# KRAS signaling in malignant pleural mesothelioma

**DOI:** 10.15252/emmm.202013631

**Published:** 2021-12-13

**Authors:** Antonia Marazioti, Anthi C Krontira, Sabine J Behrend, Georgia A Giotopoulou, Giannoula Ntaliarda, Christophe Blanquart, Hasan Bayram, Marianthi Iliopoulou, Malamati Vreka, Lilith Trassl, Mario A A Pepe, Caroline M Hackl, Laura V Klotz, Stefanie A I Weiss, Ina Koch, Michael Lindner, Rudolph A Hatz, Juergen Behr, Darcy E Wagner, Helen Papadaki, Sophia G Antimisiaris, Didier Jean, Sophie Deshayes, Marc Grégoire, Özgecan Kayalar, Deniz Mortazavi, Şükrü Dilege, Serhan Tanju, Suat Erus, Ömer Yavuz, Pınar Bulutay, Pınar Fırat, Ioannis Psallidas, Magda Spella, Ioanna Giopanou, Ioannis Lilis, Anne‐Sophie Lamort, Georgios T Stathopoulos

**Affiliations:** ^1^ Comprehensive Pneumology Center (CPC) and Institute for Lung Biology and Disease (iLBD) Helmholtz Center Munich‐German Research Center for Environmental Health (HMGU) and Ludwig‐Maximilian‐University (LMU) Munich Munich Germany; ^2^ Laboratory for Molecular Respiratory Carcinogenesis Department of Physiology Faculty of Medicine University of Patras Rio Greece; ^3^ German Center for Lung Research (DZL) Gießen Germany; ^4^ Université de Nantes CNRS INSERM CRCINA Nantes France; ^5^ Department of Pulmonary Medicine Koc University School of Medicine Istanbul Turkey; ^6^ Koc University Research Center for Translational Medicine (KUTTAM) Koc University School of Medicine Istanbul Turkey; ^7^ Center for Thoracic Surgery Munich Ludwig‐Maximilian‐University (LMU) Munich and Asklepios Medical Center Gauting Germany; ^8^ Department of Medicine V University Hospital Ludwig‐Maximilian‐University (LMU) Munich Munich Germany; ^9^ Lung Bioengineering and Regeneration Department of Experimental Medical Sciences Lund Stem Cell Center Wallenberg Molecular Medicine Center Faculty of Medicine Lund University Lund Sweden; ^10^ Department of Anatomy Faculty of Medicine University of Patras Rio Greece; ^11^ Laboratory for Pharmaceutical Technology Department of Pharmacy School of Health Sciences University of Patras Rio Greece; ^12^ Foundation for Research and Technology Hellas Institute of Chemical Engineering FORTH/ICE‐HT Rio Greece; ^13^ Centre de Recherche des Cordeliers INSERM Sorbonne Université Université de Paris Functional Genomics of Solid Tumors Paris France; ^14^ Department of Thoracic Surgery Koc University School of Medicine Istanbul Turkey; ^15^ Department of Pathology Koc University School of Medicine Istanbul Turkey

**Keywords:** asbestos, BAP1, KRAS, NF2, TP53, Cancer, Respiratory System

## Abstract

Malignant pleural mesothelioma (MPM) arises from mesothelial cells lining the pleural cavity of asbestos‐exposed individuals and rapidly leads to death. MPM harbors loss‐of‐function mutations in *BAP1*, *NF2*, *CDKN2A*, and *TP53*, but isolated deletion of these genes alone in mice does not cause MPM and mouse models of the disease are sparse. Here, we show that a proportion of human MPM harbor point mutations, copy number alterations, and overexpression of *KRAS* with or without *TP53* changes. These are likely pathogenic, since ectopic expression of mutant *KRAS* ^G12D^ in the pleural mesothelium of conditional mice causes epithelioid MPM and cooperates with *TP53* deletion to drive a more aggressive disease form with biphasic features and pleural effusions. Murine MPM cell lines derived from these tumors carry the initiating *KRAS* ^G12D^ lesions, secondary *Bap1* alterations, and human MPM‐like gene expression profiles. Moreover, they are transplantable and actionable by KRAS inhibition. Our results indicate that *KRAS* alterations alone or in accomplice with *TP53* alterations likely play an important and underestimated role in a proportion of patients with MPM, which warrants further exploration.

## Graphical abstract

Human malignant pleural mesothelioma (MPM) is a devastating occupational cancer that kills more than 200,000 individuals annually. Some MPM harbor mutations of the KRAS proto‐oncogene, but their functional significance is unknown and experimental models for these MPM are not available.

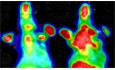

**The paper explained**

**Problem**

In a proportion of patients with human malignant pleural mesothelioma (MPM), a dreadful disease most commonly inflicted by occupational asbestos inhalation but also possibly by smoking, sporadic mutations of KRAS is observed. However, their functional impact and significance have not been addressed and experimental model systems suitable for the study of this molecular subclass of MPM are not available.

**Results**

We systematically interrogate *KRAS* alterations in the TCGA pan‐cancer dataset of human MPM and in MPM patients from our centers employing sensitive techniques. 20% of TCGA and 50% of our patients show activating mutations or amplification of *KRAS*, in 30% of the cases accompanied by *TP53* mutations or loss. These changes are associated with enhanced signaling downstream of *KRAS*. *KRAS* and *TP53* are shown to cooperate for MPM development in conditional mouse models. Three new MPM cell lines are developed that are highly similar to the human disease, and these experimental MPM models are shown to be actionable by a novel KRAS inhibitor.

**Impact**

Multiple new tools for investigations on MPM biology are provided together with proof‐of‐concept data that support involvement of *KRAS* signaling in MPM pathogenesis. The findings can be rapidly translated to clinical trials of KRAS pathway inhibition in a molecular subset of MPM patients.

## Introduction

Malignant mesothelioma annually kills up to forty persons per million population worldwide (Liu *et al*, 2017; Carbone *et al*, 2019). It most commonly arises from the mesothelium of the pleural cavities that line the lungs (visceral pleura) and the interior chest wall (parietal pleura) and only occasionally from the peritoneal mesothelium (Bibby *et al*, 2016; Mutti *et al*, 2018). Human malignant pleural mesothelioma (MPM) is mainly caused by inhaled asbestos, which caused 145,235 deaths in 1990 increasing by 51% to 218,827 deaths in 2016, most of them in high‐income countries (GBD 2016 Occupational Carcinogens Collaborators, 2020). However, other bioactive materials such as nanofibers can also cause mesothelioma in rodents and possibly in humans (Ryman‐Rasmussen *et al*, 2009; Nagai *et al*, 2011). MPM manifests with or without a malignant pleural effusion (MPE), that is, exudative fluid accumulation that causes chest pain and dyspnea, and is histologically classified into epithelioid, sarcomatoid, or biphasic subtypes (Scherpereel *et al*, 2010; Galateau‐Salle *et al*, 2016; Thomas *et al*, 2017; Paajanen *et al*, 2018). The disease progresses relentlessly despite contemporary combination therapies, with a median survival of mere 9–18 months (Zalcman *et al*, 2016; Yap *et al*, 2017; Scherpereel *et al*, 2018; Courtiol *et al*, 2019). The clinicopathologic manifestation of MPM at diagnosis impacts patient survival, with advanced stage, sarcomatoid histologic subtype, poor physical performance status, elevated numbers of peripheral blood leucocytes, male sex, uncontrolled pleural effusion, and other factors portending dismal prognosis (Fennell *et al*, 2005; Tsao *et al*, 2009; Pass *et al*, 2016; Rusch *et al*, 2016; Cheah *et al*, 2017; Thomas *et al*, 2017; Kindler *et al*, 2018; Hassan *et al*, 2019).

Multiple comprehensive analyses of MPM genomes identified a mosaic mutational landscape characterized by widespread loss‐of‐function of tumor suppressor genes (*BAP1*, *NF2*, *CDKN2A*, *TP53*, *TSC1*, etc), sporadic gain‐of‐function of proto‐oncogenes (*PIK3CA*, *EGFR*, *KRAS*, *NRAS*, *HRAS*, *BRAF*, etc), and inconclusive addiction/exclusion patterns thereof (Bott *et al*, 2011; Enomoto *et al*, 2012; Mezzapelle *et al*, 2013; Shukuya *et al*; 2014; Guo *et al*, 2015; Lo Iacono *et al*, 2015; Bueno *et al*, 2016; De Rienzo *et al*, 2016; Kato *et al*, 2016; Hmeljak *et al*, 2018). Interestingly, KRAS proto‐oncogene GTPase (*KRAS*) alterations were detected more frequently using targeted compared with massive parallel sequencing approaches by the studies above. In addition, *NF2* mutations that cause persistent KRAS signaling (Tikoo *et al*, 1994), as well as *BAP1* and *CDKN2A* mutations that are functionally related with *TP53* loss‐of‐function (Stott *et al*, 1998; Arizti *et al*, 2000; Bi *et al*, 2016), are very common in MPM. *KRAS* mutations have also been shown to activate the *TP53* cell cycle checkpoint (Matallanas *et al*, 2011). In addition to clinicopathologic presentation, MPM mutations also impact prognosis, with *TP53* and *CDKN2A* loss‐of‐function occurring more frequently in non‐epithelioid MPM and portending poor survival (Bott *et al*, 2011; Yap *et al*, 2017).

There is an unmet clinical need for mouse models that recapitulate the mutation spectrum and clinicopathologic manifestations of human MPM. In this regard, MPM cell lines for transplantable models, asbestos‐induced mouse models, and genetic models of the disease are characterized by scarcity, limited availability, and significant difficulty of implementation (Ikediobi *et al*, 2006; Fridlender *et al*, 2009; Forbes *et al*, 2015; Agalioti *et al*, 2017). Interestingly, standalone mesothelial loss‐of‐function of *BAP1*, *NF2*, *CDKN2A*, *TP53*, and *TSC1* is not sufficient to cause MPM in mice, rendering the drivers of the disease resistant to functional validation (Jongsma *et al*, 2008; Guo *et al*, 2014; Menges *et al*, 2014; Xu *et al*, 2014; Kukuyan *et al*, 2019). Moreover, faithful models of MPM are urgently needed, as most existing studies have focused on the rare peritoneal disease and only one elegant study targeted *NF2*/*CDKN2A*/*TP53* deletions to the pleural mesothelium (Jongsma *et al*, 2008). Such mouse models would represent different molecular subtypes of MPM, would have high penetrance, and would also be specific for MPM with or without MPE development.

Based on our previous observation of a *Kras* ^G12C^ mutation (*Kras*, *Mus musculus* Kirsten rat sarcoma viral oncogene homolog) in an asbestos‐induced murine MPM cell line (Agalioti *et al*, 2017; Marazioti *et al*, 2018), on published work that showed RAS pathway activation in MPM (Patel *et al*, 2007), and on the functional interconnection between mutant *KRAS* and *TP53* signaling (Matallanas *et al*, 2011), we hypothesized that *KRAS* alterations are involved in MPM development, alone or in accomplice with *TP53* alterations. Indeed, here we query the TCGA MPM dataset and employ sensitive methods in our own clinical cohorts to discover *KRAS* and *TP53* alterations in a subset of patients with MPM. We further show that targeting oncogenic *KRAS* ^G12D^ alone to the murine pleural mesothelium causes MPM and, when combined with *Trp53* deletion, triggers aggressive MPM with MPE. Murine MPM is shown to carry the initiating *KRAS* ^G12D^ mutations, to harbor *Bap1* inactivating mutations, to be transmissible to naïve mice, and to resemble the molecular signatures of human MPM. Hence, *KRAS* mutations are implicated in MPM pathobiology, the contributions of *TP53* in shaping the disease's manifestations are described, and new mouse models are provided for the study of the biology and therapy of a molecular subclass of MPM that is driven by *KRAS* signaling.

## Results

### *KRAS* and *TP53* alterations in human MPM

**Figure 1:**
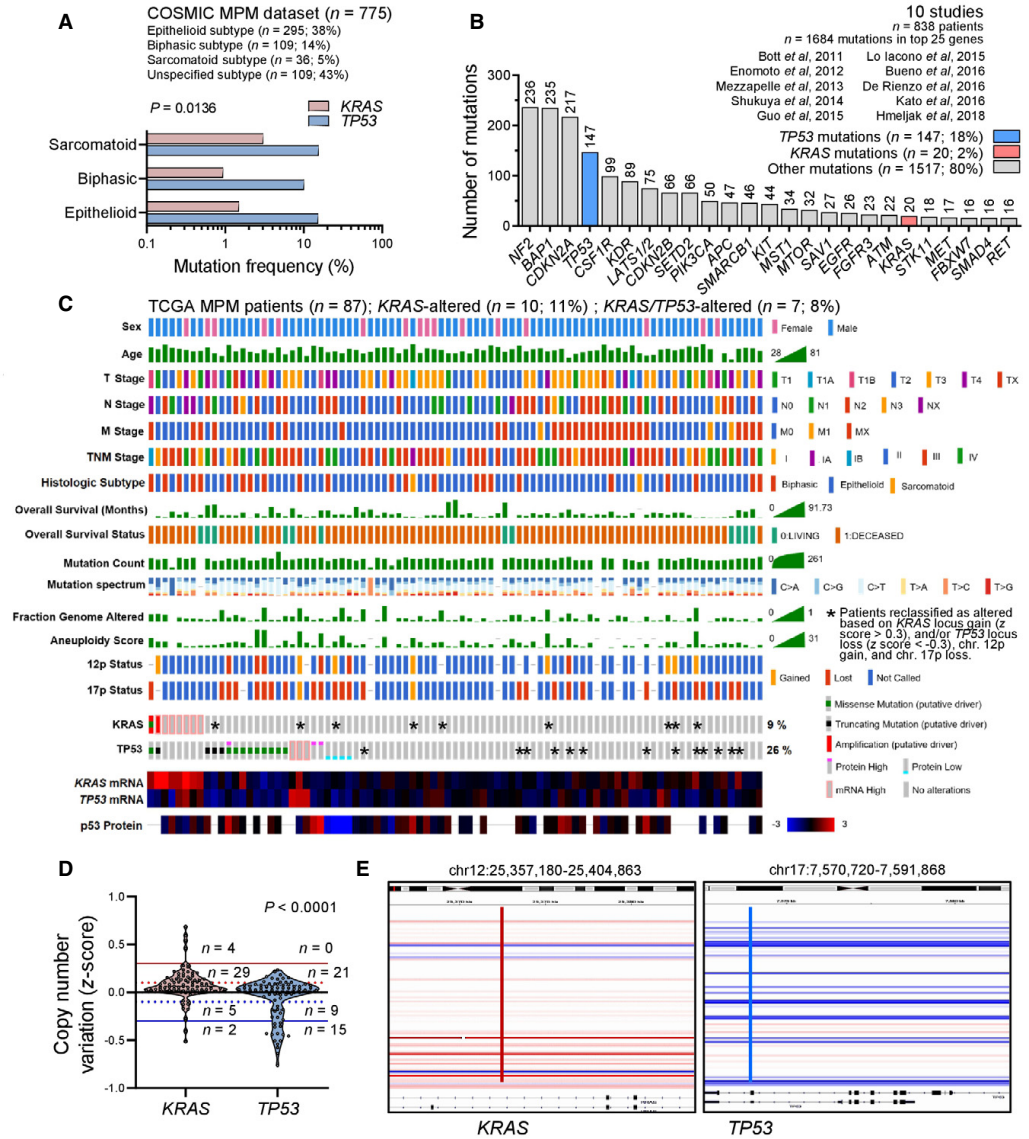
*KRAS* alterations in human MPM from published datasets and the cancer genome atlas (TCGA) pan‐cancer MPM cohort A
*KRAS* and *TP53* mutation frequencies in MPM from the catalogue of somatic mutations in cancer (COSMIC) stratified by histologic subtype (*n* = 775 patients).BTop 25 mutated genes from 10 molecular studies of human MPM (*n* = 838 patients).C–E
*KRAS* and *TP53* alterations in the cancer genome atlas (TCGA) pan‐cancer MPM dataset (*n* = 86 patients). Shown are clinical and molecular data plot with alteration frequencies (C) and patients reclassified as *KRAS‐* or *TP53*‐altered (asterisks), copy number variation data summary (D), and segments of the *KRAS* and *TP53* loci (E). Data information: In (A), data are presented as cumulative percentages of patients tested mutant respective to patients tested for every gene. *P*, overall probability, two‐way ANOVA. In (B), data are presented as cumulative numbers (*n*; numbers above bars) and percentages (%) of patients with *KRAS* (red bar), *TP53* (blue bar), and other (gray bars) mutations. In (C), each column represents one patient and each row one clinical or molecular feature. Asterisks indicate *KRAS* and *TP53* alterations not identified by the TCGA, but reclassified as altered in this study due to 12p gain, 17p loss, *KRAS* locus gain (*z* > 0.3), and/or *TP53* locus loss (*z* < −0.3). In (D), data are presented as raw data points (circles), rotated kernel density distributions (violins), and patient numbers (*n*) between thresholds of normal (solid black line at *z* = 0), low amplification (dotted red line at *z* = 0.1), low loss (dotted blue line at *z* = −0.1), high amplification (solid red line at *z* = 0.3), and deep loss (solid blue line at *z* = −0.3). *P*, probability, paired Wilcoxon rank sum test. In (E), *KRAS* (red line) and *TP53* (blue line) loci segments of all 87 patients are shown. Each horizontal segment represents one patient. White and shades of red and blue indicate no change and magnitude of gain and loss, respectively. Source data are available online for this figure.

In MPM from the catalogue of somatic mutations in cancer (COSMIC; Forbes *et al*, 2015), *KRAS* and *TP53* mutation frequencies of 1–3% and 10–20%, respectively, were evident (Fig 1A; dataset available at https://cancer.sanger.ac.uk/cosmic/browse/tissue?wgs=off&sn=pleura&ss=all&hn=mesothelioma&sh=&in=t&src=tissue&all_data=n). *KRAS* and *TP53* mutations comprised, respectively, 2 and 18% of all mutated genes in a dataset composed of 10 large MPM studies (Bott *et al*, 2011; Enomoto *et al*, 2012; Mezzapelle *et al*, 2013; Shukuya *et al*, 2014; Guo *et al*, 2015; Lo Iacono *et al*, 2015; Bueno *et al*, 2016; De Rienzo *et al*, 2016; Kato *et al*, 2016; Hmeljak *et al*, 2018) (Fig 1B). The aforementioned analysis consisted of manual curation of the main and supplementary data, while the latter study, the cancer genome atlas (TCGA) pan‐cancer MPM dataset (*n* = 86 patients; Hmeljak *et al*, 2018) available at https://www.cbioportal.org/study/summary?id=meso_tcga_pan_can_atlas_2018 (Cerami *et al*, 2012), was analyzed in detail, via a systematic query of mutations, copy number alterations, and mRNA and protein expression of *KRAS* and *TP53*. According to TCGA criteria, eight patients showed alterations in *KRAS* two of which had dual *KRAS*/*TP53* changes. However, when copy number alterations (CNA) at the *KRAS*12p12.1 (position chr12:25,357,180–25,404,863) and *TP53* 17p13.1 (position chr17:7,570,720–7,591,868) loci were scrutinized using integrative genomics viewer (Robinson *et al*, 2011), additional high *KRAS* gains were discovered in nine and deep *TP53* losses in 13 patients, with five patients harboring changes in both genes (Fig 1C–E). For this, *KRAS* locus gain (*z* > 0.3) and/or *TP53* locus loss (*z* < −0.3), as well as chromosome 12p gains and 17p losses, were taken into account (Smith & Sheltzer, 2018). Hence, a *KRAS* alteration alone was determined in *n* = 10 patients (12%) and a combined *KRAS*/*TP53* alteration in *n* = 7 (8%), for a total *KRAS* alteration rate of 20%.

**Figure 2:**
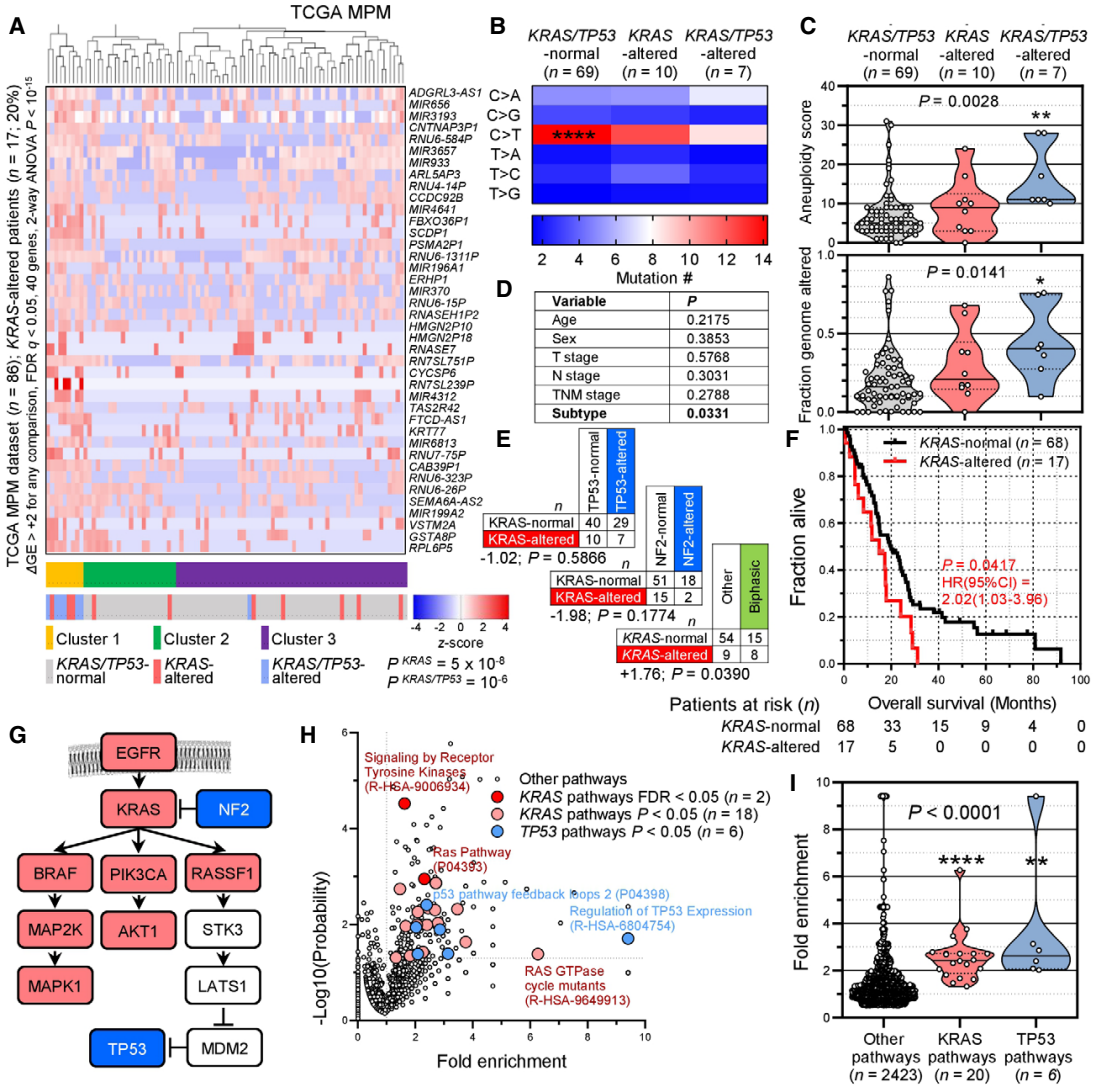
KRAS pathway activation in MPM from the cancer genome atlas (TCGA) pan‐cancer MPM dataset A–FMolecular and clinical features of the cancer genome atlas (TCGA) pan‐cancer MPM patients (*n* = 87) stratified by the presence of *KRAS* standalone (*n* = 10) and combined *KRAS*/*TP53* (*n* = 7) alterations. Shown are unsupervised hierarchical clustering of *n* = 86 patients (gene expression data were not available for one patient) by 40 genes significantly overexpressed in *KRAS*/*TP53*‐altered over *KRAS*‐altered over *KRAS*/*TP53*‐normal patients (A) and data summaries of mononucleotide change signatures (B), of indices of genomic instability and mutation burden (C), of clinical features and *KRAS*/*TP53*/*NF2* co‐mutation frequency (D, E), and of overall survival (F).GKRAS/TP53 pathway adapted from Matallanas *et al* (2011) and Tikoo *et al* (1994). Color‐coded genes were identified by TCGA and PANTHER pathway analyses.H, IPANTHER and Reactome KRAS and TP53 pathways significantly altered in the cancer genome atlas (TCGA) pan‐cancer MPM patients. Shown are volcano plot of fold‐enrichment versus −log_10_(probability) (H), and data summary of fold‐enrichment of KRAS and TP53 versus all other pathways with fold‐enrichment > 0.5 (I). Data information: In (A), data are presented as heatmap of 40 differentially expressed genes (rows) in 86 individual patients (columns), color code of unsupervised hierarchical clusters, *KRAS*/*TP53* status, and heatmap (legend), and probabilities (*P*) for enrichment of *KRAS*‐ and *KRAS*/*TP53*‐altered patients in cluster 1. The scale bar represents the color‐coded *z*‐scores. In (B), data are presented as heatmap of six different possible mononucleotide changes (rows) in patients grouped by *KRAS*/*TP53* status (columns) and color code of mean mutation number (legend). ****, FDR *q* < 2 × 10^−7^ compared with all other mononucleotide changes, 2‐way ANOVA with Benjamini, Krieger, and Yekutieli two‐stage linear step‐up procedure. In (C) and (I), data are presented as raw data points (circles), rotated kernel density distributions (violins), medians (solid lines), and quartiles (dotted lines). *P*, overall probability, Kruskal–Wallis test. (C): * and **: *P* < 0.05 and *P* < 0.01, respectively, compared with *KRAS*/*TP53*‐normal patients, Dunn's post‐tests. (I): ** and ****: *P* < 0.01 and *P* < 0.0001, respectively, compared with other pathways, Dunn's post‐tests. In (D) and (E), data are presented as patient numbers (*n*) and overall probability (*P*) by *χ* ^2^ or Kruskal–Wallis tests (D) or hypergeometric test for enrichment of *KRAS* mutations in *TP53*‐altered or biphasic MPM (E). In (F), data are presented as sample size (*n*), Kaplan–Meier survival estimates (lines), censored observations (line marks), log‐rank *P* value, and hazard ratio (HR) with 95% confidence interval (95% CI). In (H), data are presented as color‐coded individual pathways (circles), threshold of significance (horizontal dotted line), no enrichment baseline reference (vertical dotted line), and selected pathway names and codes. P and R initials in pathway codes denote PANTHER and Reactome pathways, respectively. *n*, sample size; FDR *q*, probability, false discovery rate; *Δ*GE, differential gene expression. Source data are available online for this figure.

We subsequently examined the transcriptomes of TCGA MPMs (available at https://xenabrowser.net/datapages/?dataset=TCGA‐MESO.htseq_fpkm‐uq.tsv&host=https%3A%2F%2Fgdc.xenahubs.net&removeHub=https%3A%2F%2Fxena.treehouse.gi.ucsc.edu%3A443) stratified by the presence of a *KRAS* alteration alone (*n* = 10), a combined *KRAS*/*TP53* alteration (*n* = 7), or none of the above (*n* = 69). Forty genes were biologically and statistically significantly overrepresented in *KRAS*/*TP53*‐altered over *KRAS*‐altered over normal patients, which were able to cluster patients by genetic alteration in an unsupervised hierarchical fashion (Fig 2A). *KRAS*/*TP53*‐altered patients showed loss of a C>T mononucleotide signature that preponderated in *KRAS*/*TP53*‐normal patients and displayed higher aneuploidy and genome alteration indices (Figs 2B and C). *KRAS* and *TP53* alterations were co‐occurring at a rate expected by chance, while *KRAS*‐altered patients displayed a non‐significant repulsion of *NF2* mutations, a statistically significant preponderance of biphasic histology, and significantly worse prognosis (Figs 2D–F). Interestingly, when all mutated genes from this cohort were entered into the Protein Analysis Through Evolutionary Relationships System (PANTHER; http://www.pantherdb.org/), multiple KRAS and TP53 signaling pathways were biologically and statistically significantly enriched in MPM, which, together with the *KRAS*‐*NF2* repulsion described above, aligned along a biological KRAS‐TP53 pathway proposed elsewhere (Tikoo *et al*, 1994; Matallanas *et al*, 2011) (Fig 2G–I). Our results were concordant with the TCGA pan‐cancer pathway analysis that reported 9 and 21% alteration frequencies of the RTK/RAS and p53 pathways in MPM (Sanchez‐Vega *et al*, 2018). Hence, we describe a molecular subclass of MPM patients in the TCGA dataset that involves ∼ 20% of patients, which harbor *KRAS* gain‐of‐function with or without *TP53* loss‐of‐function. This molecular MPM subset features KRAS pathway activation, different mutation spectra, gene expression profiles, histology, and survival compared to other MPMs.

**Figure 3:**
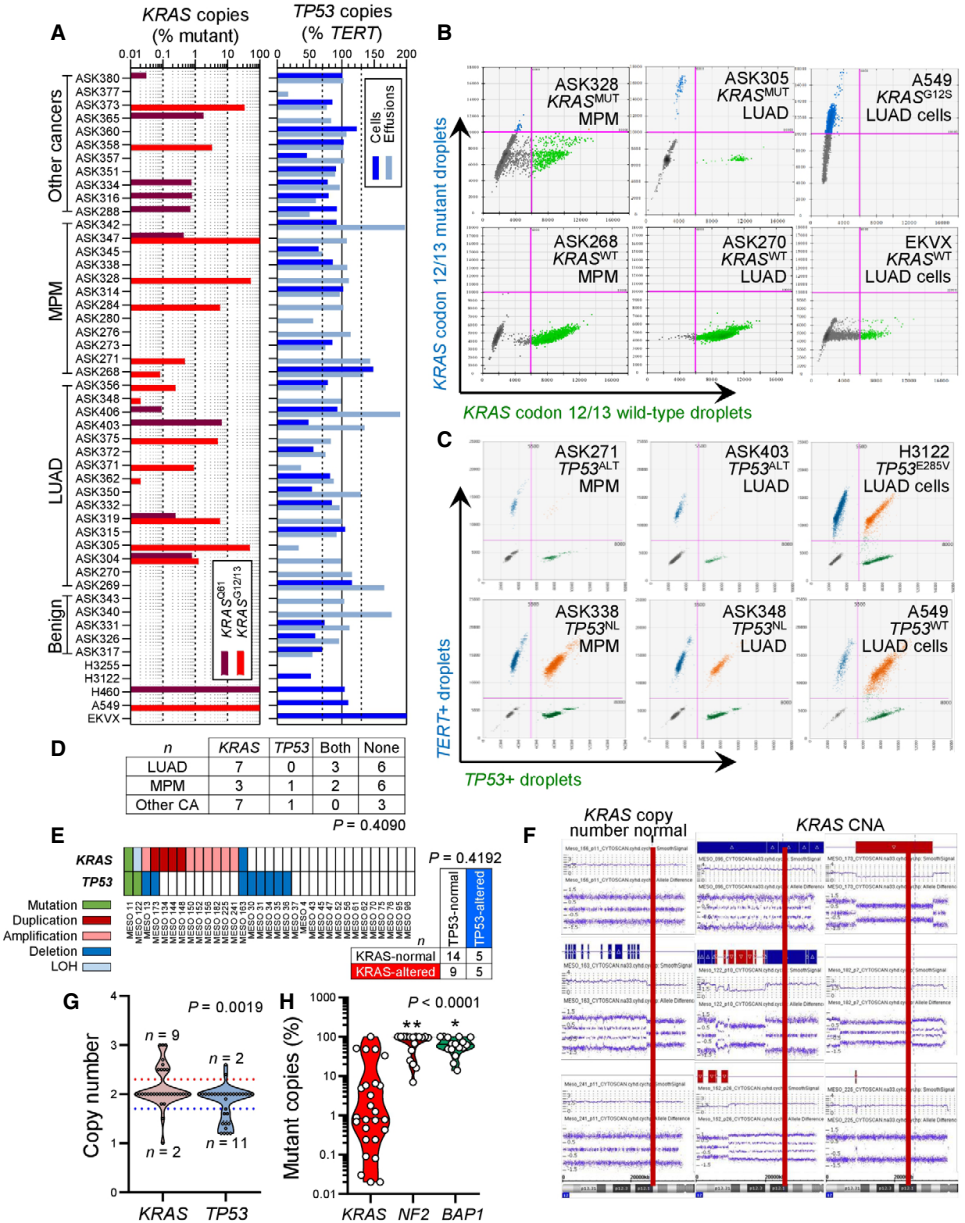
*KRAS* and *TP53* alterations in human MPM from Germany and human MPM cell lines from France A–DPleural fluid cell pellets and supernatants from 45 patients (called ASK #) with pleural effusion from Munich, Germany (Klotz *et al*, 2019a, 2019b), were subjected to digital droplet polymerase chain reaction (ddPCR) for the detection of mutant (^MUT^) copies of *KRAS* codon 12/13 (*KRAS* ^G12/13^) and *KRAS* codon 61 (*KRAS* ^Q61^), as well as copies of *TP53* and *TERT*. Diagnoses were benign pleural effusion (*n* = 5), lung adenocarcinoma (LUAD; *n* = 16), MPM (*n* = 12), and other extrathoracic cancers (*n* = 12). The assays were designed for detection of down to 1:20,000 copies using EKVX (*KRAS* ^WT^ *TP53* ^G610T^), A549 (*KRAS* ^G12S^ *TP53* ^WT^), NCI‐H460 (*KRAS* ^Q61H^ *TP53* ^WT^), NCI‐H3122 (*KRAS* ^WT^ *TP53* ^E285V^), and NCI‐H3255 (*KRAS* ^WT^ *TP53* ^G560‐1A^) human LUAD cells as controls. Shown are individual patient (*KRAS* plot) and individual sample (*TP53* plot) allelic frequencies with color code and limits of normal *TP53* allelic frequency as vertical dashed lines in the *TP53* plot (A), representative gated dotplots of codon 12/13 *KRAS* ddPCR (B) and *TP53*/*TERT* (C), and results summary table (D). Any number of *KRAS*‐mutant droplets detected in any sample (*KRAS* plot in A) and any patient that failed to achieve normal *TP53* ploidy by any sample (*TP53* plot in A) was deemed altered.E–GResults summary (E), representative *KRAS* CNA segments (F), and data summary of individual cell line CNA *z*‐score (G) from Affymetrix CytoScanHD Arrays of 33 primary MPM cell lines (called MESO #) from Nantes, France (GEO dataset GSE134349). Red lines denote the KRAS locus on chromosome 12p12.1.HData summary of mutant allelic frequency of *KRAS* compared with *NF2* and *BAP1* in all mutated samples from (A–G). Data information: In (A), data are presented as data summary of the highest mutant copy percentage detected per individual sample (*KRAS* plot) or of all individual samples assessed (*TP53* plot). In (D), data are presented as number of patients (*n*). *P*, probability, hypergeometric test for enrichment of *KRAS* mutations in MPM versus other tumors. In (E), data are presented as individual cell lines (columns), genes (rows), legend, and number of patients (*n* in table). *P*, probability, hypergeometric test for enrichment of *KRAS* mutations in *TP53*‐mutant MPM. In (G), data are presented as raw data points (circles), rotated kernel density distribution (violins), and cell line numbers (*n*) outside thresholds of amplification (dotted red line at 2.3) and loss (solid blue line at 1.7). *P*, probability, paired Wilcoxon rank sum test. In (H), data are presented as raw data points (circles), rotated kernel density distributions (violins), and medians (lines). *P*, overall probability, one‐way ANOVA. * and **: *P* < 0.05 and *P* < 0.01, respectively, compared with *KRAS*, Tukey's post‐test. Source data are available online for this figure.

**Figure EV1:**
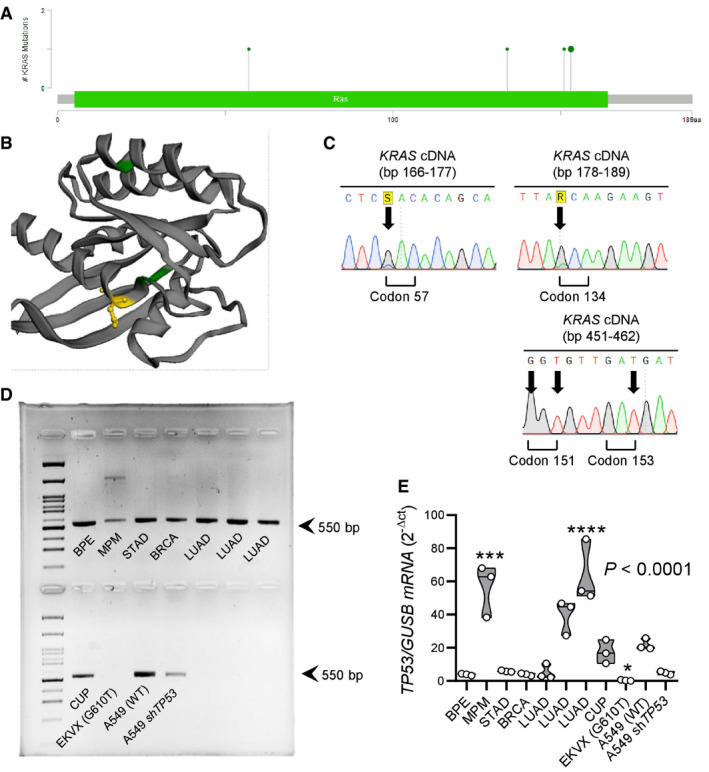
KRAS and TP53 alterations in a patient with malignant pleural mesothelioma from the MAPED study (NCT03319472) Lollipop plot showing the four different missense *KRAS* mutations found (D57H, A134T, R151G, and E153D).3D rendering of KRAS protein showing the three *KRAS* mutations predicted by OncoKB to be non‐functional (green color) and the E153D mutation predicted by OncoKB to be oncogenic (yellow color).Representative Sanger sequencing traces. Arrows indicate point mutations.
*TP53* RT–PCR in comparison to cancer cell lines and other patients with malignant pleural effusion. Note the decreased band intensity in the patient with MPM.
*TP53* qPCR in comparison to cancer cell lines and other patients with malignant pleural effusion. Note the markedly increased *TP53* transcript abundance in the patient with MPM that, together with (D), indicates a *TP53* mutation. Data information: In (A), the likely oncogenic E153D mutation is shown enlarged compared with the other three mutations. In (D), arrows at 550 base pairs (bp) indicate amplicon size. In (E), data are presented as raw data points (circles), rotated kernel density distributions (violins), and medians (lines). *P*, overall probability, one‐way ANOVA. *, ***, and ****: *P* < 0.05, *P* < 0.001, and *P* < 0.0001 compared with A549 cells, Bonferroni post‐tests. In (D) and (E), abbreviations are as follows: BPE, benign pleural effusion; MPM, malignant pleural mesothelioma; STAD, stomach adenocarcinoma; BRCA, breast cancer; LUAD, lung adenocarcinoma; CUP, cancer of unknown primary; sh, cell line stably expressing anti‐*TP53* short hairpin RNA. The TP53 status of the EKVX and A549 cell lines is indicated in parentheses. Source data are available online for this figure.

**Figure 4:**
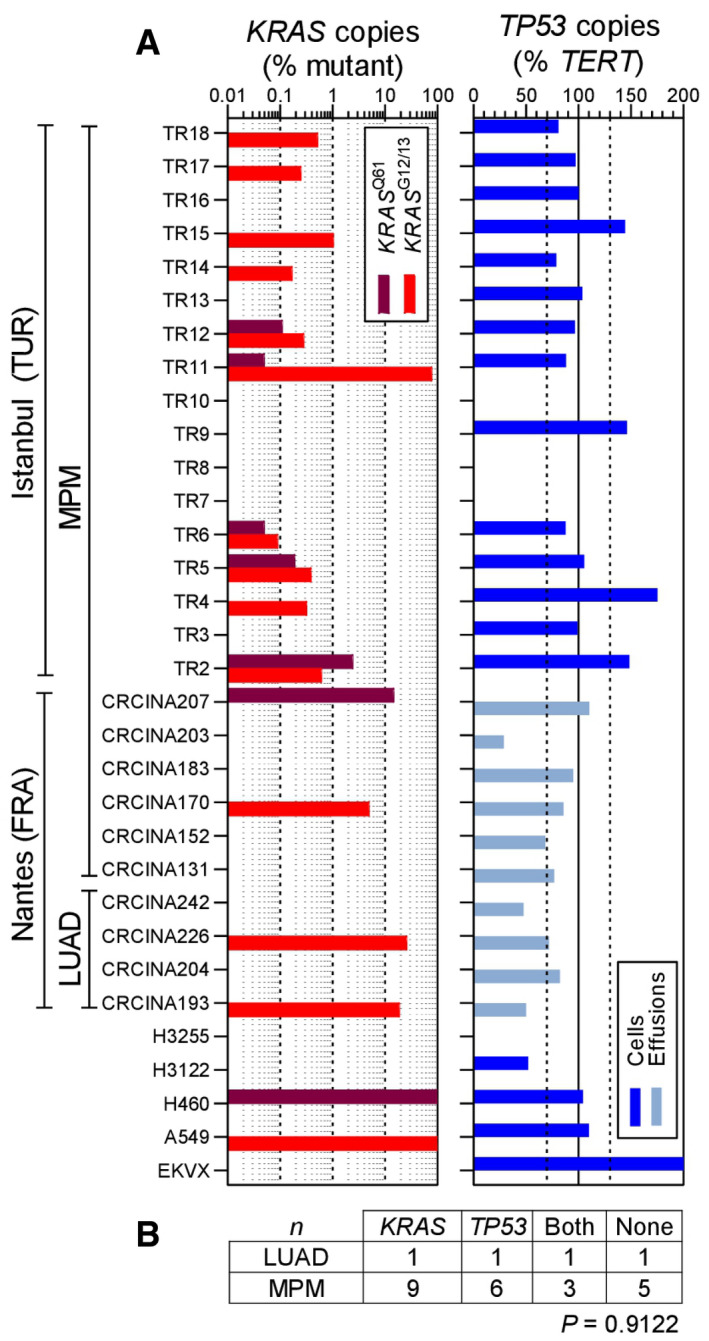
*KRAS* and *TP53* alterations in MPM patients from France and Turkey A, BPleural fluid cell pellets and supernatants from 10 patients (called CRCINA #) with pleural effusion from Nantes, France (Gueugnon *et al*, 2011; Smeele *et al*, 2018), and pleural tumor samples from 17 patients (called TR#) with MPM from Istanbul, Turkey, were subjected to digital droplet polymerase chain reaction (ddPCR) for the detection of mutant (^MUT^) copies of *KRAS* codon 12/13 (*KRAS* ^G12/13^) and *KRAS* codon 61 (*KRAS* ^Q61^), as well as copies of *TP53* and *TERT*. Diagnoses were lung adenocarcinoma (LUAD; *n* = 4) and MPM (*n* = 23). The assays were designed for detection of down to 1:20,000 copies using EKVX (*KRAS* ^WT^ *TP53* ^G610T^), A549 (*KRAS* ^G12S^ *TP53* ^WT^), NCI‐H460 (*KRAS* ^Q61H^ *TP53* ^WT^), NCI‐H3122 (*KRAS* ^WT^ *TP53* ^E285V^), and NCI‐H3255 (*KRAS* ^WT^ *TP53* ^G560‐1A^) human LUAD cells as controls. Shown are individual patient (*KRAS* plot) and individual sample (*TP53* plot) allelic frequencies with color code and limits of normal *TP53* allelic frequency as vertical dashed lines in the *TP53* plot (A) and results summary table (B). Any number of *KRAS*‐mutant droplets detected in any sample (*KRAS* plot in A) and any patient that failed to achieve normal *TP53* ploidy by any sample (*TP53* plot in A) was deemed altered. Data information: In (A), data are presented as data summary of the highest mutant copy percentage detected per individual sample (*KRAS* plot) or of all individual samples assessed (*TP53* plot). In (B), data are presented as number of patients (*n*). *P*, probability, *χ* ^2^ test. Source data are available online for this figure.

To further test this, we interrogated *KRAS* and *TP53* in our MPM patients, whose clinical characteristics are given in Appendix Table S1. We employed digital droplet polymerase chain reaction (ddPCR) in order to detect *KRAS* codon 12/13 and 61 mutations, as well as *TP53* CNA in pleural fluid and cell pellets of 45 patients with pleural effusions from our cohorts in Munich, Germany (Klotz *et al*, 2019a, 2019b). The effusions were caused from benign etiologies (*n* = 5), MPM (*n* = 12), metastatic lung adenocarcinoma (LUAD; *n* = 16), or metastatic other bodily tumors (*n* = 12). The assays were designed for the detection of down to 1:20,000 mutant (^MUT^) or wild‐type (^WT^) copies. We detected standalone *KRAS* mutations and combined *KRAS*/*TP53* alterations in three and two of our 12 patients with MPM, respectively (Fig 3A–C). *KRAS* and *TP53* alterations co‐occurred at a rate expected by chance (Fig 3D). We next used sensitive Affymetrix CytoScanHD Arrays utilizing 2.67 million markers and targeted next‐generation sequencing to identify *KRAS* and *TP53* alterations in a cohort of 33 primary MPM cell lines from Nantes, France (GEO dataset GSE134349; Gueugnon *et al*, 2011; Data ref: Blanquart *et al*, 2019; Delaunay *et al*, 2020; Quetel *et al*, 2020) The clinical characteristics of the cell line donors are given in Appendix Table S2. We detected standalone *KRAS* and combined *KRAS*/*TP53* alterations in nine and five cell lines, respectively, and *KRAS* and *TP53* alterations again co‐occurred at a rate expected by chance (Fig 3E). In addition, the *KRAS* and *TP53* loci were statistically significantly amplified and deleted, respectively, across all cell lines irrespective of genotype (Fig 3F and G). Interestingly, 80% of the samples with *KRAS* ^MUT^ copies from both studies displayed low mutant copy numbers (< 10%) that would be likely missed by other techniques with lower read depths or stringent detection thresholds (Fig 3H). We also tested a patient with MPM from the Malignancy of Pleural Effusions in the Emergency Department (MAPED; ClinicalTrials.gov # NCT03319472) Study (preprint: Marazioti *et al*, 2021) for *KRAS* and *TP53* status by Sanger sequencing, RT–PCR, and qPCR. We found four different KRAS point mutations in this patient, as well as discrepant *TP53* expression levels by RT–PCR and qPCR, strongly indicative of a *TP53* mutation (Fig EV1). To obtain definitive validation, we finally examined by ddPCR for *KRAS* codon 12/13 and 61 mutations, as well as *TP53* CNA, additional six MPM‐associated MPE samples from Nantes (Gueugnon *et al*, 2011; Smeele *et al*, 2018) and 17 MPM tumor samples from Istanbul, Turkey (patients' clinical characteristics are given in Appendix Table S3). Indeed, we found that nine patients had standalone KRAS mutations, whereas another three had combined *KRAS*/*TP53* alterations (Fig 4A and B). Taken together, we examined 36 human tumor/effusion samples from four countries to find standalone *KRAS* alterations in 12 (33%) and combined *KRAS*/*TP53* alterations in 6 (17%) patients. These results indicate that a molecular subset of MPM that is driven by *KRAS* with/without *TP53* alterations indeed exists outside the TCGA cohort.

### MPM in mice expressing mesothelial‐targeted *KRAS* ^G12D^

**Figure EV2:**
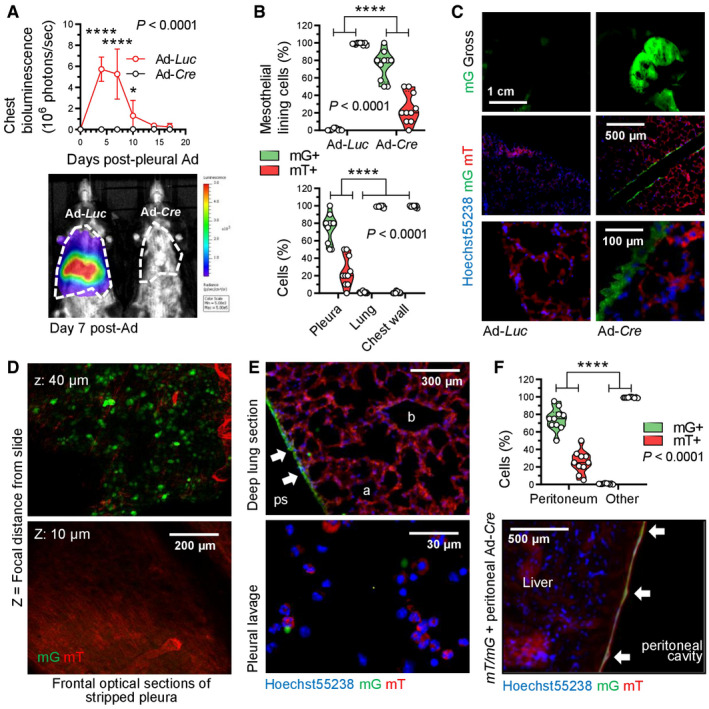
Adenoviral‐mediated mesothelial recombination Dual‐fluorescent *mT*/*mG* CRE‐reporter mice (*C57BL*/*6* background) received 5 × 10^8^ PFU intrapleural (A–E) or intraperitoneal (F) Ad‐*Luc* or Ad‐*Cre* and were serially imaged for bioluminescence.
Data summary of chest light emission (top; *n* = 5 mice/group) and representative bioluminescence images (bottom). Note cessation of transient Ad‐*Luc* expression by day 14.Data summary of mG^+^ and mT^+^ mesothelial, lung, and chest wall cell percentage (*n* = 10 mice/group; B), representative macroscopic (top) and microscopic (middle, bottom) fluorescent images (C), optical frontal sections of stripped parietal pleura placed apical side up on glass slides (D), and deep lung sections (E, top) and fluorescent image of pleural lavage cells (E, bottom). *z*, focal plane distance from slide. a, alveoli; b, bronchi; ps, pleural space; arrows, recombined mesothelium.Data summary of mG^+^ and mT^+^ mesothelial and deeper located (other) abdominal cell percentage (*n* = 10 mice/group) and representative merged microscopic fluorescent image of peritoneal surface mesothelium showing *Cre*‐recombined mesothelium (arrows). Data information: In (A), data are presented as mean ± 95% confidence interval. *P*, overall probability, two‐way ANOVA. * and ****: *P* < 0.05 and *P* < 0.0001 for comparison between groups at the indicated time points, Bonferroni post‐tests. In (B) and (F), data are presented as raw data points (circles), rotated kernel density distribution (violins), and medians (lines). *P*, overall probability, two‐way ANOVA. ****: *P* < 0.0001 for the indicated comparisons, Bonferroni post‐tests. Ad, adenovirus; PFU, plaque‐forming units; *Luc*, luciferase gene; *Cre*, CRE recombinase gene; *mT*, membranous tomato red; *mG*, membranous green fluorescent protein. Source data are available online for this figure.

**Figure EV3:**
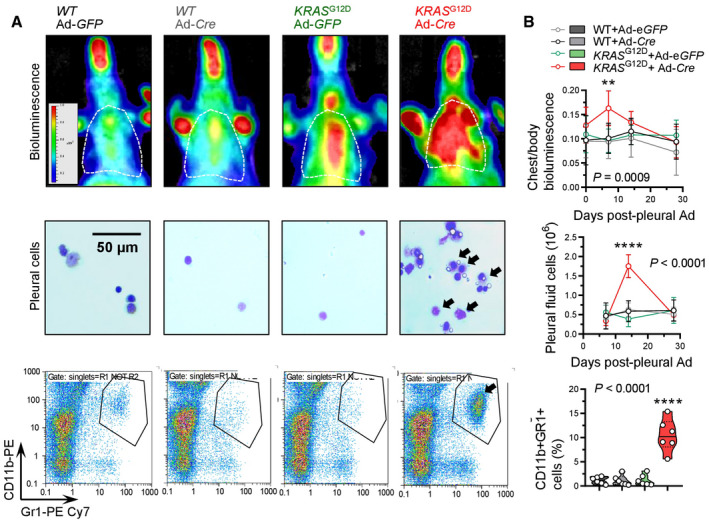
Pleural mesothelial *KRAS* ^G12D^ expression causes inflammation Wild‐type (*WT*) and *KRAS* ^G12D^ mice were lethally irradiated (1,100 Rad) and received same‐day bone marrow transfer of 10 million bone marrow cells from ubiquitously luminescent *CAG.Luc.eGFP* donors (all on the *C57BL*/*6* strain). After 1 month required for bone marrow reconstitution, chimeras received 5 × 10^8^ PFU intrapleural Ad vectors, were longitudinally imaged for bioluminescence, and were sacrificed for pleural lavage cell analysis.
Representative chest bioluminescence images taken 2 weeks post‐Ad (top), pleural lavage cytocentrifugal specimens stained with May–Gruenwald–Giemsa (middle), and dotplots of CD11b and Gr1 expression by flow cytometry (bottom). Dotted lines in top panels denote the chest. Arrows in middle and bottom panels indicate increased mononuclear cells.Summary of longitudinal chest light emission and total pleural cell number (dotplots), legend to dotplots, as well as of CD11b^+^Gr1^+^ pleural cells at day 14 post‐Ad (violin plot). Data information: In (B), data are presented as mean ± 95% confidence interval (dotplots; *n* = 5–6 mice/data‐point) or as raw data points (circles), rotated kernel density distribution (violins), and medians (lines). *P*, overall probability, one‐way (violin plot) or two‐way (dotplots) ANOVA. ** and ****: *P* < 0.01 and *P* < 0.0001, respectively, for Ad‐*Cre*‐treated *KRAS* ^G12D^ mice compared with all other groups, Bonferroni post‐tests. *WT*, wild‐type; *KRAS* ^G12D^, Lox‐STOP‐Lox.*KRAS* ^G12D^; *CAG. Luc.eGFP*, ubiquitously luminescent mice; Ad, adenovirus type 5; PFU, plaque‐forming units; *Cre*, CRE recombinase gene; GFP, green fluorescent protein; ANOVA, analysis of variance. Source data are available online for this figure.

To functionally validate *KRAS* mutations in MPM, we targeted transgenes to mesothelial surfaces using type 5 adenoviral vectors (Ad). For this, *mT*/*mG* CRE‐reporter mice that switch from somatic cell membranous tomato (mT) to green fluorescent protein (mG) expression upon *Cre*‐mediated recombination (Muzumdar *et al*, 2007) received 5 × 10^8^ plaque‐forming units (PFU) intrapleural Ad encoding *Melanotus* luciferase (Ad‐*Luc*) or *Cre* recombinase (Ad‐*Cre*) followed by serial bioluminescence imaging. Ad‐*Luc*‐treated mice developed intense bilateral chest light emission (mice lack mediastinal separations; Stathopoulos *et al*, 2006) that peaked at 4–7 and subsided by 14 days post‐injection (Fig EV2A). At this time point, when transient Ad‐*Luc* expression ceased and therefore maximal Ad‐*Cre*‐mediated recombination was achieved, Ad‐*Cre*‐treated mice displayed widespread recombination of the pleural mesothelium even in contralateral pleural fissures, but not of the lungs, chest wall, or pleural immune cells (Fig EV2B–E). Similar results were obtained from intraperitoneal 5 × 10^8^ PFU Ad‐*Cre*‐treated *mT*/*mG* mice after 2 weeks (Fig EV2F). Importantly, Ad‐*Cre* did not cause inflammation in wild‐type (*Wt*) mice, as evident by imaging and cellular analyses of luminescent bone marrow chimeras used as real‐time myeloid tracers (Cao *et al*, 2004; Giannou *et al*, 2015; Agalioti *et al*, 2017; Fig EV3). These results show that intraserosal Ad‐*Cre* treatment efficiently and specifically recombines mesothelial surfaces *in vivo*.

**Figure 5:**
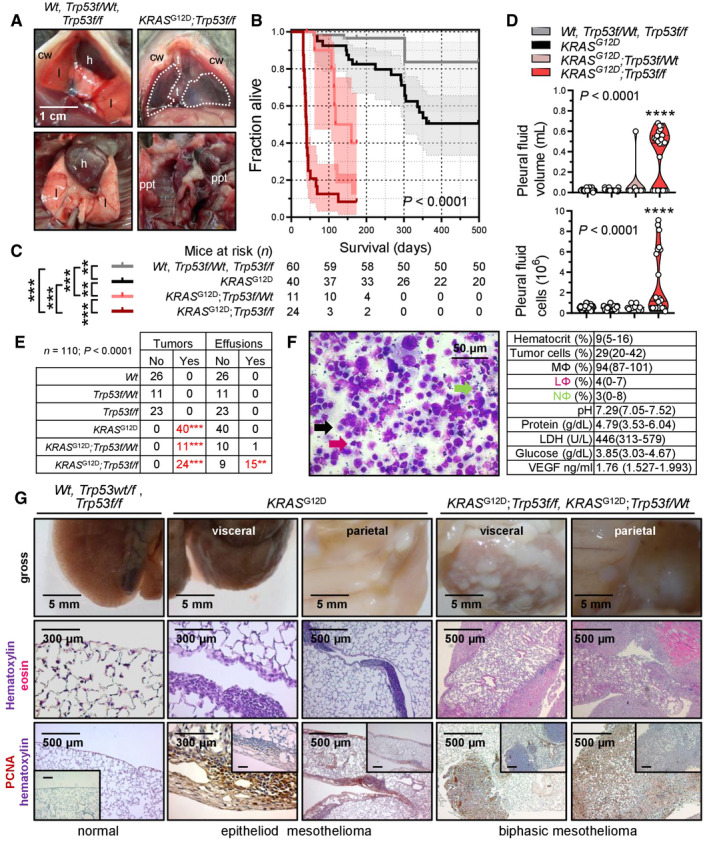
Human‐like malignant pleural mesotheliomas and effusions of mice with pleural mesothelial‐targeted oncogenic *KRAS* ^G12D^ and/or *Trp53* deletion Wild‐type (*Wt*), *KRAS* ^G12D^, and *Trp53f*/*f* mice (all *C57BL*/*6*) were intercrossed and all possible offspring genotypes received 5 × 10^8^ PFU intrapleural Ad‐*Cre* (*n* is given in survival table in [C]).
Representative photographs of the thorax before (top) and after (bottom) chest opening (t, tumors; l, lungs; cw, chest wall; h, heart; dashed lines, effusion; ppt, parietal pleural tumors).Kaplan–Meier survival plot.Survival table.Data summary of pleural effusion volume and nucleated cells (*n* is given in table in [C]).Incidence of pleural tumors and effusions.Representative May–Gruenwald–Giemsa‐stained pleural fluid cytocentrifugal specimen from a *KRAS* ^G12D^;*Trp53f*/*f* mouse showing macrophages (MΦ, black arrow), lymphocytes (LΦ, purple arrow), and neutrophils (NΦ, green arrow) and summary of cellular and biochemical features of effusions of *KRAS* ^G12D^;*Trp53f*/*f* mice (*n* = 10).Gross macroscopic and microscopic images of visceral and parietal tumors stained with hematoxylin and eosin or PCNA (*n* is given in table in [E]). Data information: In (B) and (C), data are presented as Kaplan–Meier survival estimates (lines), censored observations (line marks) 95% confidence interval (shaded areas) and number of mice at risk. *P*, overall probability, log‐rank test. ** and ***: *P* < 0.01 and *P* < 0.001, respectively, for the comparisons indicated, log‐rank test. In (D), data are presented as raw data points (circles), rotated kernel density distribution (violins), and medians (lines). *P*, overall probability, one‐way ANOVA. ****: *P* < 0.0001, for comparison with all other groups, Bonferroni post‐tests. In (E), data are presented as number of mice (*n*). *P*, probability for comparison with the top‐three groups, Fischer's exact test. In (F), data are presented as mean ± 95% confidence interval. *Wt*, wild‐type; *KRAS* ^G12D^, Lox‐STOP‐Lox.*KRAS* ^G12D^; *Trp53f*/*f*, conditional *Trp53*‐deleted; Ad, adenovirus type 5; PFU, plaque‐forming units; *Cre*, CRE recombinase gene; PCNA, proliferating cell nuclear antigen; LDH, lactate dehydrogenase; ANOVA, analysis of variance; VEGF, vascular endothelial growth factor. Source data are available online for this figure.

To test whether oncogenic *KRAS* can cause MPM, *Wt* mice and mice carrying conditional *KRAS* ^G12D^ and/or *Trp53f*/*f* alleles expressed or deleted, respectively, upon *Cre*‐mediated recombination (Marino *et al*, 2000; Jackson *et al*, 2001; Meylan *et al*, 2009) received 5 × 10^8^ PFU intrapleural Ad‐*Cre* and were longitudinally followed and sampled (Fig 5A–F). *Wt*, *Trp53f*/*Wt*, and *Trp53f*/*f* mice survived up to 16 months post‐Ad without clinical or pathologic disease manifestations (median survival undefined). In contrast, *KRAS* ^G12D^ mice developed cachexia and succumbed by 6–12 months post‐injection (median [95% CI] survival = 339 [285–379] days; *P* = 0.005 compared with controls, log‐rank test). At necropsy, no pleural fluid or inflammatory cell accumulation was evident, but diffuse visceral and parietal pleural nodular and peel‐like lesions were found in all mice. These lesions expressed proliferating cell nuclear antigen (PCNA) unlike the normal pleura and were diagnosed by a board‐certified pathologist as epithelioid MPM (Fig 5G). In addition, chimeric *KRAS* ^G12D^ recipients adoptively transplanted with luminescent bone marrow revealed an early pleural inflammatory infiltrate composed of CD11b^+^Gr1^+^ myeloid cells at 7–14 days post‐Ad‐*Cre* (Fig EV3), emulating the inflammatory response observed after pleural asbestos instillation (Nagai *et al*, 2011) that is thought to drive MPM development (Fridlender *et al*, 2009; Patil *et al*, 2018; Courtiol *et al*, 2019).

**Figure EV4:**
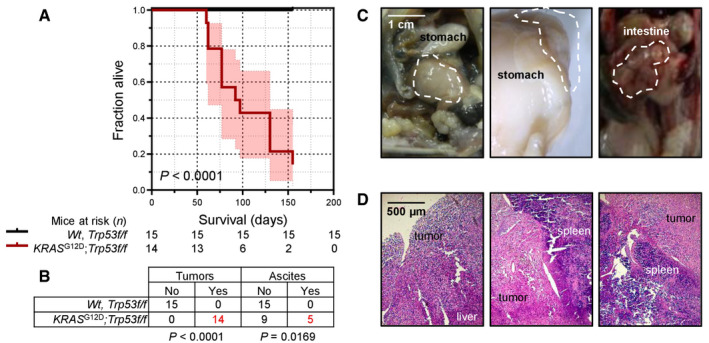
Malignant peritoneal mesothelioma of *KRAS* ^G12D^ *;Trp53f*/*f* mice Wild‐type (*Wt*), *Trp53f*/*f*, and *KRAS* ^G12D^ *;Trp53f*/*f* mice (all *C57BL*/*6*) received 5 × 10^8^ PFU intraperitoneal Ad‐*Cre* and were harvested when moribund.
Kaplan–Meier survival plot and survival table.Tumor and ascites incidence table.Representative macroscopic images of peritoneal tumors (dashed outlines).Representative hematoxylin‐and‐eosin‐stained tissue sections of peritoneal tumors. Data information: In (A), data are presented as Kaplan–Meier survival estimates (lines), 95% confidence intervals (shaded areas) and numbers of mice at risk. *P*, probability, log‐rank test. In (B), data are presented as number of mice (*n*). *P*, probabilities, Fischer's exact tests. *Wt*, wild‐type; *KRAS* ^G12D^, Lox‐STOP‐Lox.*KRAS* ^G12D^; *Trp53f*/*f*, conditional *Trp53*‐deleted; Ad, adenovirus type 5; PFU, plaque‐forming units; *Cre*, CRE recombinase gene. Source data are available online for this figure.

The phenotype of intrapleural Ad‐*Cre*‐injected *KRAS* ^G12D^ *;Trp53f*/*f* mice was fulminant, with respiratory and locomotor distress and retracted body posture culminating in death by 3–6 weeks post‐Ad‐*Cre* (median [95% CI] survival = 41 [38–73] days; *P* < 0.001 compared with any other genotype, log‐rank test). Examination of the thorax revealed massive MPE in most and visceral/parietal pleural tumors in all mice, which invaded the lungs, chest wall, and mediastinum and uniformly presented as PCNA^+^ biphasic MPM with mixed sarcomatoid/epithelioid features. Effusions were bloody but non‐coagulating, contained abundant cancer and inflammatory cells, and had low pH and glucose and high protein, VEGF, and lactate dehydrogenase levels, resembling effusions of human advanced MPM (Robinson *et al*, 2005; Patil *et al*, 2018) and of *C57BL*/*6* mice injected with *KRAS^G12C^*‐mutant AE17 mesothelioma cells (Agalioti *et al*, 2017). *KRAS* ^G12D^ *;Trp53f*/*Wt* mice displayed an intermediate phenotype (median [95% CI] survival = 118 [97–160] days; *P* < 0.003 compared with any other genotype, log‐rank test), biphasic histology, and a single MPE occurrence. *Wt*, *Trp53f*/*f*, and *KRAS* ^G12D^ *;Trp53f*/*f* mice also received 5 × 10^8^ PFU intraperitoneal Ad‐*Cre* (Fig EV4). Again, *Wt* and *Trp53f*/*f* mice displayed unlimited survival without signs of disease (median survival undefined), but K*RAS* ^G12D^ *;Trp53f*/*f* mice developed abdominal swelling and succumbed by 2–5 months post‐Ad‐*Cre* (median [95% CI] survival = 95 [60–123] days; *P* < 0.001 compared with controls, log‐rank test). At necropsy, nodular and diffuse tumors throughout the abdominal cavity and loculated ascites with features similar to MPM with MPE were detected.

**Figure 6:**
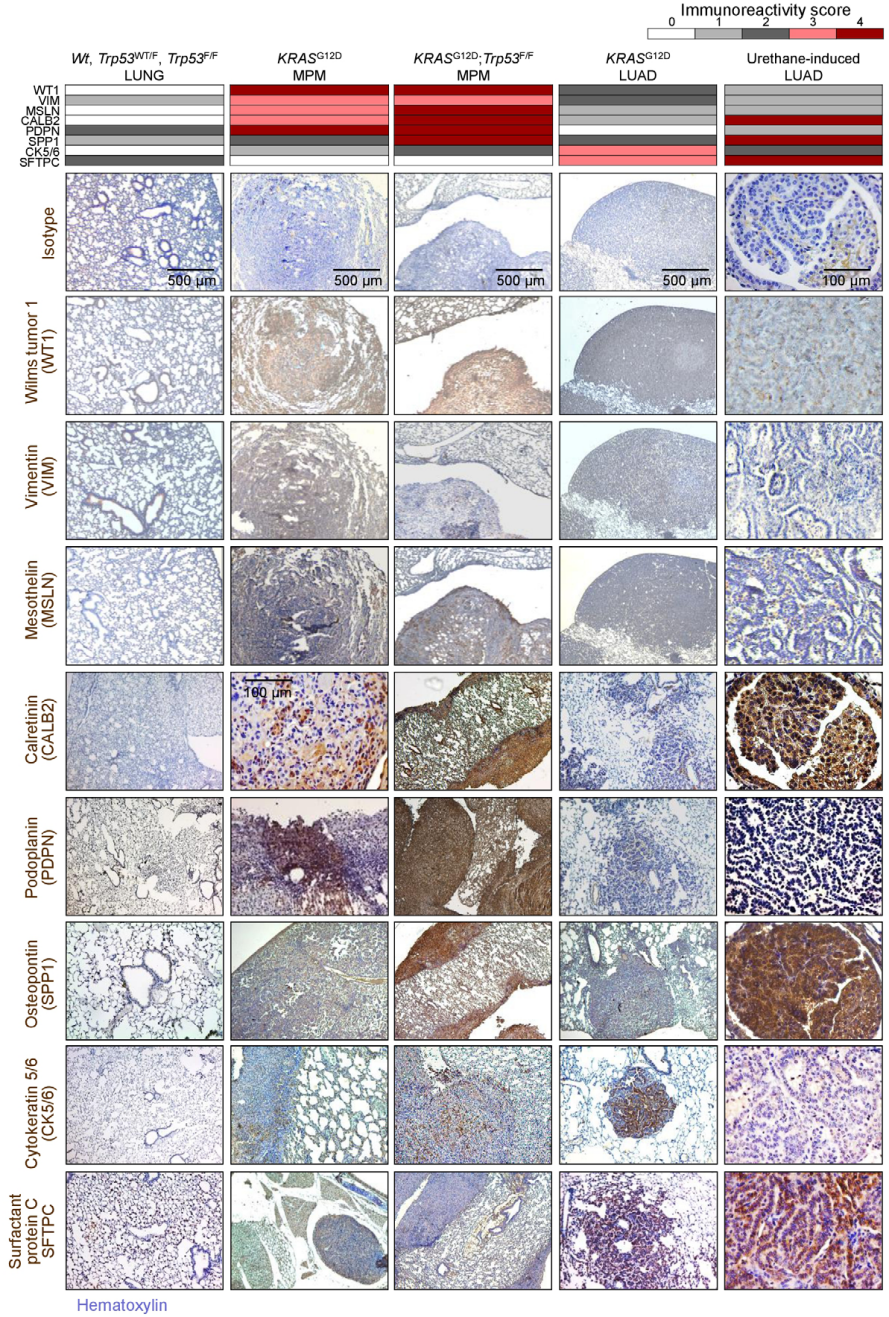
Molecular phenotyping of murine mesothelioma Wild‐type (*Wt*), *KRAS* ^G12D^, and *Trp53f*/*f* mice were intercrossed, and all possible offspring genotypes received 5 × 10^8^ PFU intrapleural or intratracheal Ad‐*Cre* and were sacrificed when moribund. In parallel, *C57BL*/*6* mice received 10 consecutive weekly intraperitoneal injections of 1 g/kg urethane and were sacrificed after 6 months. Data summary (heatmap) and representative images of immunoreactivity of tissue sections of pleural and pulmonary tissues and tumors from these mice for different markers of human malignant pleural mesothelioma (MPM) and lung adenocarcinoma (LUAD). *n* = 10 mice/group were analyzed for each marker. Brown color indicates immunoreactivity and blue color nuclear hematoxylin counterstaining. Note the ubiquitous strong expression of Wilms' tumor 1 (WT1), patchy moderate expression of vimentin (VIM), ubiquitous moderate expression of mesothelin (MSLN), ubiquitous strong expression of calretinin (CALB2), podoplanin (PDPN), and osteopontin (SPP1), patchy moderate expression of cytokeratin 5/6 (CK5/6), and the absence of expression of surfactant protein C (SFTPC) in murine *KRAS*‐driven mesotheliomas. Note also the ubiquitous strong expression of WT1, the patchy moderate expression of VIM, the ubiquitous low‐level expression of MSLN, the ubiquitous strong expression of CALB2 and SPP1, the ubiquitous low‐level expression of PDPN, the variable moderate expression of CK5/6, and the ubiquitous moderate expression of SFTPC in murine *KRAS* ^G12D^‐driven and urethane‐induced LUAD.

To corroborate that our mice had mesothelioma and not pleural spread of LUAD (Jackson *et al*, 2001), immunostaining for specific markers of both tumor types was performed based on expert guidelines for distinguishing human MPM from LUAD (Scherpereel *et al*, 2010; Galateau‐Salle *et al*, 2016; Courtiol *et al*, 2019) and on previous published experience from mouse models (Jongsma *et al*, 2008). In parallel, LUAD of intratracheal Ad‐*Cre*‐treated (5 × 10^8^ PFU) *KRAS* ^G12D^ and of urethane‐treated mice were examined (Mason *et al*, 2000; Spella *et al*, 2019). Our murine MPM displayed ubiquitous strong Wilms' tumor 1, patchy moderate vimentin, ubiquitous moderate mesothelin, ubiquitous strong calretinin/podoplanin/osteopontin, and patchy moderate cytokeratin 5/6 expression, but no evidence of surfactant protein C expression, in contrast with LUAD that expressed some of these markers and SFTPC (Fig 6), supporting that our tumors are indeed MPM of the biphasic subtype. These results show that pleural mesothelial‐targeted *KRAS* ^G12D^ causes epithelioid MPM in mice. Furthermore, that standalone *TP53* loss does not trigger MPM, but cooperates with mutant *KRAS* to accelerate MPM development, to promote biphasic histology, and to precipitate effusion formation.

### Transplantable and actionable murine MPM cell lines with *KRAS* ^G12D^, *Trp53*, and *Bap1* mutations, and a human‐like transcriptome

**Figure 7:**
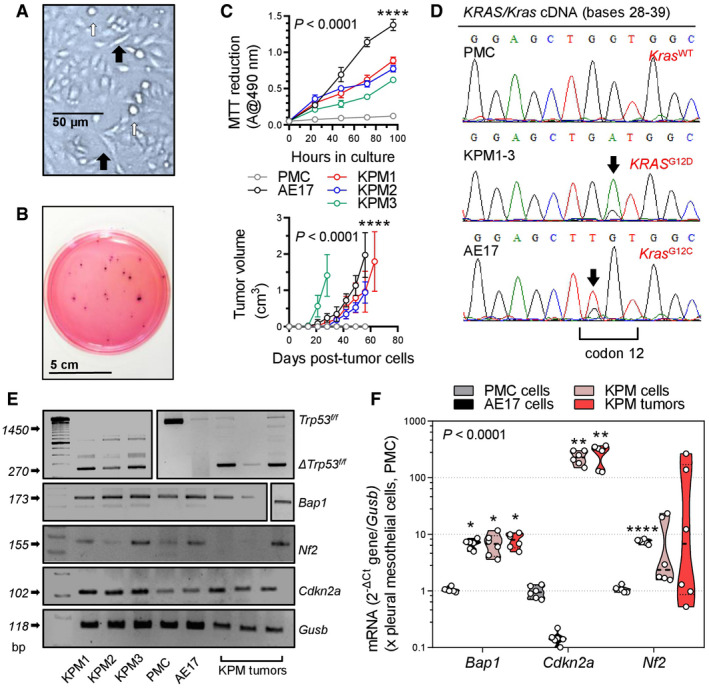
Transplantable *KRAS*/*TP53*‐mutant murine mesothelioma (KPM) cell lines *KRAS* ^G12D^ *;Trp53f*/*f* pleural mesothelioma (KPM), pleural mesothelial (PMC), and asbestos‐induced AE17 mesothelioma cells (all from *C57BL*/*6* mice) were analyzed.
AKPM cell culture showing anoikis (white arrows) and spindle‐shaped morphology (black arrows).BRepresentative colonies of KPM1 cells (7.5 × 10^3^ cells/vessel) seeded on a soft agar‐containing 60‐mm petri dish and stained with crystal violet after a month (*n* = 3/group).CData summaries from *in vitro* MTT reduction (top; 2 × 10^4^ cells/well; *n* = 6 independent experiments) and *in vivo* subcutaneous tumor growth after injection of 10^6^ cells per *C57BL*/*6* mouse (bottom; *n* = 5/group).D
*KRAS*/*Kras* mRNA Sanger sequencing shows wild‐type *Kras* (*Kras* ^WT^) of PMC and mutant murine *Kras*/human *KRAS* alleles (*KRAS* ^G12D^ and *Kras* ^G12C^) of KPM and AE17 cells (arrows).E, FRT–PCR (E) and qPCR (F) of KPM cells and parental tumors show *Trp53f*/*f* allele deletion (*Δ*) and *Bap1* and *Cdkn2a* overexpression compared with PMC. Data information: In (C), data are presented as mean (circles) and 95% confidence interval (bars). *P*, overall probability, two‐way ANOVA. ****: *P* < 0.0001 for AE17 cells (top) or for KPM cells (bottom) compared with all other groups, Bonferroni post‐tests. In (F), data are presented as raw data points (circles), rotated kernel density distribution (violins), and medians (lines). *P*, overall probability, two‐way ANOVA. *, **, and ****: *P* < 0.05, *P* < 0.01, and *P* < 0.0001, respectively, for comparison with PMC, Bonferroni post‐tests. Source data are available online for this figure.

**Figure EV5:**
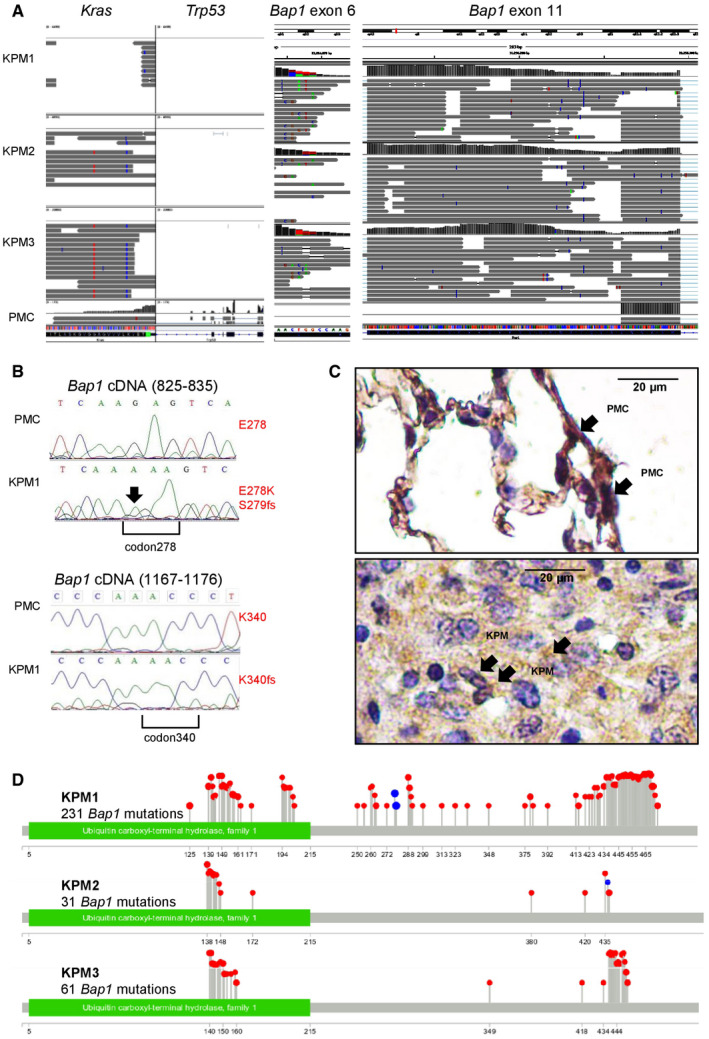
Bap1 mutations of KPM cells *KRAS* ^G12D^ *;Trp53f*/*f* pleural mesothelioma (KPM) and pleural mesothelial cells (PMC) were analyzed by RNA sequencing (GEO dataset GSE94415), Sanger sequencing for *Bap1*, and immunohistochemistry for BAP1 protein expression.
Coverage and alignment plot from RNA sequencing. Alignments are represented as gray polygons with reads mismatching the reference indicated by color. Loci with a large percentage of mismatches relative to the reference are flagged in the coverage plot as color‐coded bars. Alignments with inferred small insertions or deletions are represented with vertical or horizontal bars, respectively.
*Bap1* mRNA Sanger sequencing shows a G>A transition (arrow) at c.829 that generates a missense mutation in codon E278K (top), as well as a single nucleotide insertion in position c.831 with a consequent frameshift mutation in codon S279insA and a single nucleotide insertion resulting to a frameshift mutation in codon K340insA at c.1072 (bottom).Representative immunohistochemical images of BAP1 immunoreactivity (brown) of lungs with normal PMC and mouse tumors caused by transplanted KPM cells counterstained with hematoxylin (blue). Arrows indicate nuclear BAP1 staining.Lollipop plot for each KPM cell line visualizing all *Bap1* mutations detected. Red and blue lollipops with their numbers represent, respectively, missense mutations and insertions causing frameshift with their positions after the ATG start codon.

**Figure 8:**
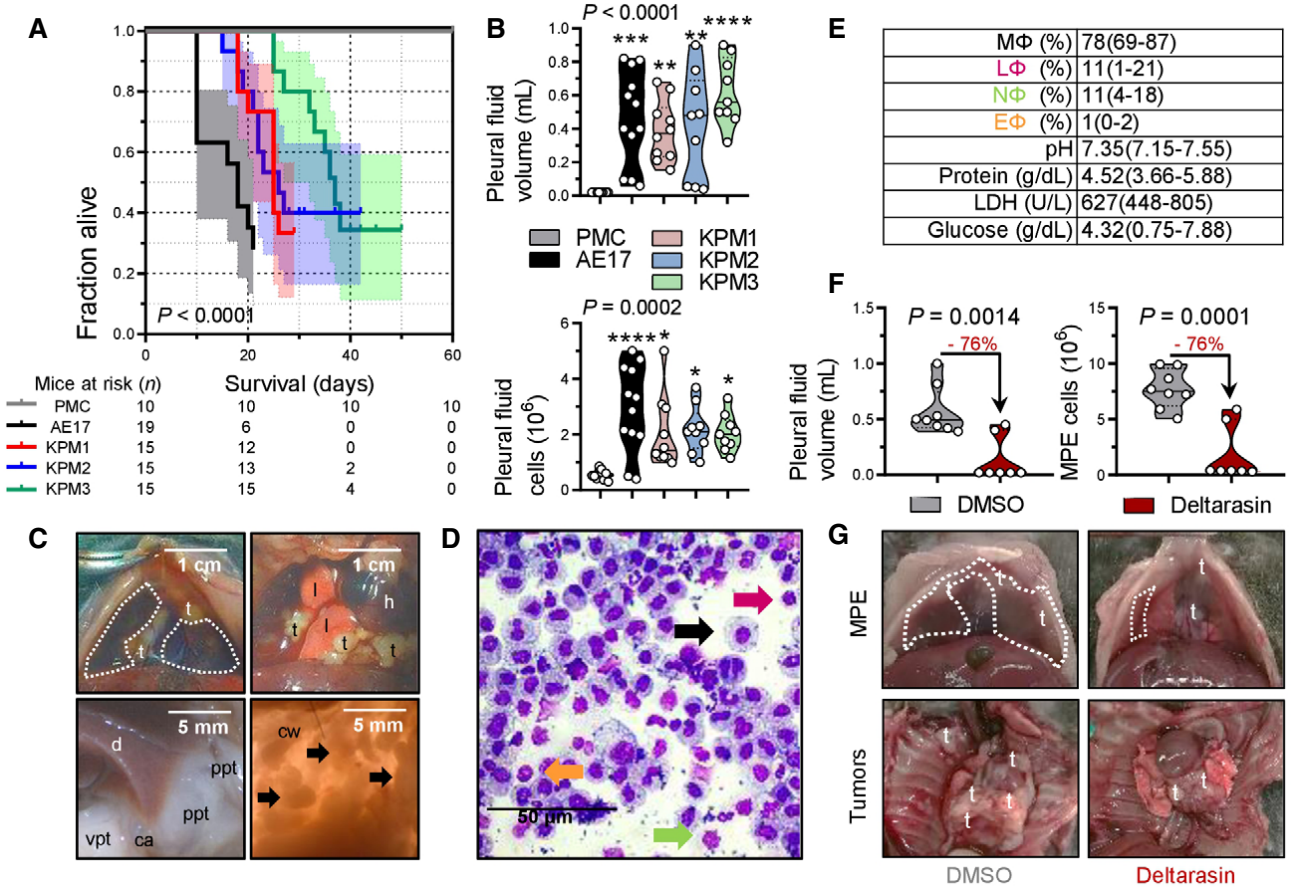
Transplantable and actionable murine mesothelioma models using KPM cells *C57BL*/*6* mice received 2 × 10^5^ intrapleural *KRAS* ^G12D^ *;Trp53f*/*f* pleural mesothelioma cells (KPM), pleural mesothelial cells (PMC), or asbestos‐induced AE17 MPM cells.
AKaplan–Meier survival plot with survival table.BData summary of pleural effusion volume and total cells (*n* = 10, 12, 10, 9, and 9 mice/group, respectively, from left to right).CImages of the chest before and after opening, showing effusion (dashed lines), visceral (vpt), and parietal (ppt) pleural tumors on the costophrenic angle (ca), the diaphragm (d), and the chest wall (cw, arrows). t, tumors; l, lungs; h, heart.DMay–Gruenwald–Giemsa‐stained pleural cells (macrophages, MΦ: black arrow; lymphocytes, LΦ: purple arrow; neutrophils, NΦ: green arrow; eosinophils, EΦ: orange arrow).EEffusion cytology and biochemistry data summary (total *n* = 10 mice; *n* = 4, 3, and 3 effusions from mice injected with KPM1, KPM2, and KPM3 cells, respectively, were analyzed and shown are pooled data).F, G
*C57BL*/*6* mice received pleural KPM1 cells followed by a single intrapleural injection of liposomes containing 1% DMSO or 15 mg/kg deltarasin in 1% DMSO at day 9 post‐tumor cells. Shown are data summaries of MPE volume (*n* = 8 and 7 DMSO and deltarasin‐treated mice/group, respectively) and pleural fluid nucleated cells at day 19 post‐KPM1 cells (F), as well as representative images of pleural effusions (dashed lines) and tumors (t in [G]). Data information: In (A), data are presented as Kaplan–Meier survival estimates (lines), 95% confidence interval (shaded areas), and number of mice at risk (*n*). *P*, probability of overall comparison and of any comparison to PMC, log‐rank test. In (B) and (F), data are presented as raw data points (circles), rotated kernel density distribution (violins), and medians (lines). Numbers in red font and arrows in (F) indicate end‐point reduction by deltarasin effect. *P*, probability, one‐way ANOVA (B) or Student's *t*‐test (F). *, **, ***, and ****: *P* < 0.05, *P* < 0.01, *P* < 0.001, and *P* < 0.0001, respectively, for comparison with PMC, Bonferroni post‐tests. In (E), data are presented as mean ± 95% confidence interval. LDH, lactate dehydrogenase. Source data are available online for this figure.

We subsequently isolated three different MPM cell lines from Ad‐*Cre*‐treated *KRAS* ^G12D^ *;Trp53f*/*f* mice (called KPM1–3) using long‐term tumor culture (Pauli *et al*, 2017; Kanellakis *et al*, 2019, 2020). KPM cells displayed anchorage‐independent growth (anoikis), spindle‐shaped morphology, and rapid growth in minimal‐supplemented media and in soft agar. In addition, KPM cells were tumorigenic when injected subcutaneously into the flank of *C57BL*/*6* mice and carried the original *KRAS* ^G12D^/*Trp53* lesions (Fig 7A–E, and Appendix Fig S1). KPM cells and their parental tumors featured enhanced *Bap1* and *Cdkn2a*, but not *Nf2* expression (Fig 7E and F, and Appendix Fig S1), consistent with previous work that identified *TP53*‐mediated repression of *BRCA1* and *CDKN2A* expression (Stott *et al*, 1998; Arizti *et al*, 2000). RNA sequencing of KPM cells (GEO dataset GSE94415; Data ref: Stathopoulos *et al*, 2017) revealed that they carry the pathogenic *KRAS* ^G12D^/*Trp53* lesions, but also multiple stochastic single nucleotide variants in exon 6 and insertions in exon 11 of *Bap1*, all validated by Sanger sequencing, although immunohistochemistry revealed persistent nuclear BAP1 expression rendering these Bap1 mutations of uncertain functional significance (Nasu *et al*, 2015) (Fig EV5). Finally, 2 × 10^5^ pleural‐delivered KPM cells could inflict to naïve *C57BL*/*6* mice secondary disease identical to primary MPM of *KRAS* ^G12D^ *;Trp53f*/*f* mice in terms of manifestation, pathology, cytology, and biochemistry (Fig 8A–E), fulfilling modified Koch's postulates (Byrd & Segre, 2016).

To determine the potential efficacy of KRAS inhibition against murine KRAS/TP53‐driven MPM, *C57BL*/*6* mice received pleural KPM1 cells, followed by a single intrapleural injection of liposomal‐encapsulated KRAS inhibitor deltarasin (15 mg/kg; Zimmermann *et al*, 2013) or empty liposomes on day nine post‐tumor cells, in order to allow initial tumor implantation in the pleural space (Agalioti *et al*, 2017). At day 19 after pleural injection of KPM1 cells, deltarasin‐treated *C57BL*/*6* mice developed fewer and smaller MPE with decreased cellularity compared with controls (Fig 8F and G). These results collectively show that our murine MPM is indeed malignant, originate from recombined mesothelial cells, and cause transplantable disease that can be used for hypothesis and drug testing.

**Figure 9:**
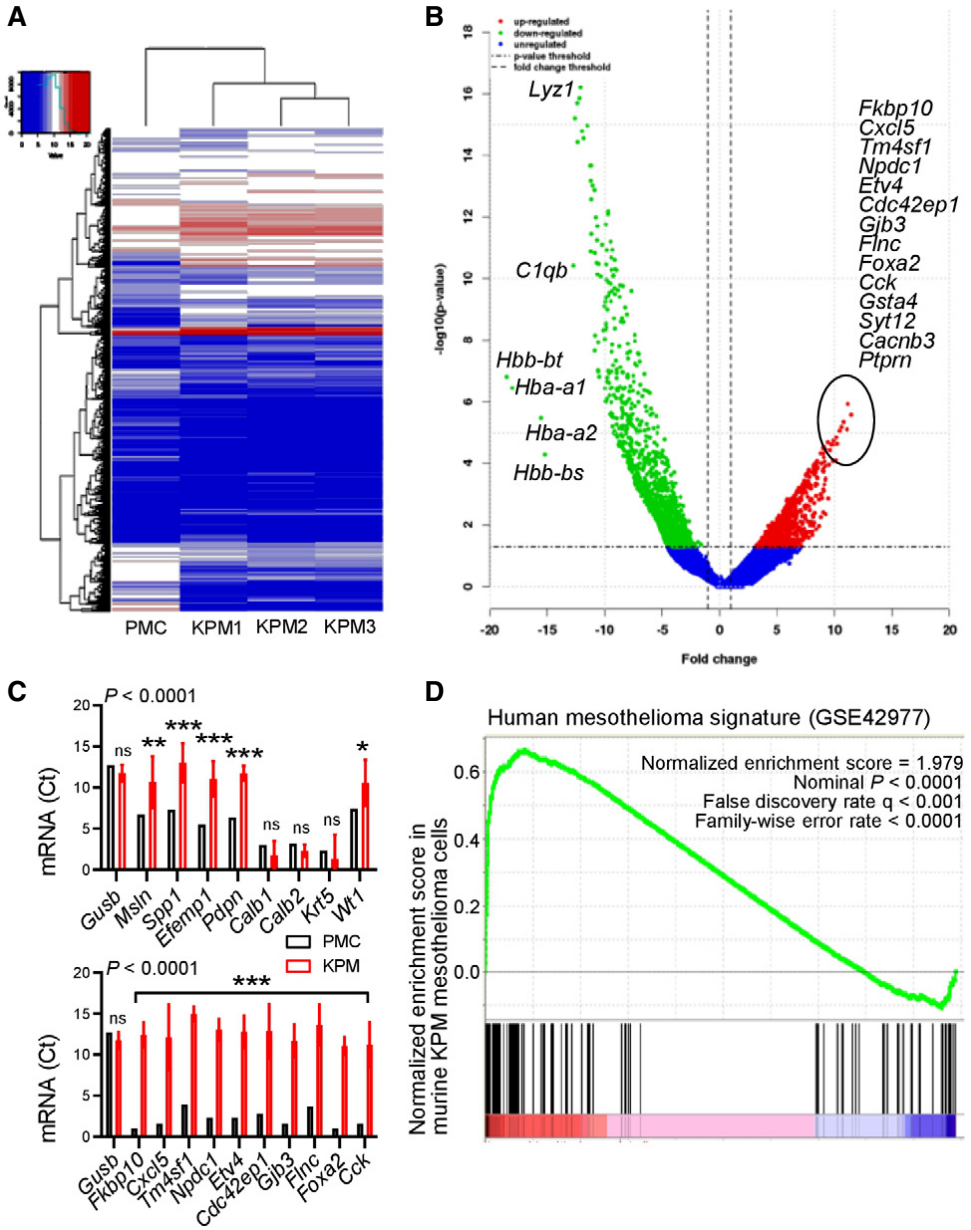
The molecular signature of KPM cells is enriched in human mesothelioma RNA sequencing results (GEO dataset GSE94415) of *KRAS* ^G12D^ *;Trp53f*/*f* mesothelioma (KPM) cells (*n* = 3) compared with pleural mesothelial cells (PMC; *n* = 1 pooled triplicate). *n* denotes biological replicates, since pooled triplicate technical replicates from each cell line were sequenced.
Unsupervised hierarchical clustering shows distinctive gene expression of KPM versus PMC.Volcano plot showing some top KPM versus PMC differentially expressed genes.KPM and PMC expression of classic mesothelioma markers (top) and top KPM versus PMC overexpressed genes (bottom).Gene set enrichment analysis, including enrichment score and nominal probability value of the 150 gene‐signature specifically over‐represented in human mesothelioma compared with other thoracic malignancies derived from 113 patients (GSE42977) within the transcriptome of KPM cells versus PMC shows significant enrichment of the human mesothelioma signature in KPM cells. Data information: In (C), data are presented as mean (columns) and 95% confidence interval (bars). *P*: probability, two‐way ANOVA. ns, *, **, and ***: *P* > 0.05, *P* < 0.05, *P* < 0.01, and *P* < 0.001, respectively, compared with PMC, Bonferroni post‐tests. Source data are available online for this figure.

Finally, RNA sequencing of KPM cells comparative to normal pleural mesothelial cells revealed a distinctive transcriptomic signature that included classic mesothelioma markers (*Msln, Spp1, Efemp1, Pdpn, Wt1*) as well as new candidate mesothelioma genes (Fig 9A–C and Appendix Table S4). A human 150–gene mesothelioma signature derived from a cohort of 113 patients via comparison of MPM against multiple other malignancies (GSE42977; De Rienzo *et al*, 2013; Data ref: De Rienzo *et al*, 2012) was highly enriched in our KPM cell line signature (Fig 9D). These data indicate that murine *KRAS*/*TP53*‐driven MPM present *Bap1* mutations, a gene expression profile that is highly similar to human MPM, and can be used for transplantable and druggable MPM models in syngeneic mice. Collectively, the murine and human findings support the existence of a *KRAS*‐driven subset of MPM patients or clones that are likely missed during sequencing and/or sampling (Comertpay *et al*, 2014; Li *et al*, 2020).

## Discussion

Our results demonstrate that, alone or in combination with *TP53*, *KRAS* is perturbed in a proportion of human MPM and can potentially drive the murine mesothelium toward MPM development. *KRAS* mutations, amplifications, and overexpression, as well as chromosome 12p gains, are shown to exist in 20% of patients from the TCGA MPM dataset and low allelic frequency *KRAS* mutations are discovered in 50% of MPM samples from our own human cohorts using sensitive techniques. Furthermore, *KRAS* mutations are shown to occasionally co‐exist with *TP53* mutations, to repulse *NF2* mutations, and to be associated with biphasic MPM histology. Targeting of oncogenic *KRAS* ^G12D^ alone to the pleural mesothelium caused epithelioid MPM in mice and together with *Trp53* deletion resulted in biphasic MPM with MPE. We further show that murine MPM carry the initiating *KRAS* ^G12D^/*Trp53* mutations and multiple secondary *Bap1* mutations, are transplantable and druggable, and highly similar to human MPM in terms of molecular markers and gene expression. Collectively, the data support a pathogenic role for *KRAS* mutations in a fraction of MPMs and provide new models to study this patient group.

Our striking findings can be reconciled with the sporadic nature of *KRAS* mutations in human MPM sequencing studies (Bott *et al*, 2011; Guo *et al*, 2015; Bueno *et al*, 2016; Hmeljak *et al*, 2018) and the incomplete penetrance of standalone *Bap1*, *Cdkn2a*, *Nf2*, or *Trp53* deletions in causing MPM in mice (Jongsma *et al*, 2008; Guo *et al*, 2014; Menges *et al*, 2014; Xu *et al*, 2014; Kukuyan *et al*, 2019). To this end, mesothelial *KRAS* mutations may initiate MPM in some patients, but may be lost during sampling and sequencing, as has been shown for other mutations in LUAD that persist at a subclonal level (Abbosh *et al*, 2017; Jamal‐Hanjani *et al*, 2017). The low allelic frequency of *KRAS* mutations is explicable by their heterozygous nature and the robust inflammatory responses *KRAS*‐mutant tumors generate (Agalioti *et al*, 2017; Marazioti *et al*, 2018) and is not limiting their driver capabilities in other tumor types (Abbosh *et al*, 2017; Jamal‐Hanjani *et al*, 2017; Li *et al*, 2020). The fact that these mutations were not detected by most next‐generation sequencing studies of MPM can be explained by the relative low sensitivity of these methods compared with ddPCR, as well as the low allelic frequency of KRAS mutations. To this end, typical read depths of 50–100 are employed in most next‐generation sequencing studies yielding a sensitivity of 1–2%, compared with the theoretical 0.005% or actual 0.1% of ddPCR (Demuth *et al*, 2018). In addition, most next‐generation sequencing studies set discovery cutoffs of 25% allelic frequency, likely rendering many *KRAS* mutations undiscovered. Our findings are plausible, since MPM is likely polyclonal (Comertpay *et al*, 2014), cell lines display *KRAS* activation and mutations (Patel *et al*, 2007; Agalioti *et al*, 2017), *NF2* is a *KRAS* suppressor (Tikoo *et al*, 1994), and *KRAS* signaling is interconnected with the *TP53* cell cycle checkpoint (Matallanas *et al*, 2011). The postulation that KRAS mutations in MPM might be early events can be tested in the future by genome doubling analyses. Taken together, our data and the literature support that, in a subset of patients, low allelic frequency *KRAS* alterations conditionally accomplice with *TP53* to drive mesothelial cells toward MPM. These tumors may be selectively responsive to *KRAS* blockade and detectable by sensitive methods like ddPCR or maximal depth sequencing (Li *et al*, 2020).

We also corroborate the critical role of *TP53* in MPM progression, since *TP53* mutations are frequent in MPM. Although standalone T*rp53* deletion did not induce MPM in mice, it promoted *KRAS* ^G12D^‐driven MPM progression and biphasic histology, as was also observed in combination with *Nf2* and *Tsc1* deletion (Jongsma *et al*, 2008; Guo *et al*, 2014), suggesting that *Trp53* loss may conditionally cooperate with other oncogenes in MPM. In addition, *Trp53‐*deleted *KRAS* ^G12D^ MPM was accompanied by effusions, a human MPM phenotype that likely affects survival (Ryu *et al*, 2014) and that was previously not reproducible in mice. Again, *Trp53* loss was not causative, but likely potentiated the effusion‐promoting effects of *KRAS*, which we recently identified in metastatic effusions (Agalioti *et al*, 2017). Taken together with published work, our findings functionally validate the role of *TP53* mutations in human MPM in driving biphasic histology, tumor progression and metastasis, and poor survival (Bueno *et al*, 2016; Yap *et al*, 2017). Hence, TP53‐targeted therapies may be prioritized for biphasic MPM when available (Brown *et al*, 2009).

Another surprising finding was the multiple and different *Bap1* mutations of our MPM cell lines, since they originated from tumors inflicted by *KRAS* ^G12D^ and *Trp53* loss. Frequent copy number loss and recurrent somatic mutations in *BAP1* have been identified in MPM (Bott *et al*, 2011; Guo *et al*, 2015; Nasu *et al*, 2015). Based on the multiplicity and variety of the *Bap1* mutations we observed, we postulate that they were secondarily triggered by the genomic instability caused from combined *KRAS* mutation and *TP53* loss. Whatever their cause may be, their presence strengthens our findings of an involvement of KRAS signaling in MPM pathobiology, as well as the relevance of the novel mouse models we developed, since *Bap1* is the single most commonly mutated gene in human MPM.

Research on MPM is hampered by the paucity of mouse models (Blanquart *et al*, 2020). We provide multiple new mouse models with defined phenotype, histology, and latency: (i) a genetic mouse model of pleural epithelioid MPM; (ii) genetic and transplantable models of pleural and peritoneal biphasic MPM with accompanying effusion; and (iii) three new MPM cell lines of defined genotype, transcriptome, and phenotype that are syngeneic to *C57BL*/*6* mice. These are positioned to enhance MPM research by overcoming the need for immune compromise providing intact immune responses critical for MPM pathogenesis (Burt *et al*, 2012; Westbom *et al*, 2014; Kadariya *et al*, 2016; Patil *et al*, 2018), by widening the repertoire of existing cell lines, by recapitulating MPM with effusion, and by addressing pleural MPM.

In conclusion, our findings support that oncogenic *KRAS* signaling causes MPM in a proportion of humans and in mice. As some mutations along this signaling pathway are currently druggable or are likely to become such in the near future (Herbst *et al*, 2002; Brown *et al*, 2009; Flaherty *et al*, 2010; Stephen *et al*, 2014), our findings may facilitate therapeutic innovation. Pending validation of our human findings in larger cohorts, we provide novel tools for the study of a molecular subclass of MPM that will hopefully aid in drug discovery and personalized treatment of patients with MPM driven by KRAS signaling.

## Materials and Methods

### Computational biologic analyses

The dataset for Fig 1A was generated by manual curation of COSMIC data (https://cancer.sanger.ac.uk/cosmic/browse/tissue?wgs=off&sn=pleura&ss=all&hn=mesothelioma&sh=&in=t&src=tissue&all_data=n). The dataset for Fig 1B was generated by manual curation of the main text and supplementary data of publications (Bott *et al*, 2011; Enomoto *et al*, 2012; Mezzapelle *et al*, 2013; Shukuya *et al*; 2014; Guo *et al*, 2015; Lo Iacono *et al*, 2015; Bueno *et al*, 2016; De Rienzo *et al*, 2016; Kato *et al*, 2016; Hmeljak *et al*, 2018). Raw data from 86 human TCGA MPM patients were retrieved from the cBioPortal for Cancer Genomics (www.cbioportal.org/) using inputs “mesothelioma”, “Mesothelioma (TCGA, PanCancer Atlas)”, “Query by Gene *KRAS* and *TP53*”, “Mutations”, “Putative copy‐number alterations from GISTIC”, “mRNA expression *z*‐scores”, and “Protein expression *z*‐scores” were downloaded and analyzed. Gene expression data from these patients, normalized with the log_2_(fpkm‐uq + 1) method, were downloaded (https://xenabrowser.net/datapages/?dataset=TCGA‐MESO.htseq_fpkm‐uq.tsv&host=https%3A%2F%2Fgdc.xenahubs.net&removeHub=https%3A%2F%2Fxena.treehouse.gi.ucsc.edu%3A443), ENSEMBL gene IDs were converted to gene symbols using https://www.biotools.fr/mouse/ensembl_symbol_converter, the data were filtered, differential gene expression (*Δ*GE) was analyzed, and heatmap visualization was performed using R* and packages limma R version 3.42.2 (https://bioconductor.org/packages/release/bioc/html/limma.html) and edgeR (https://bioconductor.org/packages/release/bioc/html/edgeR.html). Both rows and columns were clustered using Pearson correlation and complete linkage. All mutations (*n* = 2,150) of all patients (*n* = 86) with MPM from the TCGA pan‐cancer dataset were retrieved from www.cbioportal.org/ and were fed into the protein analysis through evolutionary relationships (PANTHER) Classification System (www.pantherdb.org/) using parameters: organism, *Homo Sapiens*; analysis, statistical overrepresentation test > PANTHER pathways or reactome pathways (both analyses were done); whole‐genome reference list: *Homo Sapiens*; test type: binomial; and correction: false discovery rate. All raw data from the two independent PANTHER and reactome pathway analyses were retrieved, merged, and analyzed. Gene set enrichment analysis (GSEA) was performed with the Broad Institute pre‐ranked GSEA module software (http://software.broadinstitute.org/gsea/index.jsp;Subramanian *et al*, 2005). All aforementioned raw data were downloaded from the sources referenced above in *.csv format, are provided as source data files with this publication, and were reanalyzed using R*, Prism v8.0 (GraphPad, La Jolla, CA), and Excel (Microsoft, Redmont, WA).

### Reagents

Adenoviruses type 5 (Ad) encoding *Melanotus* luciferase (*Luc*) or CRE‐recombinase (*Cre*) were from the Vector Development Laboratory, Baylor College of Medicine (Houston, TX); 3‐(4,5‐dimethylthiazol‐2‐yl)‐2,5‐diphenyltetrazolium bromide (MTT) assay from Sigma‐Aldrich (St. Louis, MO), and D‐luciferin from Gold Biotechnology (St. Louis, MO). Primers and antibodies are listed in Appendix Tables S5 and S6. All cell culture reagents were from Thermo Fisher Scientific.

### Human studies

All human experiments conformed to the principles set out in the WMA Declaration of Helsinki and the Department of Health and Human Services Belmont Report. The Munich clinical study was prospectively approved by the Ludwig‐Maximilians‐University Munich Ethics Committee (approvals #623–15 and #711–16). All patients gave written informed consent *a priori*. Diagnoses were made according to current standards by a board‐certified pathologist at the Asklepios Fachkliniken Gauting, Munich, Germany. Pleural fluid was centrifuged at 300 *g* for 10 min at 4°C, genomic DNA was extracted from cell pellets, supernatants, and pleural tumor tissues using TRIzol (Thermo Fisher) and purified using GenElute Mammalian Genomic DNA Miniprep (Sigma Aldrich), and 200 ng DNA were used to analyze *KRAS* codons 12/13 and 61, and *TP53* copies with ddPCR *KRAS* G12/G13, *KRAS* G61, *TP53* CNV, and TERT CNV Kits and QuantaSoft Analysis Pro software (BioRad, Hercules, CA) as described elsewhere (Poole *et al*, 2019). Thresholds for *KRAS* ^WT^, *KRAS* ^MUT^, *TP53*, and *TERT* droplet amplitude gates were, respectively, 6,000, 10,000, 5,500, and 7,000. Data were normalized by accepted droplet numbers to yield absolute mutant (^MUT^) and wild‐type (^WT^) droplet percentages, which were determined using thresholds derived from cell line controls and from LUAD patient samples clinically confirmed to have *KRAS* mutations and *TP53* copy number changes, according to the formula:

$$\begin{array}{ll} & KRAS\;\text{mutant}\;\text{copies}\;\%=\\ & \frac{n_{\text{positive mutant droplets}}}{\left(n_{\text{positive mutant droplets}}+n_{\text{positive wild type droplets}}\right)}*100 \end{array}$$

$$TP53\;\text{copies}\;\%=\frac{n_{\text{TP53 positive droplets}}}{n_{\text{TERT positive droplets}}}*100.$$

In the Nantes Study, MPM cell lines, as well as pleural fluid cells and supernatants, were derived from pleural fluid aspirates obtained for diagnostic and therapeutic purposes. The study was approved by the French Ministry of Research (DC‐2011‐1399), and all patients gave written informed consent *a priori* for their excess pleural fluid to be used for the establishment of cell lines. MPE samples from over 120 patients with MPM were used to generate the 33 cell lines, since the success rate is < 30%, as described elsewhere (Gueugnon *et al*, 2011; Delaunay *et al*, 2020). Diagnoses were established by both fluid cytology and immunohistochemical staining of pleural biopsies performed by the pathology department at Laënnec Hospital (St‐Herblain, France) and then externally confirmed by MESOPATH, the French panel of pathology experts for the diagnosis of mesothelioma. All recruited patients had received no prior anticancer therapy. All cell lines were maintained in RPMI‐1640 medium supplemented with 2 mM l‐glutamine, 100 IU/ml penicillin, 0.1 mg/ml streptomycin, and 10% heat‐inactivated fetal calf serum and cultured at 37°C in 5% CO_2_‐95% air. Genomic DNA from 33 MPM cell lines was extracted with Nucleospin Blood kit (Macherey‐Nagel, Düren, Germany) and 500 ng were hybridized to Affymetrix CytoScanHD Arrays (Thermo Fisher). Detection, quantification, and visualization of single nucleotide variations (SNV) and copy number alterations (CNA) were performed using Affymetrix Chromosome Analysis Suite v3.1.1.27 (Thermo Fisher) and data are available at GEO datasets (GSE134349; Data ref: Blanquart *et al*, 2019). The cell lines were also sequenced in a targeted fashion focusing on 21 genes and the TERT promoter on a MiSeq system (Illumina, San Diego, CA) (Quetel *et al*, 2020). The MAPED (Clinical identification of malignant pleural effusions in the emergency department) study entailed a few samples from patients enrolled in a prospective clinical trial (preprint: Marazioti *et al*, 2021). MAPED was registered with ClinicalTrials.gov (#NCT03319472), and written informed consent was obtained from all patients *a priori*. MAPED was approved by the University of Patras Ethics Committee (approval #22699/21.11.2013). Pleural fluid was centrifuged at 300 *g* for 10 min at 4°C, RNA and DNA were extracted from cell pellets using TRIzol (Thermo Fisher) and purified using GenElute Mammalian Genomic DNA Miniprep (Sigma‐Aldrich), and 200 ng RNA/DNA were used for RT–PCR, qPCR, and Sanger sequencing. The Istanbul study was approved by the Koç University Ethics Committee on Human Research (approval #2021.223.IRB2.042/06.05.2021). Both Nantes pleural fluid and Istanbul pleural tumor specimens were processed and analyzed identical to the Munich study.

### Mice

*C57BL*/*6* (#000664), *B6.129(Cg)‐Gt(ROSA)26Sor^tm4(ACTB‐tdTomato,‐EGFP)Luo^*/*J* (*mT*/*mG*; #007676; Muzumdar *et al*, 2007), *FVB‐Tg(CAG‐luc,‐GFP)L2G85Chco*/*J* (*CAG.Luc.eGFP*; #008450; Cao *et al*, 2004)^64^, B6.129S4‐*Kras^tm4Tyj^*/*J* (*KRAS* ^G12D^; #008179; Jackson *et al*, 2001), and B6.129P2‐*Trp53^tm1Brn^*/*J* (*Trp53f*/*f*; #008462; Meylan *et al*, 2009) mice were obtained from Jackson Laboratories (Bar Harbor, ME) and bred on the *C57BL*/*6* background at the University of Patras Center for Animal Models of Disease. Experiments were approved by the Prefecture of Western Greece's Veterinary Administration (approval 118018/578‐30.04.2014) and were conducted according to Directive 2010/63/EU (http://eur‐lex.europa.eu/legal‐content/EN/TXT/?uri=CELEX%3A32010L0063). Sex‐, weight (20–25 g)‐, and age (6–12 week)‐matched experimental mice were used, and their numbers (total *n* = 432) are detailed in Appendix Table S7.

### Mesothelial transgene delivery

Isoflurane‐anesthetized *C57BL*/*6* and *mT*/*mG* mice received 5 × 10^8^ PFU intrapleural or intraperitoneal Ad‐*Cre* or Ad‐*Luc* in 100 μl PBS and were serially imaged for bioluminescence on a Xenogen Lumina II (Perkin‐Elmer, Waltham, MA) after receiving 1 mg retro‐orbital D‐luciferin under isoflurane anesthesia, and data were analyzed using Living Image v.4.2 (Perkin‐Elmer; Stathopoulos *et al*, 2006; Spella *et al*, 2019), or were euthanized and pleural lavage was performed, lungs were explanted, and parietal pleura was stripped. For pleural lavage, 1 ml PBS was injected, was withdrawn after 30 s, and was cytocentrifuged onto glass slides (5 × 10^4^ cells, 300 *g*, 10 min) using CellSpin (Tharmac, Marburg, Germany). Lungs were embedded in optimal cutting temperature (OCT; Sakura, Tokyo, Japan) and sectioned into 10‐µm cryosections. The parietal pleura was placed apical side up onto glass slides. Samples were stained with Hoechst 55238 and were examined on AxioObserver D1 (Zeiss, Jena, Germany) or TCS SP5 (Leica, Heidelberg, Germany) microscopes.

### Primary MPM models

Wild‐type (*Wt*), *KRAS* ^G12D^, and *Trp53f*/*f* mice were intercrossed and all possible offspring genotypes received isoflurane anesthesia and 5 × 10^8^ PFU intrapleural or intraperitoneal Ad‐*Cre*. Mice were monitored daily and sacrificed when moribund or prematurely for pathology. Mice with pleural fluid volume ≥ 100 μl were judged to have effusions that were aspirated. Animals with pleural fluid volume < 100 μl were judged not to have effusions and underwent pleural lavage. For isolation of primary murine pleural mesothelial cells (PMC), pleural myeloid and lymphoid cells were removed by pleural lavage followed by pleural instillation of 1 ml DMEM, 2% trypsin EDTA, aspiration after 1 min, and culture.

### Bone marrow transfer

For adoptive BMT, *C57BL*/*6* mice received 10^7^ bone marrow cells obtained from *CAG.Luc.eGFP* donors i.v. 12 h after total‐body irradiation (1,100 Rad). Full bone marrow reconstitution was completed after one month, as described elsewhere (Agalioti *et al*, 2017).

### Transplantable mesothelioma cell lines

Murine *KRAS* ^G12D^ *;Trp53f*/*f* pleural mesotheliomas were minced and cultured in DMEM 10% FBS for > 30 passages, yielding three *KRAS* ^G12D^ *;Trp53f*/*f* mesothelioma (KPM1–3) cell lines, which were compared to AE17 cells (*Kras^G12C^*‐mutant asbestos‐induced murine mesothelioma) and PMC (Agalioti *et al*, 2017). PMC were generated in our laboratory as primary cultures of murine pleural lavage with DMEM 2% trypsin, whereas AE17 cells were donated by Dr. YC Gary Lee (University of Western Australia, Perth, Australia) and have been both extensively described elsewhere (Giannou *et al*, 2015, 2017; Agalioti *et al*, 2017; Marazioti *et al*, 2018). For this, 2 × 10^5^ cells in 100 μl PBS were delivered intrapleurally to isoflurane‐anesthetized *C57BL*/*6* mice that were followed as above. For solid tumor formation, *C57BL*/*6* mice received 10^6^ subcutaneous PMC, KPM, or AE17 cells in the rear flank, three vertical tumor dimensions (δ^1^, δ^2^, δ^3^) were monitored serially, and the formula πδ^1^δ^2^δ^3^/6 was used to calculate tumor volume. RNA sequencing was done on an IonTorrent sequencer (Thermo Fisher); data were deposited at GEO datasets (GSE94415) and were analyzed using Bioconductor (Data ref: Stathopoulos *et al*, 2017). Gene set enrichment was done with the Broad Institute pre‐ranked GSEA module (Subramanian *et al*, 2005).

### PCR and Sanger sequencing

Cellular RNA was isolated using TRIzol (Thermo Fisher Scientific, Waltham, MA) followed by RNAeasy purification and genomic DNA removal (Qiagen, Hilden, Germany). For tumor RNA, tissues were passed through 70‐μm strainers (BD Biosciences, San Jose, CA) and 10^7^ cells were subjected to RNA extraction. One μg RNA was reverse‐transcribed using Oligo(dT)_18_ and Superscript III (Thermo Fisher). cDNAs were amplified using specific primers (Appendix Table S5) and Phusion Hot Start Flex polymerase (New England Biolabs, Ipswich, MA). DNA fragments were run on 2% agarose gels or were purified with NucleoSpin gel and PCR clean‐up columns (Macherey‐Nagel, Düren, Germany) and were sequenced using their primers by VBC Biotech (Vienna, Austria). qPCR was performed using specific primers (Appendix Table S5) and SYBR FAST qPCR Kit (Kapa Biosystems, Wilmington, MA) in a StepOne cycler (Applied Biosystems, Carlsbad, CA). Ct values from triplicate reactions were analyzed with the $2^{‐\Delta \text{CT}}$ method (Pfaffl, 2001). mRNA abundance was determined relative to glycuronidase beta (*Gusb*) and is given as $2^{‐\Delta \text{CT}}=2^{‐(\text{Ct}\;\text{of}\;\text{transcript})‐(\text{Ct}\;\text{of}\;\text{Gusb})}$. The Sanger sequencing trace files were further analyzed for double peak parser using Bioconductor (https://www.bioconductor.org/) with a threshold of 25 Phred quality core (Ewing *et al*, 1998). The mismatch basecalls in respect to the wild‐type samples were grouped by sample and used as template to generate the lollipop plot per each KPM cell line for a visual representation of all the mutations detected (Jay & Brouwer, 2016). Lollipop plots were generated using MutationMapper (https://www.cbioportal.org/mutation_mapper; Cerami *et al*, 2012).

### RNA sequencing

RNA sequencing was done on an IonTorrent sequencer (Thermo Fisher), and data were analyzed using Bioconductor (https://www.bioconductor.org/). File alignments were performed with Τmap (https://github.com/iontorrent/TMAP). Coverage and alignments plot from sequencing were generated using Integrative genome viewer (Robinson *et al*, 2011). Alignments are represented as gray polygons with reads mismatching the reference indicated by color. Loci with a large percentage of mismatches relative to the reference are flagged in the coverage plot as color‐coded bars. Alignments with inferred small insertion or small deletion are represented with vertical or horizontal bars, respectively. Gene set enrichment analysis (GSEA) was performed with the Broad Institute pre‐ranked GSEA module software (http://software.broadinstitute.org/gsea/index.jsp; Subramanian *et al*, 2005). The raw *.bam files, one for each RNA‐Seq sample, were summarized to a gene read counts table, using the Bioconductor package GenomicRanges. In the final read counts table, each row represented one gene, each column one RNAseq sample, and each cell the corresponding read counts associated with each row and column. The gene counts table was normalized for inherent systematic or experimental biases (e.g., sequencing depth, gene length, and GC content bias) using the Bioconductor package DESeq after removing genes that had zero counts over all RNASeq samples (20,007 genes). The output of the normalization algorithm was a table with normalized counts, which can be used for differential expression analysis with statistical algorithms developed specifically for count data. Prior to the statistical testing procedure, the gene read counts were filtered for possible artifacts that could affect the subsequent statistical testing procedures. Genes presenting any of the following were excluded from further analysis: (i) genes with length less than 500 bp (2,051 genes), (ii) genes whose average reads per 100 bp was less than the 25^th^ percentile of the total normalized distribution of average reads per 100 bp (0 genes with cutoff value 0.02248 average reads per 100 bp), (iii) genes with read counts below the median read counts of the total normalized count distribution (11,358 genes with cutoff value 16 normalized read counts). The total number of genes excluded due to the application of gene filters was 5,298. The total (unified) number of genes excluded due to the application of all filters was 32,595. The resulting gene counts table was subjected to differential expression analysis for the contrast KPM versus PMC using the Bioconductor package DESeq. The final numbers of statistically significant differentially expressed genes were 2,344 genes and of these, 650 were up‐regulated and 1,694 were down‐regulated according to an absolute fold‐change cutoff value of 2.

### Cell culture

All KPM cell lines are available upon request. Cells were cultured at 37°C in 5% CO_2_‐95% air using DMEM 10% FBS, 2 mM l‐glutamine, 1 mM pyruvate, 100 U/ml penicillin, and 100 mg/ml streptomycin and were tested biannually for identity (by short tandem repeats) and *Mycoplasma Spp*. (by PCR). *In vitro* cell proliferation was determined using 3‐(4,5‐dimethylthiazol‐2‐yl)‐2,5‐diphenyltetrazolium bromide (MTT) assay. For *in vivo* injections, cells were harvested with trypsin, incubated with Trypan blue, counted on a hemocytometer, and > 95% viable cells were injected into the pleural space (2 × 10^5^) or into the skin (10^6^) as described elsewhere (Agalioti *et al*, 2017). Mouse numbers used are detailed in Appendix Table S7.

### Cell and tissue analyses

MPE fluid was diluted in 10‐fold excess red blood cells lysis buffer (155 mM NH_4_Cl, 12 mM NaHCO_3_, 0.1 mM EDTA). Total pleural cell counts were determined microscopically in a hemocytometer and cytocentrifugal specimens (5 × 10^4^ cells each) of pleural fluid cells were fixed with methanol for 2 min. Cells were stained with May–Grünwald stain in 1 mM Na_2_HPO_4_, 2.5 mM KH_2_PO_4_, pH = 6.4 for 6 min and Giemsa stain in 2 mM Na_2_HPO_4_, 5 mM KH_2_PO_4_, pH = 6.4 for 40 min, washed with H_2_O, and dried. Slides were mounted with Entellan (Merck Millipore, Darmstadt, Germany), coverslipped, and analyzed. For flow cytometry, 10^6^ nucleated pleural fluid cells suspended in 50 μl PBS supplemented with 2% FBS and 0.1% NaN_3_ were stained with the indicated antibodies according to manufacturer's instructions (Appendix Table S6) for 20 min in the dark, washed, and resuspended in buffer for further analysis. Lungs, visceral pleural tumors, parietal pleural tumors, and chest walls were fixed in 4% paraformaldehyde overnight, embedded in paraffin or optimal cutting temperature (OCT) and were stored at room temperature or −80°C, respectively. Five‐μm paraffin or 10‐μm cryosections were mounted on glass slides. Sections were labeled using the indicated antibodies (Appendix Table S6), counterstained with Envision (Dako, Carpinteria, CA) or Hoechst 33258 (Sigma‐Aldrich, St. Louis, MO), and mounted with Entellan new (Merck Millipore) or Mowiol 4‐88 (Calbiochem, Gibbstown, NJ). For isotype control, primary antibody was omitted. Bright‐field and fluorescent microscopy were done on AxioLab.A1 (Zeiss), AxioObserver.D1 (Zeiss), or TCS SP5 (Leica) microscopes and digital images were processed with Fiji (Schindelin *et al*, 2012).

### Liposomal deltarasin preparation and treatment

Deltarasin‐encapsulating liposomes were prepared as described elsewhere (Markoutsa *et al*, 2014; Marazioti *et al*, 2019), by freeze‐drying 30 mg of empty DSPC/PG/Chol (9:1:5 mol/mol/mol) unilamelar sonicated vesicles with 1 ml of deltarasin solution (5 mg/ml) in PBS, or plain PBS (for empty liposomes), followed by controlled rehydration. Liposome size was decreased by extrusion though Lipo‐so‐fast extruder polycarbonate membranes (Avestin Europe, Mannheim, Germany) with 400‐nm pore diameter. Liposome lipid concentration, size distribution, surface charge (zeta‐sizer, Malvern Panalytical Ltd, Malvern, United Kingdom), and drug encapsulation efficiency were estimated by measuring non‐liposomal drug absorption at 284 nm as reported elsewhere (Markoutsa *et al*, 2014; Marazioti *et al*, 2019). Deltarasin‐encapsulating liposomes were delivered intrapleurally into *C57BL*/*6* mice 9 days post‐intrapleural KPM1 cells, when the first pleural tumors were already established (Agalioti *et al*, 2017).

### Statistics

Sample size was estimated using G*power (Faul *et al*, 2007) assuming *α* = 0.05, *β* = 0.05, and effect size *d* or *φ* = 1.5. Animals were allocated to treatments by alternation and transgenic animals case‐control‐wise. Data acquisition was blinded and no data were excluded from analyses. Data were tested for normality of distribution by Kolmogorov–Smirnov test and are given as mean ± 95% confidence interval (CI). Sample size (*n*) refers to biological replicates. Differences in means or medians were examined by *t*‐test, Mann–Whitney test, Wilcoxon matched‐pairs signed rank test, one‐way analysis of variance (ANOVA) with Tukey's or Bonferroni's post‐tests, or Kruskal–Wallis test with Dunn's post‐tests, as indicated and appropriate. Differences in frequencies were tested by Fischer's exact or *χ* ^2^ tests. Molecular and longitudinal (bioluminescence, MTT, tumor growth) data were analyzed by two‐way ANOVA with Bonferroni's, Sidak's, Dunnett's, or Tukey's post‐tests, or with two‐stage linear step‐up procedure of Benjamini, Krieger, and Yekutieli. Survival was analyzed using Kaplan–Meier estimates, log‐rank (Mantel–Cox) test for probability, and Mantel–Haenszel estimates of hazard ratio. Probability (*P*) values are two‐tailed and *P* < 0.05 was considered significant. Analyses and plots were done on Prism v8.0 (GraphPad, La Jolla, CA) and Excel (Microsoft, Redmont, WA).

## Author contributions

AM, ACK, GAG, SJB, and GN designed and carried out experiments, analyzed data, provided critical intellectual input, and generated portions of the paper draft; CB, DJ, SD, and MG designed and carried out microarray analyses, provided the French MPM cell line cohort, and provided and characterized the Nantes patient cohort; HB, ÖK, DM, ŞD, ST, SE, ÖY, PB, and PF provided and characterized the Istanbul patient cohort; SAIW, LT, MAAP, and CMH designed and carried out sequencing experiments and analysis, immunohistochemistry, RNA sequencing analysis, and digital droplet PCR; LVK, IK, ML, RAH, and JB provided the German MPM and LUAD tumor cohort; MI and MV performed *in vivo* CRE reporter assays and experiments using *KRAS* ^G12D^ mice; ACK and IL performed molecular phenotyping of murine tumors; DEW performed GSEA; HP evaluated and diagnosed mouse pathology; SGA prepared liposomes; IP, MS, and IG designed and performed experiments and provided critical intellectual input and partial funding; A‐SL carried out and analyzed immunohistochemistry and digital droplet PCR, and organized the experiments for the revision of the manuscript; and GTS conceived the idea, obtained funding, supervised the study, designed experiments, analyzed the data, performed statistics, analyzed public datasets, generated graphs and figures, wrote the original paper and its revised form, and is the guarantor of the study’s integrity. All authors reviewed and concur with the submitted manuscript.

## Conflict of interest

IP works as a Senior Director in AstraZeneca Pharmaceutical in a non‐related field with the publication. The remaining authors declare no competing financial interests.

## For more information

Institute of Lung Biology and Disease (ILBD) & Comprehensive Pneumology Center (CPC): https://www.helmholtz‐muenchen.de/ilbd/index.html

Helmholtz Center Munich‐German Research Center for Environmental Health (HMGU): https://www.helmholtz‐muenchen.de/en/helmholtz‐zentrum‐muenchen/index.html

Ludwig‐Maximilian‐University (LMU) Munich: https://www.en.uni‐muenchen.de/index.html

The Regional Center for Research in Cancerology and Immunology Nantes / Angers: https://www.crcina.org/?lang=en

The Koc University School of Medicine: https://medicine.ku.edu.tr/en/

The cancer genome atlas (TCGA) pan‐cancer human malignant pleural mesothelioma (MPM) dataset available at cBioportal: https://www.cbioportal.org/study/summary?id=meso_tcga_pan_can_atlas_2018

The cancer genome atlas (TCGA) pan‐cancer human malignant pleural mesothelioma (MPM) gene expression dataset available at: https://xenabrowser.net/datapages/?dataset=TCGA‐MESO.htseq_fpkm‐uq.tsv&host=https%3A%2F%2Fgdc.xenahubs.net&removeHub=https%3A%2F%2Fxena.treehouse.gi.ucsc.edu%3A443

The catalogue of somatic mutations in cancer (COSMIC) human MPM dataset: https://cancer.sanger.ac.uk/cosmic/browse/tissue?wgs=off&sn=pleura&ss=all&hn=mesothelioma&sh=&in=t&src=tissue&all_data=n

Human MPM datasets at Gene Expression Omnibus: https://www.ncbi.nlm.nih.gov/geo/query/acc.cgi?acc=GSE51024, https://www.ncbi.nlm.nih.gov/geo/query/acc.cgi?acc=GSE134349, https://www.ncbi.nlm.nih.gov/geo/query/acc.cgi?acc=GSE42977

Novel mouse MPM cell line and normal mesothelial cell RNA sequencing dataset at Gene Expression Omnibus: https://www.ncbi.nlm.nih.gov/geo/query/acc.cgi?&acc=GSE94415

Using Pleural Effusions to Diagnose Cancer (MAPED) study page at ClinicalTrials.gov: https://www.clinicaltrials.gov/ct2/show/NCT03319472?term=maped&draw=2&rank=1

Links to patient support, advocate, and charity organizations: https://www.mesotheliomagroup.com/, https://www.mesothelioma.com/, https://www.mesotheliomahelp.org/, https://www.asbestos.com/support/, https://mesothelioma.net/mesothelioma‐support/, https://www.curemeso.org/, https://www.mesotheliomahope.com/resources/cancer‐foundations/, https://www.mesothelioma.uk.com/.

## Supporting information



Appendix

Expanded View Figures PDF

Source Data for Expanded View/Appendix

Source Data for Figure 1

Source Data for Figure 2

Source Data for Figure 3

Source Data for Figure 4

Source Data for Figure 5

Source Data for Figure 7

Source Data for Figure 8

Source Data for Figure 9

## Data Availability

Affymetrix CytoScanHD Microarray data: GEO dataset GSE134349 (https://www.ncbi.nlm.nih.gov/geo/query/acc.cgi?acc=GSE134349). IonTorrent RNA sequencing data: GEO dataset GSE94415 (https://www.ncbi.nlm.nih.gov/geo/query/acc.cgi?&acc=GSE94415).
