# Supplementary material for: KRAS signaling in malignant pleural mesothelioma
Source: EMBO Mol Med. 2021 Dec 13;14(2):e13631. doi: 10.15252/emmm.202013631 (PMC8819314; doi:10.15252/emmm.202013631)
Supplement: Supplementary file 1 — Appendix [file EMMM-14-e13631-s005.pdf]

# APPENDIX

## KRAS signaling in malignant pleural mesothelioma

Antonia Marazioti, Anthi C Krontira, Sabine J Behrend, Georgia A Giotopoulou, Giannoula Ntaliarda, Christophe Blanquart, Hasan Bayram, Marianthi Iliopoulou, Malamati Vreka, Lilith Trassl, Mario A A Pepe, Caroline M Hackl, Laura V Klotz, Stefanie AI Weiss, Ina Koch, Michael Lindner, Rudolph A Hatz, Juergen Behr, Darcy E Wagner, Helen Papadaki, Sophia G Antimisiaris, Didier Jean, Sophie Deshayes, Marc Grégoire, Özgecan Kayalar, Deniz Mortazavi, Şükrü Dilege, Serhan Tanju, Suat Erus, Ömer Yavuz, Pınar Bulutay, Pınar Fırat, Ioannis Psallidas, Magda Spella, Ioanna Giopanou, Ioannis Lilis, Anne-Sophie Lamort, and Georgios T Stathopoulos

## Table of Contents

**Appendix Element** **Page**
Appendix Title Page………………………………………………………………… S1
Appendix Figure……………………………………………………………………….. S2
Appendix Tables…………………………………………………………….............. S3

## Appendix Figure

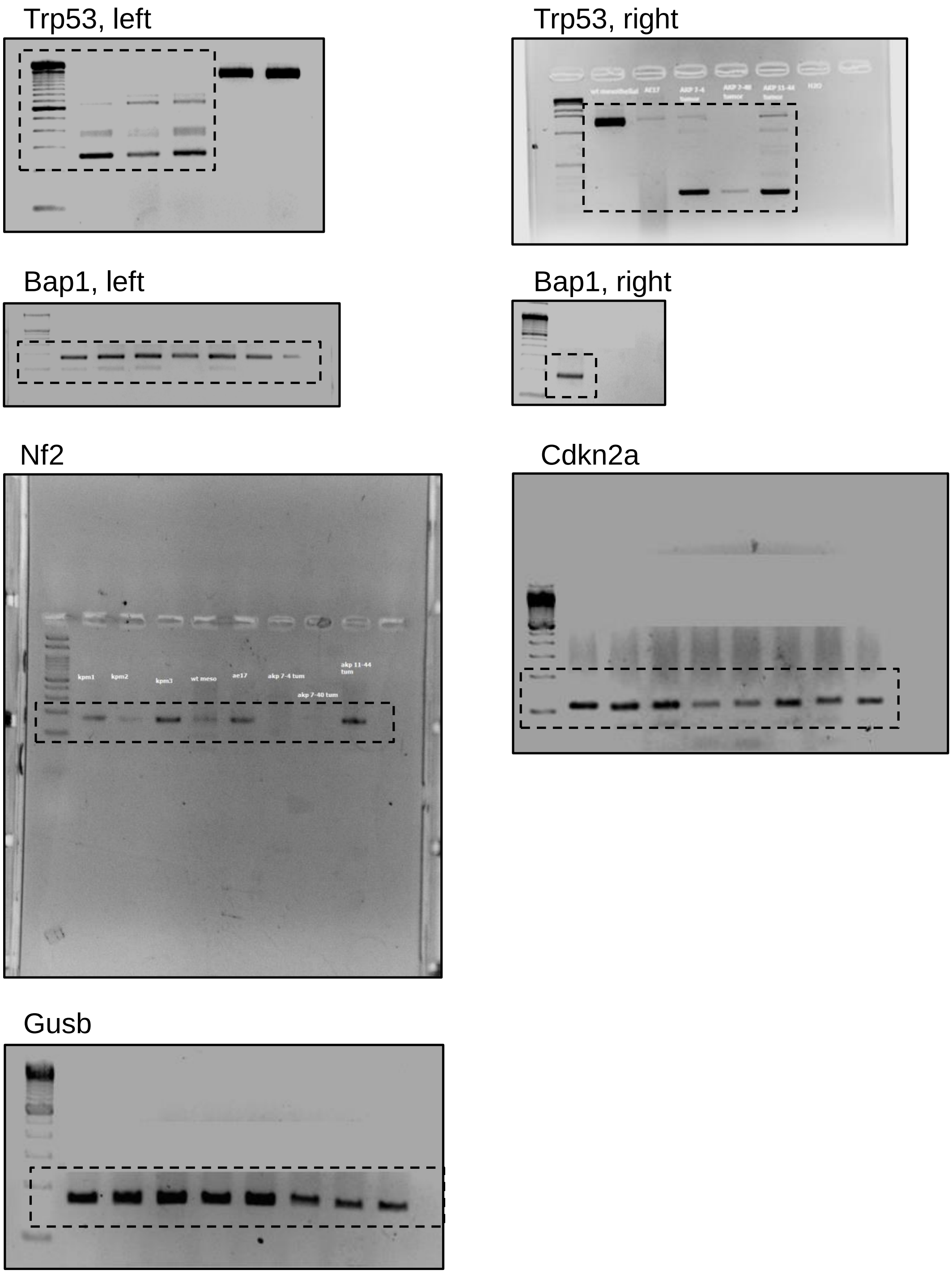


**Appendix Fig S1. Uncropped PCR gels from Fig 7E.**

Cropboxes indicate the gel areas shown in the main Figure.

# Appendix Tables

## Appendix Table S1. Clinical attributes of 45 patients with pleural effusions from Munich, Germany, studied by digital droplet (dd)PCR.

| Attribute | MPM | LUAD | Other cancers | Benign |
|---|---|---|---|---|
| Patient number (*n*) | 12 | 16 | 12 | 5 |
| Age (years)[a] | 71(68-77) | 72(56-75) | 63(50-75) | 62(21-86) |
| Sex (female/male; *n*) | 3/9 | 7/9 | 7/5 | 3/2 |
| Smoking status (never/ former/ current; *n*) | 8/4/0 | 10/4/2 | 9/0/3 | 3/0/2 |
| Smoking exposure (pack-years)[a] | 0(0-10) | 0(0-20) | 0(0-40) | 0(0-100) |
| Overall survival (years)[a] | 3.1(1.0-3.6) | 2.6(0.3-3.2) | 2.6(0.2-3.1) | 3.1(3.0-3.3) |
| Death events/ Censored observations (*n*) | 4/8 | 6/10 | 4/8 | 0/5 |
| *KRAS*$^{G12/13}$ droplets (%)[a] | 0(0-6) | 0(0-1) | 0(0-0) | 0(0-0) |
| *KRAS*$^{Q61}$ droplets (%)[a] | 0(0-0) | 0(0-0) | 0(0-8) | 0(0-0) |
| *TP53/TERT* droplets pleural fluid (%)[a] | 108(74-133) | 98(75-130) | 93(60-103) | 104(55-177) |
| *TP53/TERT* droplets pleural cells (%)[a] | 89(64-149) | 82(54-105) | 88(69-103) | 70(59-73) |
| Erythrocytes ($10^6/mm^3$)[a] | 4.2(1.4-27.8) | 1.9(1.2-32.3) | 8.5(1.2-32.0) | 1.9(0.8-9.0) |
| Nucleated cells ($10^3/mm^3$)[a] | 1.4(0.9-4.6) | 1.5(0.7-4.5) | 1.5(1.0-3.4) | 3.9(1.9-7.7) |
| Large mononuclear cells (%)[a] | 44(18-71) | 43(32-56) | 49(41-73) | 25(2-88) |
| Neutrophils (%)[a] | 2(0-7) | 9(3-11) | 7(2-29) | 2(2-98) |
| Small mononuclear cells (%)[a] | 47(6-64) | 44(23-54) | 31(10-53) | 18(0-73) |
| Eosinophils (%)[a] | 0(0-16) | 1(0-5) | 1(0-1) | 0(0-45) |

[a] Data are presented as median(95%confidence interval).

**Appendix Table S2. Clinical attributes of 33 donors of malignant pleural effusions due to malignant pleural mesothelioma from Nantes, France for establishment of cell lines studied by CytoScan arrays.**

| **Attribute** | |
|---|---|
| Patient number (*n*) | 33 |
| Age (years)[a] | 72(65-75) |
| Sex (female/male/unknown; *n*) | 6/26/1 |
| Asbestos exposure (yes/ no/ unknown; *n*) | 22/8/3 |
| MPM subtype (epithelioid/ biphasic/ sarcomatoid/ unspecified)[a] | 27/2/2/2 |
| Overall survival (years)[a] | 0.8(0.5-1.0) |
| Death events/ Censored observations (*n*) | 4/29 |

[a] Data are presented as median(95%confidence interval).

**Appendix Table S3. Clinical attributes of 10 donors of malignant pleural effusions due to lung adenocarcinoma (LUAD) and malignant pleural mesothelioma (MPM) from Nantes, France and of 17 donors of MPM tissues from Istanbul, Turkey, which were study by digital droplet PCR.**

| Attribute | Nantes LUAD | Nantes MPM | Istanbul MPM |
|---|---|---|---|
| Patient number (*n*) | 4 | 6 | 17 |
| Age (years)[a] | 64(49-70) | 66(56-85) | 67(54-70) |
| Sex (female/male/unknown; *n*) | 1/3 | 0/6 | 5/12 |
| Metastasis (yes/no; *n*) | 1/3/0 | 0/6 | 3/14 |
| MPM subtype (epithelioid/ biphasic/ sarcomatoid/ unspecified)[a] | - | 4/1/1/0 | 11/4/1/1 |

[a] Data are presented as median(95%confidence interval).

**Appendix Table S4. Top differentially expressed genes in *KRAS*$^{G12D}$*;Trp53f/f* malignant pleural mesothelioma cell lines (KPM) versus pleural mesothelial cells (PMC).**

| Gene symbol | Gene ID | *P* [a] | FDR *q* [b] | *Δ*GE [c] |
|---|---|---|---|---|
| *Fkbp10* | ENSMUSG00000001555 | 2,69E-06 | 3.66E-04 | +11,478 |
| *Cxcl5* | ENSMUSG00000029371 | 1,17E-06 | 1.27E-04 | +11,160 |
| *Tm4sf1* | ENSMUSG00000027800 | 7,75E-06 | 6.29E-04 | +11,090 |
| *Npdc1* | ENSMUSG00000015094 | 4,54E-06 | 4.12E-04 | +10,777 |
| *Etv4* | ENSMUSG00000017724 | 6,55E-06 | 5.49E-04 | +10,605 |
| *Cdc42ep1* | ENSMUSG00000049521 | 8,70E-06 | 6.92E-04 | +10,439 |
| *Gjb3* | ENSMUSG00000042367 | 2,32E-05 | 1.58E-03 | +10,230 |
| *Flnc* | ENSMUSG00000068699 | 1,44E-05 | 1.06E-03 | +10,153 |
| *Foxa2* | ENSMUSG00000037025 | 7,90E-05 | 4.17E-03 | +10,114 |
| *Cck* | ENSMUSG00000032532 | 2,37E-05 | 1.60E-03 | +9,950 |
| *C1qc* | ENSMUSG00000036896 | 6,77E-18 | 2,87E-14 | -12,792 |
| *C1qa* | ENSMUSG00000036887 | 1,57E-18 | 1,33E-14 | -13,319 |
| *Tyrobp* | ENSMUSG00000030579 | 2,12E-17 | 6,00E-14 | -13,392 |
| *Cd79b* | ENSMUSG00000040592 | 2,66E-17 | 6,45E-14 | -13,522 |
| *Lyz1* | ENSMUSG00000069515 | 2,81E-18 | 1,59E-14 | -14,652 |
| *Hbb-bs* | ENSMUSG00000052305 | 5,05E-05 | 2.89E-03 | -15,190 |
| *C1qb* | ENSMUSG00000036905 | 1,23E-18 | 1,33E-14 | -15,336 |
| *Hba-a2* | ENSMUSG00000069917 | 3,39E-06 | 3.20E-04 | -15,518 |
| *Hba-a1* | ENSMUSG00000069919 | 3,58E-07 | 4,82E-05 | -18,064 |
| *Hbb-bt* | ENSMUSG00000073940 | 1,55E-07 | 2,33E-05 | -18,539 |

[a] *P*, probability value by two-way ANOVA with Bonferroni post-test.

[b] FDR *q*, false discovery rate probability value.

[c] *Δ*GE, differential gene expression between KPM and PMC; a positive ΔGE indicates overexpression by KPM, while a negative ΔGE by PMC cells.

## Appendix Table S5. PCR primers used for these studies

| Method [a] | Primer | Sequence | Amplicon (bp) |
|---|---|---|---|
| Seq | KrasF1 | CCATTTCGGACCCGGAG | 904 |
| | KrasR1 | CTTTAGTCTCTTCCACAGGCA | |
| | KRASF2 | TCCCAGGTGCGGGAGAGAG | 846 |
| | KRASR2 | GCTAACAGTCTGCATGGAGCAGG | |
| | Bap1F | TATGCAATTGGCAATGCCCC | 928 |
| | Bap1R | CTGACAGCTGCCCATCTGAA | |
| RT-PCR | Trp53F | GTAGCTTCAGTTCATTGGGA | 1450 |
| | Trp53R | GAAGTCATAAGACAGCAAGGA | |
| | TP53F | CGCAGTCAGATCCTAGCGTC | 551 |
| | TP53R | ACCATCGCTATCTGAGCAGC | |
| RT-PCR & qPCR | MycoplasmaF | GGGAGCAAACAGGATTAGATACCCT | 270 |
| | MycoplasmaR | TGCACCATCTGTCACTCTGTTAACCTC | |
| | GusbF | TTACTTTAAGACGCTGATCACC | 165 |
| | GusbR | ACCTCCAAATGCCCATAGTC | |
| | Nf2F | CTGAAAATGCTGAGGAGGAGC | 155 |
| | Nf2R | ATAGTCGCCATACTTGGCCTG | |
| | Bap1F | TGCCATCAGATACAAGCGGA | 173 |
| | Bap1R | CAGCCCCACTGCTAGTCTTG | |
| | Cdkn2aF | CGAACTCTTTCGGTCGTACCC | 102 |
| | Cdkn2aR | GGTCCTCGCAGTTCGAATCTG | |
| | GUSBF | CTACTTGAAGATGGTGATCGCTC | 118 |
| | GUSBR | ACAGATCACATCCACATACGG | |
| | TP53F | AGTCTACCTCCCGCCATAAA | 173 |
| | TP53R | GCTTCTGACGCACACCTATT | |

[a] Method: Seq, sequencing; RT-PCR, reverse transcriptase polymerase chain reaction; qPCR, quantitative real-time polymerase chain reaction.

## Appendix Table S6. Antibodies used for these studies

| Method [a] | Target [b] | Provider [c] | Catalog # | Dilution | Conjugate [d] |
|---|---|---|---|---|---|
| IHC | PCNA | Abcam | ab2426 | 1:2000 | - |
| | CAL | Abcam | ab203055 | 1:400 | - |
| | PDPN | Merck | ABT34 | 1:1000 | - |
| | OPN | Abcam | ab8448 | 1:100 | - |
| | KRT5/6 | Merck | MAB3412 | 1:200 | - |
| | SFTPC | Santa Cruz | FL-197 | 1:100 | - |
| | BAP1 | Santa Cruz | Sc-28383 | 1:100 | - |
| | WT1 | Bioworld | BS6239 | 1:100 | - |
| | VIM | Elabscience | E-AB-36172 | 1:1000 | - |
| | MSLN | Biomol | V3821-20UG | 1:100 | - |
| FC | CD11b | eBioscience | 12-0112 | 0.1μg/$10^6$cells | PE |
| | Gr1 | eBioscience | 25-5931-82 | 0.1μg/$10^6$cells | PE-Cy7 |

[a] Method: IHC, immunohistochemistry; FC,flow cytometry.

[b] Target: PCNA, proliferating cell nuclear antigen; CAL, calretinin; PDPN, podoplanin; OPN, osteopontin; KRT, cytokeratin; SFTPC, surfactant protein C; BAP1, *BRCA1* associated protein-1; CD11b, cluster of differentiation molecule 11B; Gr1, Gr1 antigen.

[c] Provider*:* Abcam, Cambridge, UK; eBioscience, San Diego, CA; Santa Cruz Biotechnology, Santa Cruz, CA; Merck Millipore, Darmstadt, Germany; Bioworld, Philadelphia, PA; Elabscience, Houston, TX; Biomol, Hamburg, Germany.

[d] Conjugate*:* PE, phycoerythrin; PE-Cy7, phycoerythrin-cyanin 7.

## Appendix Table S7. Number of experimental mice (*n*) used for these studies

| Strain designation | Jackson Laboratory ID # | Short strain designation | *n* |
|---|---|---|---|
| *C57BL/6J* | 000664 | *C57BL/6* | 177 |
| B6.129(Cg)-Gt(ROSA)26Sor$^{tm4(ACTB\text{-}tdTomato,\text{-}EGFP)Luo}$/J | 007676 | *mT/mG* | 40 |
| B6.129S4-*Kras*$^{tm4Tyj}$/J | 008179 | *KRAS*$^{G12D}$ | 40 |
| B6.129P2-*Trp53*$^{tm1Brn}$/J | 008462 | Trp53f/f | 33 |
| FVB-Tg(CAG-luc,-GFP)L2G85Chco/J | 008450 | CAG.Luc.eGFP | 5 |
| Intercrossed mice | | *Trp53f/Wt* | 11 |
| | | *KRAS*$^{G12D}$*;Trp53f/Wt* | 11 |
| | | *KRAS*$^{G12D}$*;Trp53f/f* | 115 |
| **Total** | | | **432** |
