# Supplementary material for: KRAS signaling in malignant pleural mesothelioma
Source: EMBO Mol Med. 2021 Dec 13;14(2):e13631. doi: 10.15252/emmm.202013631 (PMC8819314; doi:10.15252/emmm.202013631)
Supplement: Supplementary file 2 — Expanded View Figures PDF [file EMMM-14-e13631-s008.pdf]

Expanded View Figures

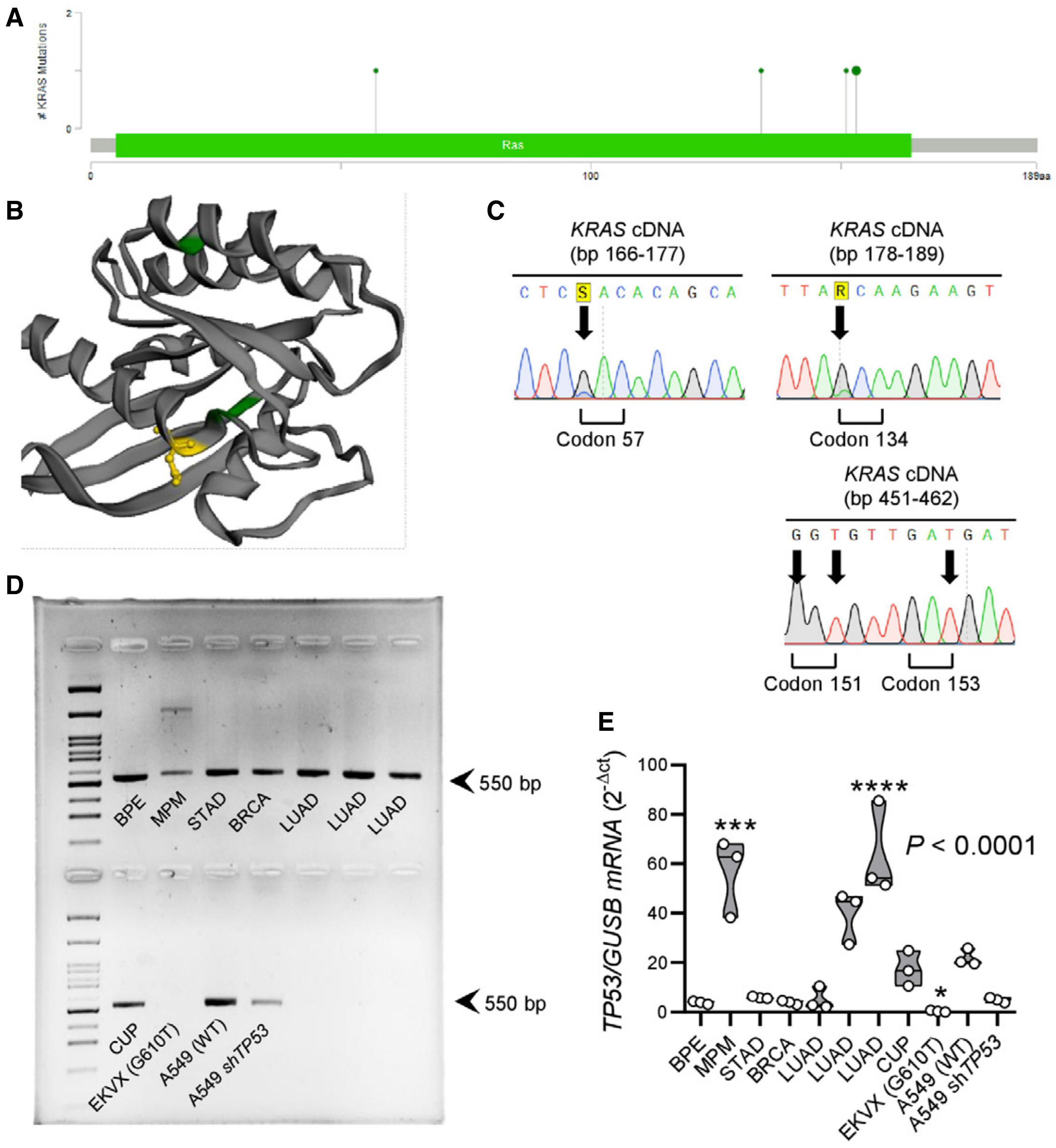

Figure EV1. KRAS and TP53 alterations in a patient with malignant pleural mesothelioma from the MAPED study (NCT03319472).

A Lollipop plot showing the four different missense *KRAS* mutations found (D57H, A134T, R151G, and E153D).

B 3D rendering of KRAS protein showing the three *KRAS* mutations predicted by OncoKB to be non-functional (green color) and the E153D mutation predicted by OncoKB to be oncogenic (yellow color).

C Representative Sanger sequencing traces. Arrows indicate point mutations.

D *TP53* RT–PCR in comparison to cancer cell lines and other patients with malignant pleural effusion. Note the decreased band intensity in the patient with MPM.

E *TP53* qPCR in comparison to cancer cell lines and other patients with malignant pleural effusion. Note the markedly increased *TP53* transcript abundance in the patient with MPM that, together with (D), indicates a *TP53* mutation.

Data information: In (A), the likely oncogenic E153D mutation is shown enlarged compared with the other three mutations. In (D), arrows at 550 base pairs (bp) indicate amplicon size. In (E), data are presented as raw data points (circles), rotated kernel density distributions (violins), and medians (lines). *P*, overall probability, one-way ANOVA. *, ***, and ****: $P < 0.05$, $P < 0.001$, and $P < 0.0001$ compared with A549 cells, Bonferroni post-tests. In (D) and (E), abbreviations are as follows: BPE, benign pleural effusion; MPM, malignant pleural mesothelioma; STAD, stomach adenocarcinoma; BRCA, breast cancer; LUAD, lung adenocarcinoma; CUP, cancer of unknown primary; sh, cell line stably expressing anti-*TP53* short hairpin RNA. The TP53 status of the EKVX and A549 cell lines is indicated in parentheses.

Source data are available online for this figure.

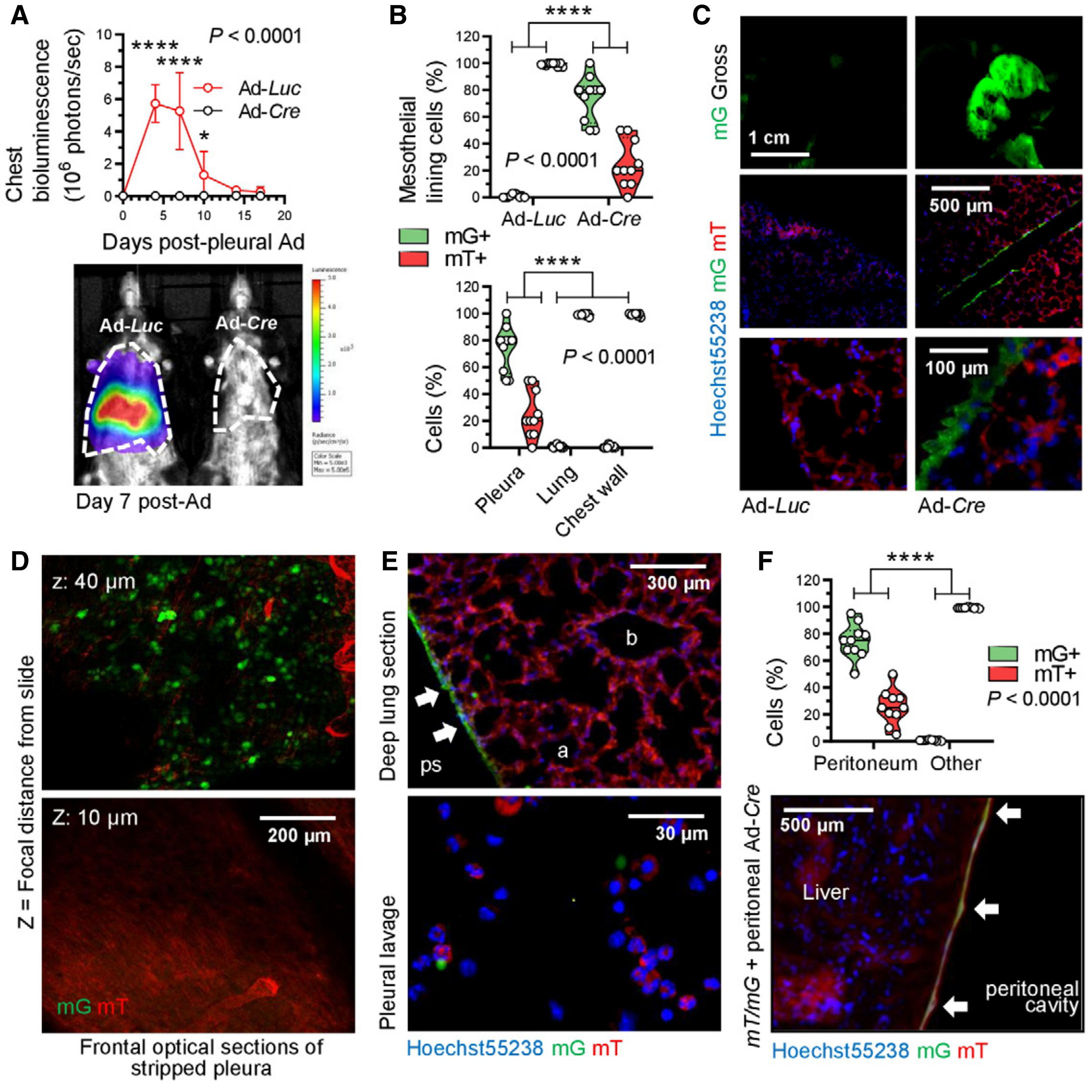

Figure EV2. Adenoviral-mediated mesothelial recombination.

Dual-fluorescent *mT*/*mG* CRE-reporter mice (*C57BL/6* background) received 5×10^8 PFU intrapleural (A–E) or intraperitoneal (F) Ad-*Luc* or Ad-*Cre* and were serially imaged for bioluminescence.

- A Data summary of chest light emission (top; *n* = 5 mice/group) and representative bioluminescence images (bottom). Note cessation of transient Ad-*Luc* expression by day 14.
- B Data summary of mG^+ and mT^+ mesothelial, lung, and chest wall cell percentage (*n* = 10 mice/group; B), representative macroscopic (top) and microscopic (middle, bottom) fluorescent images (C), optical frontal sections of stripped parietal pleura placed apical side up on glass slides (D), and deep lung sections (E, top) and fluorescent image of pleural lavage cells (E, bottom). *z*, focal plane distance from slide. a, alveoli; b, bronchi; ps, pleural space; arrows, recombined mesothelium.
- C Data summary of mG^+ and mT^+ mesothelial and deeper located (other) abdominal cell percentage (*n* = 10 mice/group) and representative merged microscopic fluorescent image of peritoneal surface mesothelium showing *Cre*-recombined mesothelium (arrows).

Data information: In (A), data are presented as mean ± 95% confidence interval. *P*, overall probability, two-way ANOVA. * and ****: $P < 0.05$ and $P < 0.0001$ for comparison between groups at the indicated time points, Bonferroni post-tests. In (B) and (F), data are presented as raw data points (circles), rotated kernel density distribution (violins), and medians (lines). *P*, overall probability, two-way ANOVA. ****: $P < 0.0001$ for the indicated comparisons, Bonferroni post-tests. Ad, adenovirus; PFU, plaque-forming units; *Luc*, luciferase gene; *Cre*, CRE recombinase gene; *mT*, membranous tomato red; *mG*, membranous green fluorescent protein.

Source data are available online for this figure.

 © 2021 The Authors

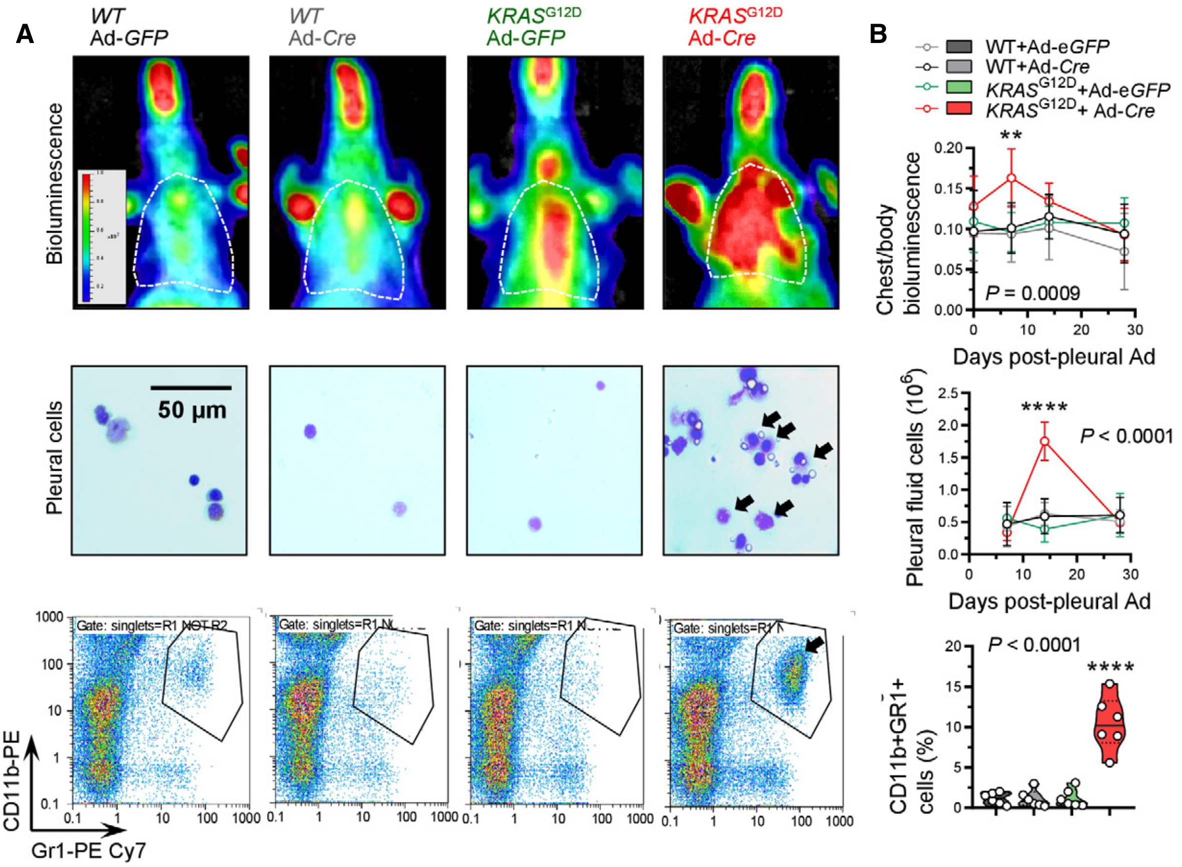

Figure EV3. Pleural mesothelial *KRAS*^{G12D} expression causes inflammation.

Wild-type (*WT*) and *KRAS*^{G12D} mice were lethally irradiated (1,100 Rad) and received same-day bone marrow transfer of 10 million bone marrow cells from ubiquitously luminescent *CAG.Luc.eGFP* donors (all on the *C57BL/6* strain). After 1 month required for bone marrow reconstitution, chimeras received 5×10^8 PFU intrapleural Ad vectors, were longitudinally imaged for bioluminescence, and were sacrificed for pleural lavage cell analysis.

A Representative chest bioluminescence images taken 2 weeks post-Ad (top), pleural lavage cytocentrifugal specimens stained with May–Gruenwald–Giemsa (middle), and dotplots of CD11b and Gr1 expression by flow cytometry (bottom). Dotted lines in top panels denote the chest. Arrows in middle and bottom panels indicate increased mononuclear cells.

B Summary of longitudinal chest light emission and total pleural cell number (dotplots), legend to dotplots, as well as of CD11b$^+$Gr1$^+$ pleural cells at day 14 post-Ad (violin plot).

Data information: In (B), data are presented as mean ± 95% confidence interval (dotplots; n = 5–6 mice/data-point) or as raw data points (circles), rotated kernel density distribution (violins), and medians (lines). *P*, overall probability, one-way (violin plot) or two-way (dotplots) ANOVA. ** and ****: $P < 0.01$ and $P < 0.0001$, respectively, for Ad-*Cre*-treated *KRAS*^{G12D} mice compared with all other groups, Bonferroni post-tests. *WT*, wild-type; *KRAS*^{G12D}, Lox-STOP-Lox.*KRAS*^{G12D}; *CAG. Luc.eGFP*, ubiquitously luminescent mice; Ad, adenovirus type 5; PFU, plaque-forming units; *Cre*, CRE recombinase gene; GFP, green fluorescent protein; ANOVA, analysis of variance.

Source data are available online for this figure.

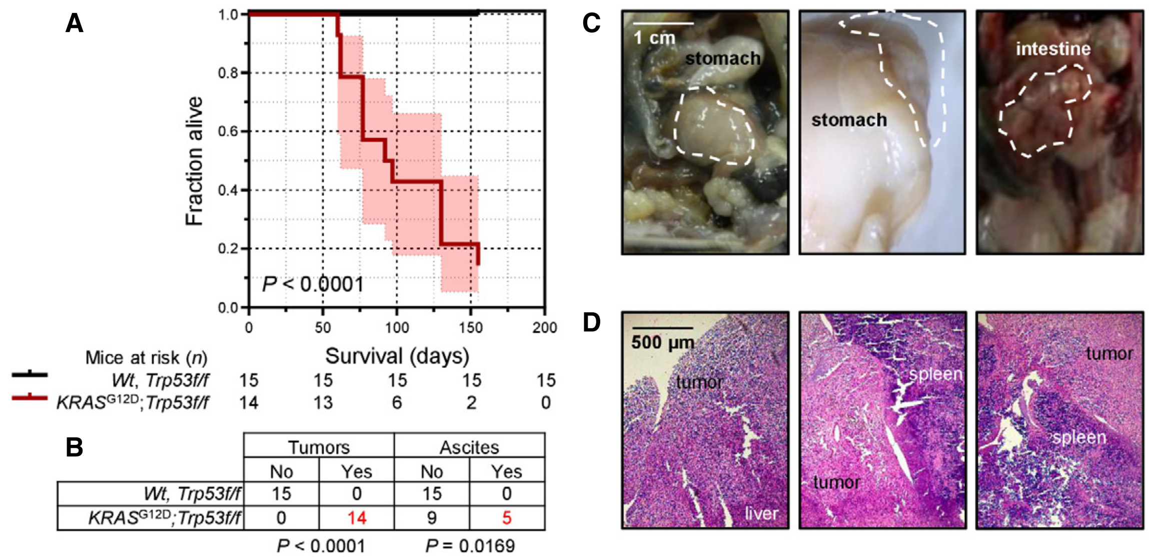

Figure EV4. Malignant peritoneal mesothelioma of *KRAS*^{G12D}*;Trp53f/f* mice.

Wild-type (*Wt*), *Trp53f/f*, and *KRAS*^{G12D}*;Trp53f/f* mice (all *C57BL/6*) received 5 × 10^8 PFU intraperitoneal Ad-*Cre* and were harvested when moribund.

A Kaplan–Meier survival plot and survival table.

B Tumor and ascites incidence table.

C Representative macroscopic images of peritoneal tumors (dashed outlines).

D Representative hematoxylin-and-eosin-stained tissue sections of peritoneal tumors.

Data information: In (A), data are presented as Kaplan–Meier survival estimates (lines), 95% confidence intervals (shaded areas) and numbers of mice at risk. *P*, probability, log-rank test. In (B), data are presented as number of mice (*n*). *P*, probabilities, Fischer's exact tests. *Wt*, wild-type; *KRAS*^{G12D}, Lox-STOP-Lox.*KRAS*^{G12D}; *Trp53f/f*, conditional *Trp53*-deleted; Ad, adenovirus type 5; PFU, plaque-forming units; *Cre*, CRE recombinase gene.

Source data are available online for this figure.

Figure EV5. Bap1 mutations of KPM cells.

KRAS^{G12D}*;Trp53f/f* pleural mesothelioma (KPM) and pleural mesothelial cells (PMC) were analyzed by RNA sequencing (GEO dataset GSE94415), Sanger sequencing for *Bap1*, and immunohistochemistry for BAP1 protein expression.

A Coverage and alignment plot from RNA sequencing. Alignments are represented as gray polygons with reads mismatching the reference indicated by color. Loci with a large percentage of mismatches relative to the reference are flagged in the coverage plot as color-coded bars. Alignments with inferred small insertions or deletions are represented with vertical or horizontal bars, respectively.

B *Bap1* mRNA Sanger sequencing shows a G>A transition (arrow) at c.829 that generates a missense mutation in codon E278K (top), as well as a single nucleotide insertion in position c.831 with a consequent frameshift mutation in codon S279insA and a single nucleotide insertion resulting to a frameshift mutation in codon K340insA at c.1072 (bottom).

C Representative immunohistochemical images of BAP1 immunoreactivity (brown) of lungs with normal PMC and mouse tumors caused by transplanted KPM cells counterstained with hematoxylin (blue). Arrows indicate nuclear BAP1 staining.

D Lollipop plot for each KPM cell line visualizing all *Bap1* mutations detected. Red and blue lollipops with their numbers represent, respectively, missense mutations and insertions causing frameshift with their positions after the ATG start codon.

▶

 © 2021 The Authors

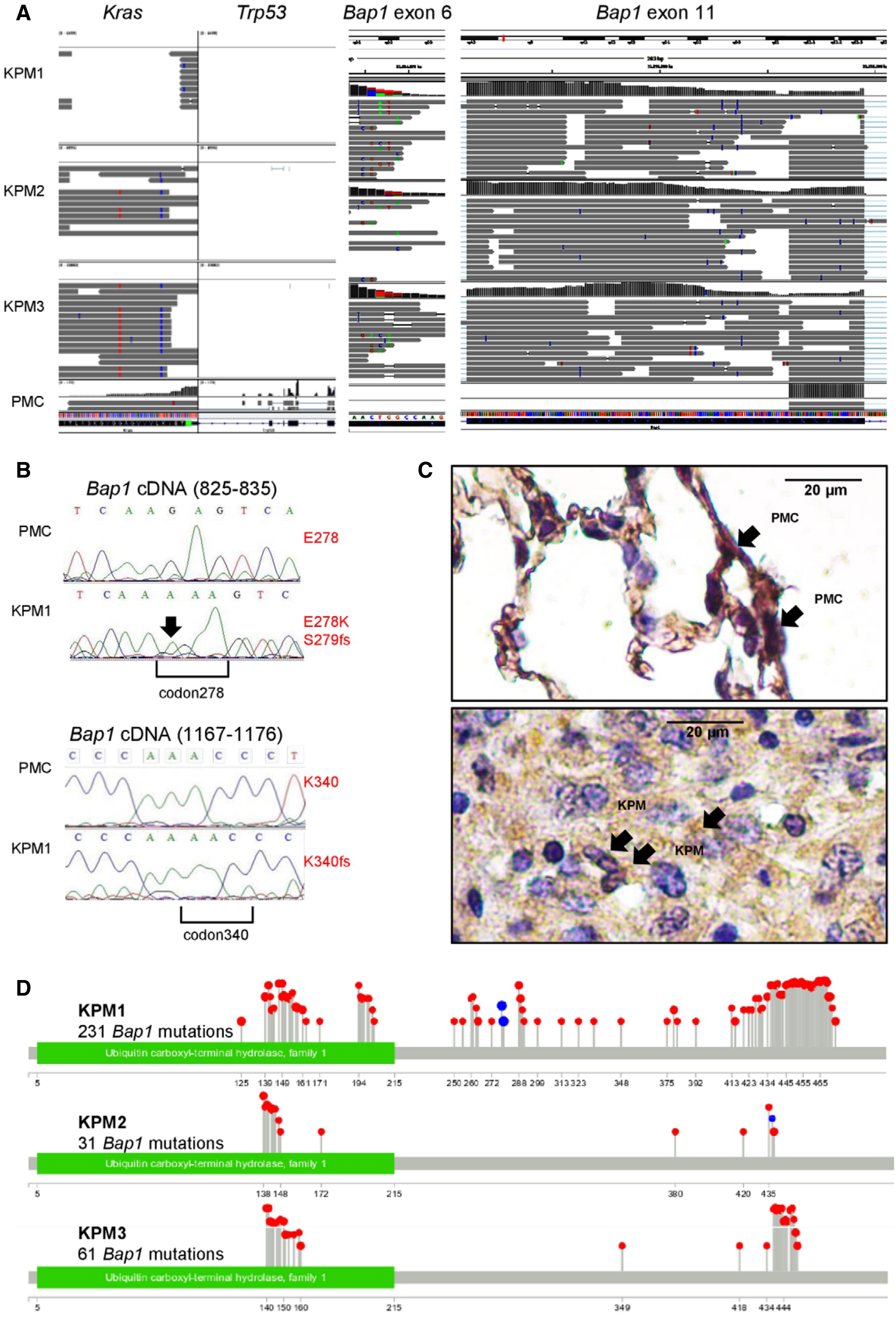
A
Kras
Trp53
Bap1 exon 6
Bap1 exon 11
KPM1
KPM2
KPM3
PMC
B
Bap1 cDNA (825-835)
PMC
E278
KPM1
E278K
S279fs
codon278
Bap1 cDNA (1167-1176)
PMC
K340
KPM1
K340fs
codon340
C
20 μm
PMC
PMC
20 μm
KPM
KPM
D
KPM1
231 Bap1 mutations
Ubiquitin carboxyl-terminal hydrolase, family 1
KPM2
31 Bap1 mutations
Ubiquitin carboxyl-terminal hydrolase, family 1
KPM3
61 Bap1 mutations
Ubiquitin carboxyl-terminal hydrolase, family 1

Figure EV5.
