# Supplementary material for: KRAS signaling in malignant pleural mesothelioma
Source: EMBO Mol Med. 2021 Dec 13;14(2):e13631. doi: 10.15252/emmm.202013631 (PMC8819314; doi:10.15252/emmm.202013631)
Supplement: Supplementary file 12 [file EMMM-14-e13631-s009.pdf]

EMBO Molecular Medicine

KRAS signaling in malignant pleural mesothelioma

Antonia Marazioti, Anthodesmi Krontira, Sabine Behrend, Georgia Giotopoulou, Gianna Ntaliarda, Christophe Blanquart, Hasan Bayram, Mario Pepe, Caroline Hackl, Marianthi Iliopoulou, Malamati Vreka, Lilith Trassl, Laura Klotz, Stefanie Weiss, Ina Koch, Michael Lindner, Rudolph Hatz, Jürgen Behr, Darcy Wagner, Helen Papadaki, Sophia Antimisiaris, Didier Jean, Sophie Deshayes, Marc Gregoire, Özgecan Kayalar, Deniz Mortazavi, Sükrü Dilege, Serhan Tanju, Suat Erus, Ömer Yavuz, Pinar Bulutay, Pinar Firat, Ioannis Psallidas, Magda Spella, Ioanna Giopanou, Ioannis Lilis, Anne Sophie Lamort, and Georgios Stathopoulos
DOI: 10.15252/emmm.202013631

Corresponding author: Georgios Stathopoulos (stathopoulos@helmholtz-muenchen.de)

Review Timeline:

Submission Date:	22nd Oct 20
Editorial Decision:	17th Nov 20
Revision Received:	4th Apr 21
Editorial Correspondence:	22 Apr 21
Author Correspondence:	23rd Apr 21
Editorial Decision:	23rd Apr 21
Revision Received:	22nd Sep 21
Editorial Decision:	12th Oct 21
Revision Received:	28th Oct 21
Editorial Correspondence:	12th Nov 21
Accepted:	15th Nov 21

Editor: Lise Roth

Transaction Report:

(Note: With the exception of the correction of typographical or spelling errors that could be a source of ambiguity, letters and reports are not edited. Depending on transfer agreements, referee reports obtained elsewhere may or may not be included in this compilation. Referee reports are anonymous unless the Referee chooses to sign their reports.)

17th Nov 2020

Dear Dr. Stathopoulos,

Thank you for the submission of your manuscript to EMBO Molecular Medicine. We have now received feedback from the three reviewers who agreed to evaluate your manuscript. As you will see from the reports below, the referees acknowledge the interest of the study and are overall supporting publication of your work pending appropriate revisions.

Addressing the reviewers' concerns in full will be necessary for further considering the manuscript in our journal, and in particular, the implications of your findings for human mesothelioma should be further clarified (but not overstated). Acceptance of the manuscript will entail a second round of review. EMBO Molecular Medicine encourages a single round of revision only and therefore, acceptance or rejection of the manuscript will depend on the completeness of your responses included in the next, final version of the manuscript. For this reason, and to save you from any frustrations in the end, I would strongly advise against returning an incomplete revision.

***

When submitting your revised manuscript, please carefully review the instructions that follow below. Failure to include requested items will delay the evaluation of your revision:

1) A .docx formatted version of the manuscript text (including legends for main figures, EV figures and tables). Please make sure that the changes are highlighted to be clearly visible.

2) Individual production quality figure files as .eps, .tif, .jpg (one file per figure).

3) A .docx formatted letter INCLUDING the reviewers' reports and your detailed point-by-point responses to their comments. As part of the EMBO Press transparent editorial process, the point-by-point response is part of the Review Process File (RPF), which will be published alongside your paper.

4) A complete author checklist, which you can download from our author guidelines (https://www.embopress.org/page/journal/17574684/authorguide#submissionofrevisions). Please insert information in the checklist that is also reflected in the manuscript. The completed author checklist will also be part of the RPF.

5) Before submitting your revision, primary datasets produced in this study need to be deposited in an appropriate public database (see https://www.embopress.org/page/journal/17574684/authorguide#dataavailability).
Please remember to provide a reviewer password if the datasets are not yet public.
The accession numbers and database should be listed in a formal "Data Availability " section (placed after Materials & Method). Please note that the Data Availability Section is restricted to new primary data that are part of this study.

*** Note - All links should resolve to a page where the data can be accessed. ***

6) We would also encourage you to include the source data for figure panels that show essential data. Numerical data should be provided as individual .xls or .csv files (including a tab describing the data). For blots or microscopy, uncropped images should be submitted (using a zip archive if multiple images need to be supplied for one panel). Additional information on source data and instruction on how to label the files are available at
.

7) Our journal encourages inclusion of *data citations in the reference list* to directly cite datasets that were re-used and obtained from public databases. Data citations in the article text are distinct from normal bibliographical citations and should directly link to the database records from which the data can be accessed. In the main text, data citations are formatted as follows: "Data ref: Smith et al, 2001" or "Data ref: NCBI Sequence Read Archive PRJNA342805, 2017". In the Reference list, data citations must be labeled with "[DATASET]". A data reference must provide the database name, accession number/identifiers and a resolvable link to the landing page from which the data can be accessed at the end of the reference. Further instructions are available at .

8) We replaced Supplementary Information with Expanded View (EV) Figures and Tables that are collapsible/expandable online. A maximum of 5 EV Figures can be typeset. EV Figures should be cited as 'Figure EV1, Figure EV2" etc... in the text and their respective legends should be included in the main text after the legends of regular figures.

- For the figures that you do NOT wish to display as Expanded View figures, they should be bundled together with their legends in a single PDF file called *Appendix*, which should start with a short Table of Content. Appendix figures should be referred to in the main text as: "Appendix Figure S1, Appendix Figure S2" etc.

- Additional Tables/Datasets should be labeled and referred to as Table EV1, Dataset EV1, etc. Legends have to be provided in a separate tab in case of .xls files. Alternatively, the legend can be supplied as a separate text file (README) and zipped together with the Table/Dataset file.
See detailed instructions here:
.

9) The paper explained: EMBO Molecular Medicine articles are accompanied by a summary of the articles to emphasize the major findings in the paper and their medical implications for the non-specialist reader. Please provide a draft summary of your article highlighting
- the medical issue you are addressing,
- the results obtained and
- their clinical impact.

This may be edited to ensure that readers understand the significance and context of the research. Please refer to any of our published articles for an example.

10) For more information: There is space at the end of each article to list relevant web links for further consultation by our readers. Could you identify some relevant ones and provide such information as well? Some examples are patient associations, relevant databases, OMIM/proteins/genes links, author's websites, etc...

11) Every published paper now includes a 'Synopsis' to further enhance discoverability. Synopses are displayed on the journal webpage and are freely accessible to all readers. They include a short stand first (maximum of 300 characters, including space) as well as 2-5 one-sentences bullet points that summarizes the paper. Please write the bullet points to summarize the key NEW findings. They should be designed to be complementary to the abstract - i.e. not repeat the same text. We encourage inclusion of key acronyms and quantitative information (maximum of 30 words / bullet point). Please use the passive voice. Please attach these in a separate file or send them by email, we will incorporate them accordingly.

Please also suggest a striking image or visual abstract to illustrate your article. If you do please provide a png file 550 px-wide x 400-px high.

12) As part of the EMBO Publications transparent editorial process initiative (see our Editorial at http://embomolmed.embopress.org/content/2/9/329), EMBO Molecular Medicine will publish online a Review Process File (RPF) to accompany accepted manuscripts.
In the event of acceptance, this file will be published in conjunction with your paper and will include the anonymous referee reports, your point-by-point response and all pertinent correspondence relating to the manuscript. Let us know whether you agree with the publication of the RPF and as here, if you want to remove or not any figures from it prior to publication.
Please note that the Authors checklist will be published at the end of the RPF.

EMBO Molecular Medicine has a "scooping protection" policy, whereby similar findings that are published by others during review or revision are not a criterion for rejection. Should you decide to submit a revised version, I do ask that you get in touch after three months if you have not completed it, to update us on the status.

I look forward to receiving your revised manuscript.

Yours sincerely,

Lise Roth

Lise Roth, PhD
Editor
EMBO Molecular Medicine

***** Reviewer's comments *****

Referee #1 (Comments on Novelty/Model System for Author):

In this work investigators look at KRAS alterations as potential initiating event for malignant mesothelioma. They look at KRAS alterations in a series of mesothelioma samples and find a minority have mutations or amplification. Using an AdCRE system they deliver the KRAS alteration to the pleural cavity of mice and pleural changes resembling mesothelioma develop over many months. By altering p53 as well, mice develop a more biphasic histology. BAP1 alterations occur secondarily. They postulate the reason for the low KRAS mutations seen in clinical specimens is low sensitivity of the assays and regression of clones that are KRAS altered over time in favor of the clones. this reviewer agrees that a subset of mesotheliomas probably exist that maybe KRAS driven.

My comments:

1- In terms of the clinical specimens pathway analyses using RNA seq to determine which pathways are activated may be helpful. For example in the KRAS mutated are the relevant pathways showing activation. This data should be available from expression values. Are the KRAS mutated mesotheliomas showing activation of the pathway. As they are mostly seen in epithelioid variety does this effect prognosis. More evaluation of evaluation of clinical specimens would be helpful

2- Although they report copy number alteration the actual copy number values is required. Many of the alterations seem low copy number which diminish their clinical value. What are the co-mutations in the KRAS altered mesotheliomas as opposed to to other mesotheliomas.

3- If in fact KRAS alterations are in only a sub clone of specimens (and this number is very low) which requires highly sensitive techniques and increased depth of coverage how can this be clinically important.

4- The discussion should be around discussing a novel subgroup of mesotheliomas that maybe KRAS driven (lung lung cancer) and we should try to identify these patients. Most malignancies are being subgroup by genomic alterations and subgroups being targeted for therapeutic vulnerabilities. Therefore a hierirachal clustering of mesotheliomas that are KRAS driven or not should be performed (from the clinical specimens already out there in the literature).

5- The over emphasis on the presence of effusions in mesothelioma is wrong. Many mesotheliomas do not have effusions and this is not a diagnostic criteria.

Referee #2 (Comments on Novelty/Model System for Author):

The human data are highly hypothetical, not supported by the data

Referee #2 (Remarks for Author):

The information in the literature relating RAS alterations to mesothelioma:
There is an extreme paucity of publications supporting the relevance of somatic RAS mutations in human mesothelioma. A few papers report RAS pathway activation predominantly in cell lines or mouse data. Recently a Nature Communication paper reported the involvement of RAS in causing pleural effusions in mice, the study is based on the use of cell lines and combines

data from adenocarcinoma and mesothelioma together. Unfortunately that study includes only one MPM murine cell line as a representative of the MPM tumor type. Large sequencing studies including Bueno et al, did not find a significant amount of mutations in the RAS pathway, however KRAS pathway up-regulation was identified by mRNA analysis in some specimens.

In this paper I am reviewing, the authors utilize a very sensitive technique, the digital droplet PCR to detect mutations in a small fraction of tumor cells to detect alterations in KRAS in human pleural mesothelioma samples. In a cohort of 12 mesothelioma samples they found 1 sample with a frequency of RAS mutation < 10% and 2 with frequency <1%. While this new technique has proven to be sensitive and specific (30504843, 31477768) to detect mutations in cell subpopulations, the number of samples analyzed here is too small to make conclusions about representation of RAS mutation in human MPM at large.

The authors then analyze 32 patient-derived MPM cell lines by CytoScan, a SNP-based sensitive technique to detect CNV (the sensitivity of this technique should be similar to a high density microarray). Although this dataset shows that the technique used is sensitive, the use of cell lines limits the relevance of these findings, as it is well known that cell lines accumulate genetic damage in tissue culture that is not representative of the initial genetic damage that caused tumor growth. primary mesothelioma biopsies should have been analyzed by CytoScan to support the cell line results. A characterization of patient characteristics and the cellular content of the malignant pleural effusion used to generate the data with ddPCR and CytoScan should also be included

To support their findings, the authors analyze previously published WES data sets from human mesothelioma samples (fig. 1d) from refs 13-21 and conclude that about 10% of the samples carried RAS mutations. This finding is potentially interesting especially because it was not noted or described by the authors of those studies, did they really miss it?. Given that the Authors of this paper I am reviewing have an "additional" interpretation of the data presented in refs 13-21, they authors should include a summary table describing all the RAS mutations found in each study, the frequency in each dataset, the total number of samples per dataset, the specific type of mutations, gene (chromosome/cDNA location) and their putative pathogenicity score. If these mutations were not listed in the previous publications (main paper or supplementary data), the authors should also include a description of the method used to analyze the raw data and mention the databases used to access those data. In toehr words they need to explain us how and why they re-interpreted these data to conclude that pathogenic RAS mutations are relatively frequent.

The mouse data presented are instead convincing. The authors demonstrated that RAS alone and more potently in combination with TP53 mutations promote mesothelioma in mice. To see if the mouse data are relevant to human mesothelioma, the authors should investigate whether concurrent TP53 and KRAS mutations are present in human mesotheliomas (ddPCR can be easily multiplexed as published in 30504843)
The authors should also use the previously published data they refer to (refs.13-21 shown in fig 1d) and maybe the TGCA dataset to find out if there is any overlap between TP53 mutations and RAS mutations in clinical mesothelioma samples.

The conclusive hypothesis drawn by the authors that "Mesothelial KRAS and other RAS pathway mutations may initiate MPM in some patients, but may be subsequently lost during tumour evolution, or may persist at a clonal level going undetected during sampling/sequencing, as has been shown for lung tumours51,52" and depicted in fig.9h, is highly hypothetical as it is not substantiated by the data presented. Presently these data show that RAs mutations together with TP53 mutations cause mesothelioma in mice. The human data are too preliminary to be presented.

SPECIFIC EDITS
Page 6 line 4 from bottom. Fig. reference: should be Fig. 3a,b and not 2,a b

Some of the literature cited about mesothelioma is old and does not reflect current knowledge, for example reference 1-2 used as general reference for the epidemiology of mesothelioma, references 9-12 used for "predictive factors of survival". Rather use recent reviews published in 2019 and 2020 in some of the top high impact factor medical and scientific journals.

Referee #3 (Remarks for Author):

The authors perform characterization of patient mesothelioma samples derived from pleural effusions, finding that 2 of 12 patients have a KRAS oncogenic driver mutation and in as separate cohort of cell lines that copy number events at this locus are also frequent. They then use a KRas/Trp53 mutant mouse model to demonstrate the formation of mesothelioma when those genes are altered in pleura. The manuscript is a significant step forward in mouse modelling of mesothelioma, is well-written and I have only relatively minor suggestions for improvements.

Comments:
• The authors could provide more context for their findings by clearly stating at the outset what proportions of mesothelioma patients are thought to have KRAS, TP53, BAP1 etc. mutations based on prior large genomic sequencing studies (TCGA etc).
• The claim in the abstract that "a significant proportion of human MPM harbour point mutations and copy number alterations in the KRAS proto-oncogene" might require validation in larger cohorts.
• Why are a different patient cohort used for the ddPCR and Affymetrix CytoScanHD assays? What are the KRAS mutation

statuses of the cell lines?
• Additional markers such as mesothelin, WT1 and vimentin could be added to the characterisation in Figure 6 for a more comprehensive assessment.
• Figure 8D - do these mutations actually effect BAP1 function? In this panel what do blue vs red dots represent? Do the mutations affect BAP1 nuclear localisation (this is more commonly used as a read out than sequencing in mesothelioma).
• Figure 8A is unreadably small as presented.
• Based on KRAS/TP53 initiated tumours in other contexts, there is little reason to believe that KRAS mutation can be 'lost' during tumour evolution (page 10) as RAS pathway addiction is a key driver in these tumours. The authors findings of 2/12 patients suggests that previous efforts in mesothelioma may have simply underestimated the frequency of these mutations, perhaps because of issues in depth of sequencing and purity of mesothelioma samples.
• The view of cancer proposed in Figure 9H is relatively controversial as it sees tumours as communities of single mutant cells, rather than accumulating genetic damage through somatic evolution of cells with a common ancestor containing driver mutations. As above, I think technical issues rather than underlying biology are a more likely cause for the lack of appreciation of KRAS mutations in mesothelioma to date.

Typos:
• The ethics approval number is missing from page 14.
• Page 4: One mention of KRAS is formatted as Kras here.

RESPONSE TO EDITOR'S COMMENTS

C1. *"Thank you for the submission of your manuscript to EMBO Molecular Medicine. We have now received feedback from the three reviewers who agreed to evaluate your manuscript. As you will see from the reports below, the referees acknowledge the interest of the study and are overall supporting publication of your work pending appropriate revisions. Addressing the reviewers' concerns in full will be necessary for further considering the manuscript in our journal, and in particular, the implications of your findings for human mesothelioma should be further clarified (but not overstated). Acceptance of the manuscript will entail a second round of review. EMBO Molecular Medicine encourages a single round of revision only and therefore, acceptance or rejection of the manuscript will depend on the completeness of your responses included in the next, final version of the manuscript. For this reason, and to save you from any frustrations in the end, I would strongly advise against returning an incomplete revision".*

R1. We cordially thank the Editor for the positive appraisal of our manuscript. We undertook every effort feasible to address the comments put forth, as detailed in the Letter to the Editor and in this Response to the Comments of the Reviewers. In addition, we formatted the manuscript according to *EMBO Mol Med* guidelines and now provide all source data.

C2. *"When submitting your revised manuscript, please carefully review the instructions that follow below. Failure to include requested items will delay the evaluation of your revision: 1) A .docx formatted version of the manuscript text (including legends for main figures, EV figures and tables). Please make sure that the changes are highlighted to be clearly visible. 2) Individual production quality figure files as .eps, .tif, .jpg (one file per figure). 3) A .docx formatted letter INCLUDING the reviewers' reports and your detailed point-by-point responses to their comments. As part of the EMBO Press transparent editorial process, the point-by-point response is part of the Review Process File (RPF), which will be published alongside your paper. 4) A complete author checklist, which you can download from our author guidelines (https://www.embopress.org/page/journal/17574684/authorguide#submissionofrevisions). Please insert information in the checklist that is also reflected in the manuscript. The completed author checklist will also be part of the RPF. 5) Before submitting your revision, primary datasets produced in this study need to be deposited in an appropriate public database (see https://www.embopress.org/page/journal/17574684/authorguide#dataavailability). Please remember to provide a reviewer password if the datasets are not yet public. The accession numbers and database should be listed in a formal "Data Availability" section (placed after Materials & Method). Please note that the Data Availability Section is restricted to new primary data that are part of this study. *** Note - All links should resolve to a page where the data can be accessed. *** 6) We would also encourage you to include the source data for figure panels that show essential data. Numerical data should be provided as individual .xls or .csv files (including a tab describing the data). For blots or microscopy, uncropped images should be submitted (using a zip archive if multiple images need to be supplied for one panel). Additional information on source data and instruction on how to label the files are available at https://www.embopress.org/page/journal/17574684/authorguide#sourcedata. 7) Our journal encourages inclusion of *data citations in the reference list* to directly cite datasets that were re-used and obtained from public databases. Data citations in the article text are distinct from normal bibliographical citations and should directly link to the database records from which the data can be accessed. In the main text, data citations are formatted as follows: "Data ref: Smith et al, 2001" or "Data ref: NCBI Sequence Read Archive PRJNA342805, 2017". In the Reference list, data citations must be labeled with "[DATASET]". A data reference must provide the database name, accession number/identifiers and a resolvable link to the landing page from*

*which the data can be accessed at the end of the reference. Further instructions are available at https://www.embopress.org/page/journal/17574684/authorguide#referencesformat. 8) We replaced Supplementary Information with Expanded View (EV) Figures and Tables that are collapsible/expandable online. A maximum of 5 EV Figures can be typeset. EV Figures should be cited as 'Figure EV1, Figure EV2" etc... in the text and their respective legends should be included in the main text after the legends of regular figures. - For the figures that you do NOT wish to display as Expanded View figures, they should be bundled together with their legends in a single PDF file called *Appendix*, which should start with a short Table of Content. Appendix figures should be referred to in the main text as: "Appendix Figure S1, Appendix Figure S2" etc. - Additional Tables/Datasets should be labeled and referred to as Table EV1, Dataset EV1, etc. Legends have to be provided in a separate tab in case of .xls files. Alternatively, the legend can be supplied as a separate text file (README) and zipped together with the Table/Dataset file. See detailed instructions here: https://www.embopress.org/page/journal/17574684/authorguide#expandedview. 9) The paper explained: EMBO Molecular Medicine articles are accompanied by a summary of the articles to emphasize the major findings in the paper and their medical implications for the non-specialist reader. Please provide a draft summary of your article highlighting - the medical issue you are addressing, - the results obtained and - their clinical impact. This may be edited to ensure that readers understand the significance and context of the research. Please refer to any of our published articles for an example. 10) For more information: There is space at the end of each article to list relevant web links for further consultation by our readers. Could you identify some relevant ones and provide such information as well? Some examples are patient associations, relevant databases, OMIM/proteins/genes links, author's websites, etc... 11) Every published paper now includes a 'Synopsis' to further enhance discoverability. Synopses are displayed on the journal webpage and are freely accessible to all readers. They include a short stand first (maximum of 300 characters, including space) as well as 2-5 one-sentence bullet points that summarizes the paper. Please write the bullet points to summarize the key NEW findings. They should be designed to be complementary to the abstract - i.e. not repeat the same text. We encourage inclusion of key acronyms and quantitative information (maximum of 30 words / bullet point). Please use the passive voice. Please attach these in a separate file or send them by email, we will incorporate them accordingly. Please also suggest a striking image or visual abstract to illustrate your article. If you do please provide a png file 550 px-wide x 400-px high. 12) As part of the EMBO Publications transparent editorial process initiative (see our Editorial at http://embomolmed.embopress.org/content/2/9/329), EMBO Molecular Medicine will publish online a Review Process File (RPF) to accompany accepted manuscripts. In the event of acceptance, this file will be published in conjunction with your paper and will include the anonymous referee reports, your point-by-point response and all pertinent correspondence relating to the manuscript. Let us know whether you agree with the publication of the RPF and as here, if you want to remove or not any figures from it prior to publication. Please note that the Authors checklist will be published at the end of the RPF. EMBO Molecular Medicine has a "scooping protection" policy, whereby similar findings that are published by others during review or revision are not a criterion for rejection. Should you decide to submit a revised version, I do ask that you get in touch after three months if you have not completed it, to update us on the status."*

R2. We thank the Editor for the detailed instructions for revision. We implemented all changes requested to conform to Journal style and provide now all the additional items required, including a .docx formatted manuscript with highlighted changes, individual production quality figures, a .docx formatted letter with reviewers' reports and a detailed point-by-point response to their comments, a complete author checklist, data deposition statements and reviewer password (included in this letter), a link to the page where the data can be accessed, source data files for

all figures, all uncropped gels in an Appendix Figure, data citations in the reference list, Expanded View Figures, Appendix with Table of Contents, The paper explained, For more information, and Synopsis sections, a striking image, as well as an agreement with the publication of the RPF and instructions to remove the data figures from it.

RESPONSE TO REVIEWERS' COMMENTS

Referee #1

C3. *"In this work investigators look at KRAS alterations as potential initiating event for malignant mesothelioma. They look at KRAS alterations in a series of mesothelioma samples and find a minority have mutations or amplification. Using an AdCRE system they deliver the KRAS alteration to the pleural cavity of mice and pleural changes resembling mesothelioma develop over many months. By altering p53 as well, mice develop a more biphasic histology. BAP1 alterations occur secondarily. They postulate the reason for the low KRAS mutations seen in clinical specimens is low sensitivity of the assays and regression of clones that are KRAS altered over time in favor of the clones. This reviewer agrees that a subset of mesotheliomas probably exist that maybe KRAS driven."*

R3. We cordially thank this Reviewer for the positive appraisal of our manuscript and its central idea. We undertook every effort feasible to address the comments (C; italic fonts) put forth, as detailed by our responses (R; regular fonts) below. All references refer to full references within the manuscript.

C4. *"In terms of the clinical specimens pathway analyses using RNA seq to determine which pathways are activated may be helpful. For example in the KRAS mutated are the relevant pathways showing activation. This data should be available from expression values. Are the KRAS mutated mesotheliomas showing activation of the pathway. As they are mostly seen in epithelioid variety does this effect prognosis. More evaluation of evaluation of clinical specimens would be helpful."*

R4. We thank the Reviewer for this pertinent comment. To address this, we undertook detailed analyses of the largest genomic and transcriptomic dataset of malignant pleural mesothelioma by the TCGA (new Figures 1 and 2). We analyzed mutation, copy number alteration, as well as clinical and gene expression data. Indeed, the KRAS pathway emanates as one of the most upregulated pathways in this important dataset. Detailed molecular and clinical analyses are now provided, which show that KRAS-altered mesotheliomas comprise approximately 20% of all mesotheliomas in the TCGA dataset and that they are a highly genome-altered and aneuploidic subset of patients that faces worse survival. Pathway analyses were also performed using PANTHER db (http://www.pantherdb.org/) that show enrichment of the KRAS pathway in human mesothelioma overall. The new information is provided in the new Figures 1 and 2 and the relevant text of the revised manuscript.

C5. *"Although they report copy number alteration the actual copy number values is required. Many of the alterations seem low copy number which diminish their clinical value. What are the co-mutations in the KRAS altered mesotheliomas as opposed to other mesotheliomas."*

R5. We thank the Reviewer for this comment that helped us improve the paper. We analyzed copy number in the TCGA and our cohorts and report detailed and very interesting results including actual copy numbers in the new figures 1-3. The low copy numbers of KRAS mutations are

also summarized in new Figure 3H and makes our hypothesis of these mutations getting lost in NGS plausible. We show in our cohort how *KRAS* allelic frequency is lower than that of other oncogenes. This is also evident from another unpublished dataset, where we applied ddPCR for KRAS codon 12 and 13 and for EGFR exon 19 to 200 lung adenocarcinomas. The ddPCR findings here were validated by clinical grade sequencing in all cases! In this tumor type, where KRAS is a known driver in 30% of the cases that occur in Caucasians, KRAS copy numbers (median = 19.9%) were also statistically significantly lower than EGFR copy numbers (median = 33.0 %) (Figure on the right; *P*, probability; Mann-Whitney test; n = 56 KRAS-mutant and n = 19 EGFR-mutant patients out of n = 200; mutation frequencies = 28% KRAS and 10% EGFR). These data validate our ddPCR approach in MPM and is hopefully convincing this Reviewer that our findings are true and important. In this lung adenocarcinoma cohort, KRAS mutations are also extremely low in some patients, but KRAS mutations still drive their cancers.

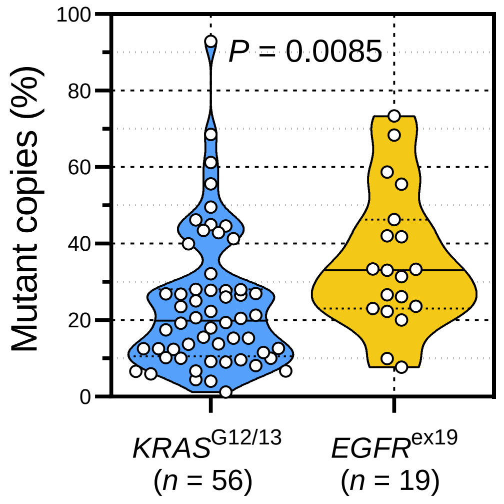

The low copy number of KRAS mutations observed in lung adenocarcinoma is even lower in mesothelioma, as we show for the first time, and this effect can be explained by the heterozygous nature of KRAS mutations as well as the intense inflammatory response these tumors cause (Agalioti et al, 2017; Marazioti et al, 2018), which would further dilute out the mutant copy signal in next generation sequencing approaches. We classified patients as KRAS-altered when they had a mutation, amplification, chromosome 12p gain, or overexpression and actually found 17 such patients. Similarly, we classified patients as TP53-altered when they had a mutation, deletion, chromosome 17p loss, or over- or under-expression and actually found 36 such patients, 7 of which also had a KRAS alteration. These KRAS-altered patients had TP53-alterations as expected by chance, but tended to be mutually exclusive with NF2 mutations (enrichment -1.98-fold; P = 0.1774). This is very interesting because NF2 is a KRAS inhibitor (Tikoo et al, 1994). The data are shown in new Figure 2E. The full co-mutation table follows below:

KRAS-normal	# Mutated patients	Profiled Samples	KRAS-altered	# Mutated patients	Profiled Samples	Enriched in	Fold-enrichment	Probability (hypergeometric test)
NF2	18	69	NF2	2	17	KRAS-normal	1.12	0.1774
BAP1	14	69	BAP1	4	17	KRAS-altered	1.12	0.4991
TP53	11	69	TP53	3	17	KRAS-altered	1.08	0.557
TTN	9	69	TTN	3	17	KRAS-altered	1.26	0.4371
LATS2	7	69	LATS2	1	17	KRAS-normal	1.09	0.5033
SETD2	7	69	SETD2	1	17	KRAS-normal	1.09	0.5033

C6. *“If in fact KRAS alterations are in only a sub clone of specimens (and this number is very low) which requires highly sensitive techniques and increased depth of coverage how can this be clinically important.”*

R6. We thank the Reviewer for this point. We show now evidence of *KRAS* alterations in 20% of TCGA patients and in 45% of our patients most of which would go undetected by conventional methods. We show also in our cohorts how *KRAS* allelic frequency is lower than that of other oncogenes. This can be explained by the heterozygous nature of KRAS mutations as well as the intense inflammatory response these tumors cause (Agalioti et al, 2017; Marazioti et al, 2018), which would further dilute out the mutant copy signal in next

generation sequencing approaches. We believe that KRAS mutations are important even at a lower allelic frequency as shown in mouse models, since they can drive cancer stemness and tumor progression (Li et al, 2020). This can be clinically important because this would mean that even these MPM with low allelic frequency would be refractory to other treatments and would respond to KRAS blockade. Based on our findings, we propose sensitive targeted methods to diagnose KRAS mutations in mesothelioma, in order to identify these patients and potentially treat them. This point of the Reviewer is now discussed in the revised discussion.

C7. *"The discussion should be around discussing a novel subgroup of mesotheliomas that maybe KRAS driven (like lung cancer) and we should try to identify these patients. Most malignancies are being subgroup by genomic alterations and subgroups being targeted for therapeutic vulnerabilities. Therefore a hierarchical clustering of mesotheliomas that are KRAS driven or not should be performed (from the clinical specimens already out there in the literature)."*

R7. We thank the Reviewer for this comment. We performed all of the requested analyses and show now the clinical and molecular profile as well as the hierarchical clustering according to the genes overexpressed by these tumors. We rewrote the paper to better characterize this molecular subclass of KRAS-driven MPM and also revised the discussion to focus on this subclass rather than talking about mesothelioma in general.

C8. *"The over emphasis on the presence of effusions in mesothelioma is wrong. Many mesotheliomas do not have effusions and this is not a diagnostic criteria."*

R8. We thank the Reviewer for this important point and we de-escalated the emphasis on effusion in the revised manuscript. However, we think that effusions make mesotheliomas more aggressive and thus play a biological role. This has been shown for non-small-cell lung cancer (Ryu et al, 2014), but unfortunately clinical cohorts of mesothelioma are small and underpowered to detect this effect. To accommodate the Reviewer's comment, we rewrote all the relevant sections to downplay the importance of effusions in our models, we just mentioned that the combined KRAS-TP53 model also features effusions, which is important since some patients with MPM also have effusions.

Referee #2

C9. *"The human data are highly hypothetical, not supported by the data. The information in the literature relating RAS alterations to mesothelioma: There is an extreme paucity of publications supporting the relevance of somatic RAS mutations in human mesothelioma. A few papers report RAS pathway activation predominantly in cell lines or mouse data. Recently a Nature Communication paper reported the involvement of RAS in causing pleural effusions in mice, the study is based on the use of cell lines and combines data from adenocarcinoma and mesothelioma together. Unfortunately that study includes only one MPM murine cell line as a representative of the MPM tumor type. Large sequencing studies including Bueno et al, did not find a significant amount of mutations in the RAS pathway, however KRAS pathway up-regulation was identified by mRNA analysis in some specimens. In this paper I am reviewing, the authors utilize a very sensitive technique, the digital droplet PCR to detect mutations in a small fraction of tumor cells to detect alterations in KRAS in human pleural mesothelioma samples. In a cohort of 12 mesothelioma samples they found 1 sample with a frequency of RAS mutation < 10% and 2 with frequency <1%. While this new technique has proven to be sensitive and specific (30504843, 31477768) to detect mutations in cell subpopulations, the number of samples analyzed here is too small to make conclusions about representation of RAS mutation in human MPM at large."*

R9. We cordially thank this Reviewer for the positive appraisal of our manuscript and its central idea. We also thank this Reviewer for mentioning our previous work on malignant pleural effusions (Agalioti et al, 2017; Marazioti et al, 2018). We undertook every effort feasible to address the comments (C; italic fonts) put forth, as detailed by our responses (R; regular fonts) below. All references refer to full references within the manuscript. We have now included important new human data in the revised manuscript and hope to alleviate the Reviewer's concern. We analyzed the largest human molecular MPM cohort (TCGA) and report now 20% mutation frequency of *KRAS* with or without *TP53* alterations using specific diagnostic criteria (Smith & Sheltzer, 2018). In more detail, we analyzed TCGA mutation, copy number alteration, as well as clinical and gene expression data. Indeed, the KRAS pathway emanates as one of the most upregulated pathways in this important dataset. Detailed molecular and clinical analyses are now provided, which show that KRAS-altered mesotheliomas comprise approximately 20% of all mesotheliomas in the TCGA dataset and that they are a highly genome-altered and aneuploidic subset of patients that faces worse survival. Pathway analyses were also performed using PANTHER db (http://www.pantherdb.org/) that show enrichment of the KRAS pathway in human mesothelioma overall. We also enhanced the analyses in our cohorts and now report deep molecular data for 46 patients with MPM and evidence for KRAS alteration in 45% of our patients. It is very difficult for us to attain a larger sample size as MPM is rather infrequent, but we are content to have enrolled 46 patients from three countries (more than 50% of TCGA enrollment). The new information is provided in the new Figures 1-3 and the relevant text of the revised manuscript. To accommodate the Reviewer's concern, we include her/his skepticism in the final sentence of the revised discussion.

C10. *"The authors then analyze 32 patient-derived MPM cell lines by CytoScan, a SNP-based sensitive technique to detect CNV (the sensitivity of this technique should be similar to a high density microarray). Although this dataset shows that the technique used is sensitive, the use of cell lines limits the relevance of these findings, as it is well known that cell lines accumulate genetic damage in tissue culture that is not representative of the initial genetic damage that caused tumor growth. Primary mesothelioma biopsies should have been analyzed by CytoScan to support the cell line results. A characterization of patient characteristics and the cellular content of the malignant pleural effusion used to generate the data with ddPCR and CytoScan should also be included."*

R10. We agree with the Reviewer that analysis of primary samples would be preferential. Unfortunately, this was impossible for us since these samples do not exist anymore. To address this point, we performed additional analyses on the cell lines and present now single nucleotide variant in addition to copy number variation data, with impressive results, since more KRAS alterations were found, although the cell lines have a MPM-like mutation profile, now included in the source data of the study. Our assumption that these cell lines accurately represent the patient they were derived from relies on the fact that they were passaged less than 30 times after puncture and on evidence that emanates from authoritative work of others (Pauli et al, 2017), but also from our own hands (Kanellakis et al, 2020; Kanellakis et al, 2019), indicating that such cell lines can still reflect the initiating oncogenic events, that acquired genetic damage in culture is minimal compared with the in vivo mutation burden of these tumors, and they still present valuable tools. We also included now all available clinical data from the German and French patients in Appendix Tables S1 and S2 and provide the raw data in the respective source data files.

C11. *"To support their findings, the authors analyze previously published WES data sets from human mesothelioma samples (fig. 1d) from refs 13-21 and conclude that about 10% of the*

samples carried RAS mutations. This finding is potentially interesting especially because it was not noted or described by the authors of those studies, did they really miss it?. Given that the Authors of this paper I am reviewing have an "additional" interpretation of the data presented in refs 13-21, they authors should include a summary table describing all the RAS mutations found in each study, the frequency in each dataset, the total number of samples per dataset, the specific type of mutations, gene (chromosome/cDNA location) and their putative pathogenicity score. If these mutations were not listed in the previous publications (main paper or supplementary data), the authors should also include a description of the method used to analyze the raw data and mention the databases used to access those data. In other words they need to explain us how and why they re-interpreted these data to conclude that pathogenic RAS mutations are relatively frequent."

R11. We apologize for not describing the curation method of this dataset, which was corrected in this revision. The raw data are also provided in the respective source data file. In more detail, the aforementioned analysis consisted of manual curation of the main and supplementary data of cited studies (Bott et al, 2011; Enomoto et al, 2012; Mezzapelle et al, 2013; Shukuya et al; 2014; Guo et al, 2015; Lo Iacono et al, 2015; Bueno et al, 2016; De Rienzo et al, 2016; Kato et al, 2016; Hmeljak et al, 2018). The last cited study (Hmeljak et al, 2018) represents the TCGA dataset and was analyzed in detail in New Figures 1 and 2 since the data are publicly available.

C12. *"The mouse data presented are instead convincing. The authors demonstrated that RAS alone and more potently in combination with TP53 mutations promote mesothelioma in mice. To see if the mouse data are relevant to human mesothelioma, the authors should investigate whether concurrent TP53 and KRAS mutations are present in human mesotheliomas (ddPCR can be easily multiplexed as published in 30504843). The authors should also use the previously published data they refer to (refs.13-21 shown in fig 1d) and maybe the TGCA dataset to find out if there is any overlap between TP53 mutations and RAS mutations in clinical mesothelioma samples."*

R12. We cordially thank this Reviewer for the positive appraisal of our mechanistic studies. We performed additional ddPCR for TP53 copy number in our patients and indeed found 2 of 12 MPMs with dual KRAS and TP53 alterations. As discussed above, we also thoroughly analyzed the TCGA dataset and discovered 7 out of 86 MPM patients with dual KRAS and TP53 alterations.

C13. *"The conclusive hypothesis drawn by the authors that "Mesothelial KRAS and other RAS pathway mutations may initiate MPM in some patients, but may be subsequently lost during tumor evolution, or may persist at a clonal level going undetected during sampling/sequencing, as has been shown for lung tumours51,52" and depicted in fig.9h, is highly hypothetical as it is not substantiated by the data presented. Presently these data show that RAs mutations together with TP53 mutations cause mesothelioma in mice. The human data are too preliminary to be presented."*

R13. We agree with this Reviewer and apologize for over-interpreting our results in the initial submission. We now undertook every effort feasible to strengthen our human data on the one hand and to dampen their interpretation. As a result, we hope to satisfy the constructive criticism of this Reviewer. We also removed the schematic to prevent over- or mis-interpretation of the data.

C14. *"Page 6 line 4 from bottom. Fig. reference: should be Fig. 3a,b and not 2,a b."*

R14. We thank this Reviewer, apologize for the error, and corrected as appropriate.

C15. *"Some of the literature cited about mesothelioma is old and does not reflect current knowledge, for example reference 1-2 used as general reference for the epidemiology of mesothelioma, references 9-12 used for "predictive factors of survival". Rather use recent reviews published in 2019 and 2020 in some of the top high impact factor medical and scientific journals."*

R15. We thank this Reviewer, apologize for the outdated references, and corrected with recent state-of-the-art literature in the field, as appropriate.

Referee #3

C16. *"The authors perform characterization of patient* mesothelioma samples derived from pleural *effusions, finding that 2 of 12 patients have a KRAS oncogenic driver mutation and in as separate cohort of cell lines that copy number events at this locus are also frequent. They then use a KRas/Trp53 mutant mouse model to demonstrate the formation of mesothelioma when those genes are altered in pleura. The manuscript is a significant step forward in mouse modelling of mesothelioma, is well-written and I have only relatively minor suggestions for improvements."*

R16. We cordially thank this Reviewer for the positive appraisal of our manuscript and its central idea. We undertook every effort feasible to address the comments (C; italic fonts) put forth, as detailed by our responses (R; regular fonts) below. All references refer to full references within the manuscript.

C17. *"The authors could provide more context for their findings by clearly stating at the outset what proportions of mesothelioma patients are thought to have KRAS, TP53, BAP1 etc. mutations based on prior large genomic sequencing studies (TCGA etc)."*

R17. We thank this Reviewer for this point. We applied it to new Figure 1B and in the relevant text and clarify what is thought and what our study brings to the field.

C18. *"The claim in the abstract that "a significant proportion of human MPM harbor point mutations and copy number alterations in the KRAS proto-oncogene" might require validation in larger cohorts."*

R18. We totally agree with this Reviewer and apologize for the interpretation of the data. To address this point, we undertook detailed analyses of the largest genomic and transcriptomic dataset of malignant pleural mesothelioma by the TCGA (new Figures 1 and 2). We analyzed mutation, copy number alteration, as well as clinical and gene expression data. Indeed, the KRAS pathway emanates as one of the most upregulated pathways in this important dataset. Detailed molecular and clinical analyses are now provided, which show that KRAS-altered mesotheliomas comprise approximately 20% of all mesotheliomas in the TCGA dataset and that they are a highly genome-altered and aneuploidic subset of patients that faces worse survival. Pathway analyses were also performed using PANTHER db (http://www.pantherdb.org/) that show enrichment of the KRAS pathway in human mesothelioma overall. The new information is provided in the new Figures 1 and 2 and the relevant text of the revised manuscript. We also reanalyzed and expanded our own cohort studies and present now new evidence that supports that an even larger proportion of our patients (up to 45% of 46 patients) harbor KRAS alterations. To accommodate this point, we

also re-wrote the abstract and the whole text to avoid over-interpretation of our findings and we state the Reviewer's point in the discussion.

C19. *"Why are a different patient cohort used for the ddPCR and Affymetrix CytoScanHD assays? What are the KRAS mutation statuses of the cell lines?"*

R19. These are different patients from Germany and France, which were recruited in different studies and were subjected to different assays. They are both reported together in this study, in order to increase sample size of a relatively rare disease. This was achieved and we present n = 46 now, which is a significant patient number since TCGA achieved n = 86 (Hmeljak et al, 2018) and Bueno et al, 2016 achieved n = 52. To accommodate the Reviewer's concern, we expanded and harmonized the assays done in the two studies and now present both single nucleotide variant and copy number variation data for both French and German patients. The new data are shown in new Figure 3 and the associated text and source data.

C20. *"Additional markers such as mesothelin, WT1 and vimentin could be added to the characterization in Figure 6 for a more comprehensive assessment."*

R20. This was done, and all three new markers are now included in revised Figure 5.

C21. *"Figure 8D - do these mutations actually effect BAP1 function? In this panel what do blue vs red dots represent? Do the mutations affect BAP1 nuclear localization (this is more commonly used as a read out than sequencing in mesothelioma)."*

R21. We thank this Reviewer for this pertinent comment. In the lollipop plots of new Figure EV5, red is used for single nucleotide variation (SNV) while the blue for insertion causing frameshift. This is now clarified in the legend. The investigation on *Bap1* was conducted on three different levels, as was done in (Bueno et al, 2016): next generation sequencing (Fig EV5A), Sanger sequencing (Fig EV5B), and immunohistochemistry (Fig EV5C). Despite the several deleterious alterations that are identified by sequencing, we show that BAP1 protein in still translated and detectable in the nucleus of our *KRAS*-mutant murine mesotheliomas. This nuclear localization of mutant *Bap1* is now pointed out in new Figure EV5C with arrows and text.

C22. *"Figure 8A is unreadably small as presented."*

R22. We thank this Reviewer, apologize for the error, and corrected as appropriate. The enhanced Figure is presented as new Fig EV5.

C23. *"Based on KRAS/TP53 initiated tumors in other contexts, there is little reason to believe that KRAS mutation can be 'lost' during tumor evolution (page 10) as RAS pathway addiction is a key driver in these tumors. The authors findings of 2/12 patients suggests that previous efforts in mesothelioma may have simply underestimated the frequency of these mutations, perhaps because of issues in depth of sequencing and purity of mesothelioma samples. The view of cancer proposed in Figure 9H is relatively controversial as it sees tumors as communities of single mutant cells, rather than accumulating genetic damage through somatic evolution of cells with a common ancestor containing driver mutations. As above, I think technical issues rather than underlying biology are a more likely cause for the lack of appreciation of KRAS mutations in mesothelioma to date.*

R23. We thank this Reviewer for this pertinent comment. We agree, we re-wrote the paper removing this speculation, we removed the graphic in question, and we systematically assessed KRAS alterations in the TCGA and our own cohorts to better define the molecular subclass of KRAS-altered mesotheliomas.

C24. *"The ethics approval number is missing from page 14."*

R24. We thank this Reviewer, apologize for the unfortunate error, and corrected as appropriate. All ethics information is now included in the revised manuscript.

C25. *"Page 4: One mention of KRAS is formatted as Kras here."*

R25. We use *Kras* for the mouse allele and *KRAS* for the human. We refer to the mouse allele in this instance, and this is now clarified from the start in the relevant section.

Dear Dr. Stathopoulos,

Thank you for submitting your revised manuscript to EMBO Molecular Medicine. We have now heard back from the three referees who re-evaluated your manuscript.

As you will see from the reports pasted below, the referees acknowledge the efforts that were made to address their concerns. However, referee #2 in particular still raises a major issue regarding the lack of validation in patients' data.
This issue was further discussed with the other referees, and referee #3 commented:
"I must say I share some of the concerns of reviewer 2 regarding the re-analysis of human data. Anything the authors can do to improve this analysis would improve the paper, particularly if there are independent WGS/WES sequences available through collaboration. Simply establishing if low frequency KRAS mutations are present in such datasets would be achievable on a reasonable timeframe.
I suspect the answer lies in the low allelic frequency of these mutations. Previous authors might have missed these below thresholds for mutation calling. It's possible that when the current authors have gone back and looked specifically at this locus, there are in fact some mutant reads present. Better clarity on how their methods differ to prior analyses and why the results were not found by others would be useful in any case."

In some cases, we may consult with the authors before deciding on the outcome. In this case, we would like to know if you would be ready to perform additional experiments and further modify your manuscript to address this particular concern.
EMBO Press usually allows one round of major revisions only, but here we would exceptionally invite a second round of revisions as the three referees acknowledge the interest of your findings (as we do).

We understand that revising the manuscript according to the referees' recommendations would require additional work. Therefore, should you find that the requested revisions are not feasible and prefer, therefore, to submit your paper elsewhere, we would welcome a message to this effect. Alternatively, should you be willing to undertake a second round of revisions, please send me a preliminary point-by-point rebuttal letter that I would then share with the referees before reaching a decision.

Looking forward to hearing from you,

With kind regards,

Lise Roth

Lise Roth, PhD
Editor
EMBO Molecular Medicine

Referee #1:
The authors have extensively revised their manuscript in my view have adequately answered the reviewers questions.

Referee #2:
The model should be independently validated.

The authors have done a great job in trying to politely address criticisms. The problem remains, however, that they are finding RAS mutations that others who analyzed the same specimens did not. The authors write (R10) in their rebuttal: We agree with the Reviewer that analysis of primary samples would be preferential. Unfortunately, this was impossible for us since these samples do not exist anymore. So they use cell lines, but this will not convince most readers. In short, the papers reads well, but I am sure t that this paper would have a much greater impact if independently validated. There are plenty of primary specimens to be used for these analyses: the authors list in the references most of the physician scientists who have these specimens: for example Bueno, De Perrot, Fennel, Hassan, Pass, and many others. All of them have provided their cohort for validation multiple times to others in the mesothelioma research field. Many of them already have done WGS, WES and RNAseq on the, so should be pretty easy to check and validate. Same problem with R11: why the authors of the original papers References 13-21- failed to identify these mutations? I guess the answer is because their biostatisticians were not as good as those who co-authored this paper, but it is a little hard to believe that so many people could have overlooked these mutations. Of course it is possible. I am not a biostatistician so I cannot express an opinion, but I hope the authors understand why I am concerned. In short either these data are reproducible -and everybody else had missed so far the relevance of the RAS pathway to mesothelioma, and then the data are important because they provide a novel target for therapy, or the data are not reproducible, if so, it would be better to know this before not after publication.

Referee #3:
Marazioti et al. have revised their manuscript thoroughly based on the advice of reviewers. The human data remain the least certain but these data are less strongly interpreted in the revised manuscript and methods are now

presented that will allow readers to compare this study with previous analyses of the same data. I support publication of the manuscript, including the human data, which as the authors point out, do add substantially to the available data for MPM.

• Regarding the low allelic frequency of KRAS mutations noted in the study, I wondered if the authors could add an analysis of whole-genome doubling. It occurred to me that wGD might be enriched in the KRAS/TP53 altered patient cohort and that this might explain the low allelic frequency of KRAS. Further, if wGD precedes KRAS mutation then the presence of more KRAS alleles might make KRAS mutation acquisition more likely.

• How did the authors select the one MAPED trial patient for investigation?This is important information to include given the patient turned out to have 4 KRAS mutations and TP53 alteration.

• The immunohistochemistry performed for BAP1 does not "validate" (p. 12) the BAP1 mutations found using sequencing approaches since it shows that BAP1 protein remains present and in the expected location in these cell lines. In MPM, it is thought that loss of BAP1 nuclear protein correlates with functionally significant BAP1 mutations (Nasu et al., 2015, JTO). This should be re-worded in the results text.

HelmholtzZentrum münchen
German Research Center for Environmental Health

CPC Comprehensive Pneumology Center

Helmholtz Zentrum München ·P.O. Box 11 29 ·85758 Neuherberg

Dr. Georgios T. Stathopoulos, MD PhD
Group Leader
Lung Carcinogenesis

Comprehensive Pneumology Center
T +49(0)89 3187-4846
F +49(0)89 3187-4661
stathopoulos@helmholtz-muenchen.de

TO: Dr. Lise Roth, Editor, *EMBO Mol Med*
RE: "KRAS signalling in malignant pleural mesothelioma", Marazioti et al., EMM-2020-13631.R2

Munich, Germany,
23. April 2021

Resubmission of revised manuscript EMM-2020-13631.R2

Dear Dr. Roth,

Dear Editor,

We are very sorry that we did not persuade all three reviewers about the validity and importance of our findings. In essence, the reviewers want us to reanalyze "independent WGS/WES sequences available through collaboration".

At the same time, we are thankful to you and all three reviewers for the positive appraisal of our work and the comments provided, that will help us improve the paper further. We hereby commit to resubmit a revised manuscript referenced above in due time.

We will obtain significant new data from published datasets and we will test additional 23 MPM patients from Turkey reaching a total *n* = 69). To this end, we will contact every possible authority to obtained raw or analyzed data to corroborate our findings, including the physician scientists mentioned by Referee #2 (Drs. Bueno, De Perrot, Fennel, Hassan, Pass). We already heard back from Dr. Fennel and we have his commitment to contribute the data essential for this revision.

We will undertake every effort feasible to address all editorial and reviewer's comments in an extensive and full revision. A detailed response to the reviewers' concerns will be as follows below this letter.

With best regards and hope for your appreciation of our efforts and your positive response,

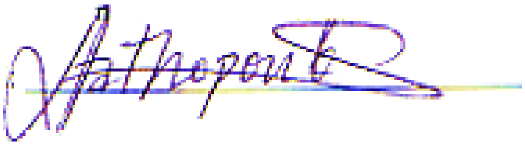

Georgios T. Stathopoulos, MD PhD

Helmholtz Zentrum München
Deutsches Forschungszentrum für
Gesundheit und Umwelt (GmbH)
Ingolstädter Landstr. 1
85764 Neuherberg
T +49(0)89 3187 (0)
F +49(0)89 3187 3322

info@helmholtz-muenchen.de
www.helmholtz-muenchen.de
Aufsichtsratsvorsitzende:
Petra Steiner-Hoffmann

Geschäftsführer:
Prof. Dr. med. Dr. h.c. Matthias H. Tschöp
Kerstin Günther

Registergericht:
Amtsgericht München HRB 6466
USt-IdNr- DE 129521671

Bankverbindung:
Münchner Bank eG
Konto-Nr. 2 158 620
BLZ 701 900 00
IBAN DE04701900000002158620
BIC GENODEF1M01

RESPONSE TO REVIEWERS' COMMENTS

Referee #1

C1. *Referee #1:*
The authors have extensively revised their manuscript in my view have adequately answered the reviewers questions.

We are extremely grateful to this reviewer for her/his appreciation of our work and effort spent in this revision.

Referee #2:

C2. *The model should be independently validated.The authors have done a great job in trying to politely address criticisms. The problem remains, however, that they are finding RAS mutations that others who analyzed the same specimens did not. The authors write (R10) in their rebuttal: We agree with the Reviewer that analysis of primary samples would be preferential. Unfortunately, this was impossible for us since these samples do not exist anymore. So they use cell lines, but this will not convince most readers. In short, the papers reads well, but I am sure t that this paper would have a much greater impact if independently validated. There are plenty of primary specimens to be used for these analyses: the authors list in the references most of the physician scientists who have these specimens: for example Bueno, De Perrot, Fennel, Hassan, Pass, and many others. All of them have provided their cohort for validation multiple times to others in the mesothelioma research field. Many of them already have done WGS, WES and RNAseq on the, so should be pretty easy to check and validate. Same problem with R11: why the authors of the original papers References 13-21- failed to identify these mutations? I guess the answer is because their biostatisticians were not as good as those who co-authored this paper, but it is a little hard to believe that so many people could have overlooked these mutations. Of course it is possible. I am not a biostatistician so I cannot express an opinion, but I hope the authors understand why I am concerned. In short either these data are reproducible -and everybody else had missed so far the relevance of the RAS pathway to mesothelioma, and then the data are important because they provide a novel target for therapy, or the data are not reproducible, if so, it would be better to know this before not after publication.*

We thank this reviewer for her/his appreciation of our effort and her/his honest critiques. To address them, we commit to obtaining significant new data from independent published datasets and to testing additional 23 MPM patients from Turkey reaching a total n = 69). To this end, we will contact every possible authority to obtained raw or analyzed data to corroborate our findings, including the physician scientists mentioned by Referee #2 (Drs. Bueno, De Perrot, Fennel, Hassan, Pass).

Referee #3:

C3. *Marazioti et al. have revised their manuscript thoroughly based on the advice of reviewers. The human data remain the least certain but these data are less strongly interpreted in the revised manuscript and methods are now presented that will allow readers to compare this study with previous analyses of the same data. I support publication of the manuscript, including the human data, which as the authors point out, do add substantially to the available data for MPM.*

We are extremely grateful to this reviewer for her/his appreciation of our work and effort spent in this revision.

C4. *Regarding the low allelic frequency of KRAS mutations noted in the study, I wondered if the authors could add an analysis of whole-genome doubling. It occurred to me that wGD might be enriched in the KRAS/TP53 altered patient cohort and that this might explain the low allelic frequency of KRAS. Further, if wGD precedes KRAS mutation then the presence of more KRAS alleles might make KRAS mutation acquisition more likely.*

We are extremely grateful to this reviewer for her/his idea and commit to perform such analyses.

C5. *How did the authors select the one MAPED trial patient for investigation? This is important information to include given the patient turned out to have 4 KRAS mutations and TP53 alteration.*

The MAPED study was a clinical prospective observational trial that did not include sample biobanking. The single patient included was one single mesothelioma patient out of 15 others who contacted us independently and wished to donate samples for research. We will be happy to eliminate the patient data from the revised paper in the light of the new patients from Turkey, if the reviewer wishes so (of course, we would prefer to keep the data in the paper).

C6. *The immunohistochemistry performed for BAP1 does not "validate" (p. 12) the BAP1 mutations found using sequencing approaches since it shows that BAP1 protein remains present and in the expected location in these cell lines. In MPM, it is thought that loss of BAP1 nuclear protein correlates with functionally significant BAP1 mutations (Nasu et al., 2015, JTO). This should be re-worded in the results text.*

We apologize for our mistake and we will correct and reference as appropriate.

23rd Apr 2021

Dear Dr. Stathopoulos,

Thank you for the submission of your revised manuscript to EMBO Molecular Medicine. As discussed in a previous correspondence, while the referees appreciate the work that was made to address their concerns, they also raise issues that remain unanswered.
I therefore contacted you to ask whether you would be willing to exceptionally perform an additional round of revisions to address the remaining issues. You kindly provided a provisional point-by-point letter, and after discussion with my colleagues, we agree that the proposed revisions would likely address the referees' concerns.
Please be aware that this will be the last chance for you to address these points.

Additionally, please also address the following editorial issues:
- We can accommodate up to 5 keywords, please correct accordingly.
- Please upload your checklist as an excel file.
- Thank you for providing a Data Availability section, please move it after the Material and Methods section.
- Please make sure that the funding information provided in the submission system and in the manuscript match.
- Thank you for providing Source Data. Please upload them as 1 file per figure.
- Please make sure all figures are referenced in the main text.
- As part of the EMBO Publications transparent editorial process initiative (see our Editorial at http://embomolmed.embopress.org/content/2/9/329), EMBO Molecular Medicine will publish online a Review Process File (RPF) to accompany accepted manuscripts.
In the event of acceptance, this file will be published in conjunction with your paper and will include the anonymous referee reports, your point-by-point response and all pertinent correspondence relating to the manuscript. Let us know whether you agree with the publication of the RPF and as here, if you want to remove or not any figures from it prior to publication.
Please note that the Authors checklist will be published at the end of the RPF.

EMBO Molecular Medicine has a "scooping protection" policy, whereby similar findings that are published by others during review or revision are not a criterion for rejection. Should you decide to submit a revised version, I do ask that you get in touch after three months if you have not completed it, to update us on the status.

I look forward to receiving your revised manuscript.

With kind regards,

Lise Roth

Lise Roth, PhD
Editor
EMBO Molecular Medicine

***** Reviewer's comments *****

Referee #1 (Remarks for Author):

The authors have extensively revised their manuscript in my view have adequately answered the reviewers questions.

Referee #2 (Comments on Novelty/Model System for Author):

The model should be independently validated

Referee #2 (Remarks for Author):

The authors have done a great job in trying to politely address criticisms. The problem remains, however, that they are finding RAS mutations that others who analyzed the same specimens did not. The authors write (R10) in their rebuttal: We agree with the Reviewer that analysis of primary samples would be preferential. Unfortunately, this was impossible for us since these samples do not exist anymore. So they use cell lines, but this will not convince most readers. In short, the papers reads well, but I am sure t that this paper would have a much greater impact if independently validated. There are plenty of primary specimens to be used for these analyses: the authors list in the references most of the physician scientists who have these specimens: for example Bueno, De Perrot, Fennel, Hassan, Pass, and many others. All of them have provided their cohort for validation multiple times to others in the mesothelioma research field. Many of them already have done WGS, WES and RNAseq on the, so should be pretty easy to check and validate. Same problem with R11: why the authors of the original papers References 13-21- failed to identify these mutations? I guess the answer is because their biostatisticians were not as good as those who co-authored this paper, but it is a little hard to believe that so many people could have overlooked these mutations. Of course it is possible. I am not a biostatistician so I cannot express an opinion, but I hope the authors understand why I am concerned. In short either these data are reproducible -and everybody else had missed so far the relevance of the RAS pathway to mesothelioma, and then the data are important because they provide a novel target for therapy, or the data are not reproducible, if so, it would be better to know this before not after publication.

Referee #3 (Remarks for Author):

Marazioti et al. have revised their manuscript thoroughly based on the advice of reviewers. The human data remain the least certain but these data are less strongly interpreted in the revised manuscript and methods are now presented that will allow readers to compare this study with previous analyses of the same data. I support publication of the manuscript, including the human data, which as the authors point out, do add substantially to the available data for MPM.

• Regarding the low allelic frequency of KRAS mutations noted in the study, I wondered if the authors could add an analysis of whole-genome doubling. It occurred to me that wGD might be enriched in the KRAS/TP53 altered patient cohort and that this might explain the low allelic frequency of KRAS. Further, if wGD precedes KRAS mutation then the presence of more KRAS alleles might make KRAS mutation acquisition more likely.

• How did the authors select the one MAPED trial patient for investigation? This is important information to include given the patient turned out to have 4 KRAS mutations and TP53 alteration.

• The immunohistochemistry performed for BAP1 does not "validate" (p. 12) the BAP1 mutations found using sequencing approaches since it shows that BAP1 protein remains present and in the expected location in these cell lines. In MPM, it is thought that loss of BAP1 nuclear protein correlates with functionally significant BAP1 mutations (Nasu et al., 2015, JTO). This should be re-worded in the results text.

RESPONSE TO REVIEWERS' COMMENTS

Referee #1

C1. *Referee #1:*
The authors have extensively revised their manuscript in my view have adequately answered the reviewers questions.

We are extremely grateful to this reviewer for her/his appreciation of our work and effort spent in this revision.

Referee #2:

C2. *The model should be independently validated. The authors have done a great job in trying to politely address criticisms. The problem remains, however, that they are finding RAS mutations that others who analyzed the same specimens did not. The authors write (R10) in their rebuttal: We agree with the Reviewer that analysis of primary samples would be preferential. Unfortunately, this was impossible for us since these samples do not exist anymore. So they use cell lines, but this will not convince most readers. In short, the papers reads well, but I am sure t that this paper would have a much greater impact if independently validated. There are plenty of primary specimens to be used for these analyses: the authors list in the references most of the physician scientists who have these specimens: for example Bueno, De Perrot, Fennel, Hassan, Pass, and many others. All of them have provided their cohort for validation multiple times to others in the mesothelioma research field. Many of them already have done WGS, WES and RNAseq on the, so should be pretty easy to check and validate. Same problem with R11: why the authors of the original papers References 13-21- failed to identify these mutations? I guess the answer is because their biostatisticians were not as good as those who co-authored this paper, but it is a little hard to believe that so many people could have overlooked these mutations. Of course it is possible. I am not a biostatistician so I cannot express an opinion, but I hope the authors understand why I am concerned. In short either these data are reproducible -and everybody else had missed so far the relevance of the RAS pathway to mesothelioma, and then the data are important because they provide a novel target for therapy, or the data are not reproducible, if so, it would be better to know this before not after publication.*

We thank this reviewer for her/his appreciation of our effort and her/his honest critiques. To address them, we obtained significant new data from independent cohorts and tested additional 23 MPM patients from France and Turkey reaching a total $n = 35$ human primary MPM samples and $n = 33$ human primary MPM cell lines. To this end, we contacted every possible authority to obtain data and samples to corroborate our findings, including all physician scientists mentioned by this Referee (Drs. Bueno, De Perrot, Fennel, Hassan, and Pass), in addition to our own contacts. Fortunately, we were able to secure a significant number of new primary MPM patient samples. The new data provided validate the presence of *KRAS* mutations in additional patients from France and Turkey, with or without *TP53* alterations, as shown in new Figure 4.

Referee #3:

C3. *Marazioti et al. have revised their manuscript thoroughly based on the advice of reviewers. The human data remain the least certain but these data are less strongly interpreted in the*

revised manuscript and methods are now presented that will allow readers to compare this study with previous analyses of the same data. I support publication of the manuscript, including the human data, which as the authors point out, do add substantially to the available data for MPM.

We are extremely grateful to this reviewer for her/his appreciation of our work and effort spent in this revision.

C4. *Regarding the low allelic frequency of KRAS mutations noted in the study, I wondered if the authors could add an analysis of whole-genome doubling. It occurred to me that wGD might be enriched in the KRAS/TP53 altered patient cohort and that this might explain the low allelic frequency of KRAS. Further, if wGD precedes KRAS mutation then the presence of more KRAS alleles might make KRAS mutation acquisition more likely.*

We are extremely grateful to this reviewer for her/his idea and attempted to find ways in order to perform such analyses, however we were unable to, since we do not possess whole genome or transcriptome sequencing data. We mention this great idea of this Reviewer in the discussion section as a proposal for further future study of our findings.

C5. *How did the authors select the one MAPED trial patient for investigation? This is important information to include given the patient turned out to have 4 KRAS mutations and TP53 alteration.*

The MAPED study was an observational trial that did not include sample biobanking. The single patient included was one single mesothelioma patient out of 15 others who contacted us independently and wished to donate samples for research. We will be happy to eliminate the patient data from the paper in the light of the new patients from Turkey, if the reviewer wishes so (of course, we would prefer to keep the data in the paper).

C6. *The immunohistochemistry performed for BAP1 does not "validate" (p. 12) the BAP1 mutations found using sequencing approaches since it shows that BAP1 protein remains present and in the expected location in these cell lines. In MPM, it is thought that loss of BAP1 nuclear protein correlates with functionally significant BAP1 mutations (Nasu et al., 2015, JTO). This should be re-worded in the results text.*

We apologize for our mistake and we corrected and referenced as appropriate.

12th Oct 2021

Dear Dr. Stathopoulos,

Thank you for the submission of your revised manuscript to EMBO Molecular Medicine. We have received the enclosed reports from referees #2 and #3 who re-reviewed your manuscript, and as you will see, they are now supportive of publication. Nevertheless, while the referees agree that your functional studies support the hypothesis of a role of KRAS in mesothelioma, the analysis of existing data remains unclear to them. We therefore consulted a biostatistician on this aspect, who also considered that the manual curation of existing datasets was not sufficiently well described. Therefore, in a minor revision of your manuscript, we would like you to clearly describe the procedure you followed (i.e. did you tally the number of entries reflecting mutations? When you mention downloading data from public databases, did you download vcf files or did you recall variants using the raw reads and find mutations others did not?), as well as suggest explanation on why previous efforts have not identified/reported these mutations.

Moreover, please also address the following minor editorial points:

1/ Main manuscript text:
- Please answer/correct the changes suggested by our data editors in the main manuscript file (in track changes mode). This file will be sent to you in the next few days. Please use this file for any further modification.
- Please remove the red text, and only keep in track changes mode any further modification.
- Material and methods:
o Human studies: please include the full statement that the experiments conformed to the principles set out in the WMA Declaration of Helsinki and the Department of Health and Human Services Belmont Report.
o Cells: please indicate the origin of the cells.
o As we don't have size limitation, please include the methods from the Appendix in the main manuscript text.
- Data Availability section: Thank you for providing a DAS. Please note that the datasets must be publicly available before online publication.

2/ Figures:
- Please indicate in the main and appendix figures or in their legends the exact n and p= values (including for non-significant p values, ns), not a range, along with the statistical test used.

3/ Thank you for providing Source Data. Please add the tab that contains data for fig 2 and currently in Source Data fig.1 in the Source data for fig. 2 as well.
Please check the labelling of the tabs for Source Data Fig. 3.
Please check the file currently labelled "Source Data Fig. EV1, EV2 and EV3".
Source Data Fig. 9 contains a personal email in .txt format, please remove.

4/ Synopsis:
Thank you for providing a synopsis text. I had to shorten it a bit to fit our format, please let me know if you agree with the following:
Human malignant pleural mesothelioma (MPM) is a devastating occupational cancer that kills more than 200000 individuals annually. Some MPM harbor mutations of the KRAS proto-oncogene, but their functional significance is unknown and experimental models for these MPM are not available.
• Activating mutations, amplifications, and transcriptional activation of KRAS alone or in combination with inactivating alterations of TP53 are found in 20% of 86 MPM patients from the TCGA dataset.
• KRAS mutations alone or in combination with TP53 loss are corroborated in 40-50% of 35 newly reported MPM patients and of 33 established MPM cell lines.
• Mesothelial-restricted ectopic expression of KRASG12D alone or in combination with deletion of the murine TP53 genes are shown to trigger epithelioid or biphasic MPM, respectively, with 100% penetrance.
• Three MPM cell lines are provided, which feature the causative KRAS and TP53 mutations but also secondary mutations in the mouse BAP1 gene, as well as a human MPM-like transcriptome.
• Transgenic and transplantable MPM models for hypothesis testing and drug triage are provided and the prototype KRAS inhibitor deltarasin is shown to block MPM development in mice by 76%.

Thank you for providing a picture as well. Could you please add some text for better comprehension and to reflect your findings?

I look forward to receiving your revised manuscript.

With kind regards,

Lise Roth

Lise Roth, PhD
Editor
EMBO Molecular Medicine

***** Reviewer's comments *****

Referee #2 (Comments on Novelty/Model System for Author):

Look, the authors tried and they got some human specimens that allowed them to confirm the presence of RAS mutations. The issue remains, why previous authors missed them. This question could have been answered by establishing a collaboration with Bueno, Ladany and others who previously analyzed MM by WES RNAseq, etc., and did not detect RAS mutations. I would have done that, i wouldhave liked to see my data independently validated before saying that others had missed those mutations, but that is my point of view, they are entitled to a different point of view. In short, I think that they seem careful and convinced their data are correct: so the data should be published and then we we'll see if the data are independently validated. Hopefully so. Please note that I am not a biostatistician so I cannot comment on that. I hope one of the other reviewers was a biostatistician.

Referee #2 (Remarks for Author):

The authors did the effort to get more specimens and validated the presence of KRAS in 9/23 additional mesothelioma biopsies, 6 from France and 23 from Turkey and 3 more had combined KRAS/TP53 mutations.

Referee #3 (Remarks for Author):

The authors have adequately addressed my concerns and added new data in independent patients.

The authors performed the requested editorial changes.

Dear Dr. Stathopoulos,

Thank you for the submission of your revised manuscript to EMBO Molecular Medicine. I am sorry for the delay in getting back to you, as we were waiting for the input of our advisor on the biostatistic aspect of the study. This advisor has now gotten back to us and is satisfied with your responses but would nevertheless like you to back up your argumentation with references. We would therefore like you to add a reference for the following sentence:
“The fact that these mutations were not detected by most next generation sequencing studies of MPM can be explained by the relative low sensitivity of these methods compared with ddPCR, as well as the low allelic frequency of KRAS mutations. To this end, typical read depths of 50-100 are employed in most next generation sequencing studies yielding a sensitivity of 1-2%, compared with that of 0.005% of ddPCR [REFS]. In addition, most next generation sequencing studies set discovery cut-offs of 25% allelic frequency, likely rendering many KRAS mutations undiscovered.”

Once this is done, please accept all changes. You may send me the final manuscript via email.

Please also include a legend in the synopsis picture that you may also send me via email.

I look forward to receiving these files.

With kind regards,

Lise Roth

Lise Roth, PhD
Editor
EMBO Molecular Medicine

15th Nov 2021

Dear Dr. Stathopoulos,

Thank you for submitting the revised files. Please accept my apologies for the confusion regarding the synopsis image. I meant to add a legend within the picture. I have now done it myself, please check the attached picture and let me know if you approve.

I am pleased to inform you that your manuscript is accepted for publication and is now being sent to our publisher to be included in the next available issue of EMBO Molecular Medicine.

Congratulations on your interesting work,

With kind regards,

Lise Roth

Lise Roth, Ph.D
Scientific Editor
EMBO Molecular Medicine

EMBO PRESS

YOU MUST COMPLETE ALL CELLS WITH A PINK BACKGROUND

PLEASE NOTE THAT THIS CHECKLIST WILL BE PUBLISHED ALONGSIDE YOUR PAPER

Corresponding Author Name: GEORGIOS STATHOPOULOS	
Journal Submitted to: EMBO MOLECULAR MEDICINE	
Manuscript Number: 13631.R1	

USEFUL LINKS FOR COMPLETING THIS FORM

http://www.antibodypedia.com	Antibodypedia
http://1degreebio.org	1DegreeBio
http://www.equator-network.org/reporting-guidelines/improving-bioscience-research-repor	ARRIVE Guidelines
http://grants.nih.gov/grants/olaw/olaw.htm	NIH Guidelines in animal use
http://www.mrc.ac.uk/Ourresearch/Ethicsresearchguidance/Useofanimals/index.htm	MRC Guidelines on animal use
http://ClinicalTrials.gov	Clinical Trial registration
http://www.consort-statement.org	CONSORT Flow Diagram
http://www.consort-statement.org/checklists/view/32-consort/66-title	CONSORT Check List
http://www.equator-network.org/reporting-guidelines/reporting-recommendations-for-tumo	REMARK Reporting Guidelines (marker prognostic studies)
http://datadryad.org	Dryad
http://figshare.com	Figshare
http://www.ncbi.nlm.nih.gov/gap	dbGAP
http://www.ebi.ac.uk/ega	EGA
http://biomodels.net/	Biomodels Database
http://biomodels.net/miriam/	MIRIAM Guidelines
http://jjj.biochem.sun.ac.za	JWS Online
http://oba.od.nih.gov/biosecurity/biosecurity_documents.html	Biosecurity Documents from NIH
http://www.selectagents.gov/	List of Select Agents

Reporting Checklist For Life Sciences Articles (Rev. June 2017)

This checklist is used to ensure good reporting standards and to improve the reproducibility of published results. These guidelines are consistent with the Principles and Guidelines for Reporting Preclinical Research issued by the NIH in 2014. Please follow the journal's authorship guidelines in preparing your manuscript.

A- Figures
1. Data
The data shown in figures should satisfy the following conditions:

- the data were obtained and processed according to the field's best practice and are presented to reflect the results of the experiments in an accurate and unbiased manner.
- figure panels include only data points, measurements or observations that can be compared to each other in a scientifically meaningful way.
- graphs include clearly labeled error bars for independent experiments and sample sizes. Unless justified, error bars should not be shown for technical replicates.
- if n< 5, the individual data points from each experiment should be plotted and any statistical test employed should be justified
- Source Data should be included to report the data underlying graphs. Please follow the guidelines set out in the author ship guidelines on Data Presentation.

2. Captions
Each figure caption should contain the following information, for each panel where they are relevant:

- a specification of the experimental system investigated (eg cell line, species name).
- the assay(s) and method(s) used to carry out the reported observations and measurements
- an explicit mention of the biological and chemical entity(ies) that are being measured.
- an explicit mention of the biological and chemical entity(ies) that are altered/varied/perturbed in a controlled manner.
- the exact sample size (n) for each experimental group/condition, given as a number, not a range;
- a description of the sample collection allowing the reader to understand whether the samples represent technical or biological replicates (including how many animals, litters, cultures, etc.).
- a statement of how many times the experiment shown was independently replicated in the laboratory.
- definitions of statistical methods and measures:
  - common tests, such as t-test (please specify whether paired vs. unpaired), simple χ2 tests, Wilcoxon and Mann-Whitney tests, can be unambiguously identified by name only, but more complex techniques should be described in the methods section;
  - are tests one-sided or two-sided?
  - are there adjustments for multiple comparisons?
  - exact statistical test results, e.g., P values = x but not P values < x;
  - definition of 'center values' as median or average;
  - definition of error bars as s.d. or s.e.m.

Any descriptions too long for the figure legend should be included in the methods section and/or with the source data.

In the pink boxes below, please ensure that the answers to the following questions are reported in the manuscript itself. Every question should be answered. If the question is not relevant to your research, please write NA (non applicable). We encourage you to include a specific subsection in the methods section for statistics, reagents, animal models and human subjects.

B- Statistics and general methods

Please fill out these boxes (Do not worry if you cannot see all your text once you press return)

1.a. How was the sample size chosen to ensure adequate power to detect a pre-specified effect size?	An a priori target sample size of n=10/group was calculated using G* power (http://www.gpower.hhu.de/) assuming α-error=0.05, β-error=0.95, and Cohen's d=1.5 resulting from 30%differences between groups featuring SD spanning 20-30% of the mean. Page MS24.
1.b. For animal studies, include a statement about sample size estimate even if no statistical methods were used.	An a priori target sample size of n=10/group was calculated using G* power (http://www.gpower.hhu.de/) assuming α-error=0.05, β-error=0.95, and Cohen's d=1.5 resulting from 30%differences between groups featuring SD spanning 20-30% of the mean. Page MS24.
2. Describe inclusion/exclusion criteria if samples or animals were excluded from the analysis. Were the criteria pre-established?	No data were excluded. Page MS24
3. Were any steps taken to minimize the effects of subjective bias when allocating animals/samples to treatment (e.g. randomization procedure)? If yes, please describe.	Since in animal study the sample size is relatively low as compared to clinical studies, randomization has limited applicability. Instead we used simple alternation of littermates to treatments or interventions. After alternation and before experimentation, baseline characteristics of the experimental groups, such as body mass, were analyzed to ensure there is no statistically significant differences between the experimental groups at baseline. Page MS24.
For animal studies, include a statement about randomization even if no randomization was used.	Since in animal study the sample size is relatively low as compared to clinical studies, randomization has limited applicability. Instead we used simple alternation of littermates to treatments or interventions. After alternation and before experimentation, baseline characteristics of the experimental groups, such as body mass, were analyzed to ensure there is no statistically significant differences between the experimental groups at baseline. Page MS24.
4.a. Were any steps taken to minimize the effects of subjective bias during group allocation or/and when assessing results (e.g. blinding of the investigator)? If yes please describe.	Data acquisition was blinded on samples previously coded by a non- blinded investigator. Page MS24.
4.b. For animal studies, include a statement about blinding even if no blinding was done	Data acquisition was blinded on samples previously coded by a non- blinded investigator. Page MS24.
5. For every figure, are statistical tests justified as appropriate?	Yes. Pages MS 46-64.
Do the data meet the assumptions of the tests (e.g., normal distribution)? Describe any methods used to assess it.	All data were examined for normality by Kolmogorov-Smirnov test and were normally distributed. Page MS24.
Is there an estimate of variation within each group of data?	Values are given as mean ± 95% confidence interval. Page MS23.
Is the variance similar between the groups that are being statistically compared?	Yes. Page MS23.

C- Reagents

6. To show that antibodies were profiled for use in the system under study (assay and species), provide a citation, catalog number and/or clone number, supplementary information or reference to an antibody validation profile. e.g., Antibodypedia (see link list at top right), 1DegreeBio (see link list at top right).	Yes, we provide catalogue numbers, providers and dilutions. Appendix Table S5. Page S10.
7. Identify the source of cell lines and report if they were recently authenticated (e.g., by STR profiling) and tested for mycoplasma contamination.	All cell lines used are identified by source and/or citation. Appendix Page 3 and MS Pages 19 and 22. Cell lines were tested annually for identity (by the short tandem repeat method) and for Mycoplasma Spp. (by PCR). Page MS21 and Appendix Page S3.

* for all hyperlinks, please see the table at the top right of the document

D- Animal Models

8. Report species, strain, gender, age of animals and genetic modification status where applicable. Please detail housing and husbandry conditions and the source of animals.	C57BL/6 (#000664), B6.129(Cg)-Gt(ROSA)26Sortm4(ACTB-tdTomato,-EGFP)Luo/J (mT/mG; #007676; Muzumdar et al, 2007), FVB-Tg(CAG-luc,-GFP)L2G85Chco/J (CAG.Luc.eGFP; #008450; Cao et al, 2004)64, B6.129S4-Krastm4Tyj/J (KRASG12D; #008179; Jackson et al, 2001), and B6.129P2-Trp53tm1Brn/J (Trp53f/f; #008462; Meylan et al, 2009) mice were obtained from Jackson Laboratories (Bar Harbor, ME) and bred on the C57BL/6 background at the University of Patras Center for Animal Models of Disease. Pages MS21-22.
9. For experiments involving live vertebrates, include a statement of compliance with ethical regulations and identify the committee(s) approving the experiments.	Experiments were carefully designed and approved a priori by the Prefecture of Western Greece's Veterinary Administration (approval 118018/578-30.04.2014), and were conducted according to Directive 2010/63/EU (http://eur-lex.europa.eu/legal-content/EN/TXT/?uri=CELEX%3A32010L0063). Sex-, weight (20-25 g)-, and age (6-12 week)-matched experimental mice were used, and their numbers (total n = 432) are detailed in Appendix Table S6, Appendix Page S11.
10. We recommend consulting the ARRIVE guidelines (see link list at top right) (PLoS Biol. 8(6), e1000412, 2010) to ensure that other relevant aspects of animal studies are adequately reported. See author guidelines, under 'Reporting Guidelines'. See also: NIH (see link list at top right) and MRC (see link list at top right) recommendations. Please confirm compliance.	Yes.

E- Human Subjects

11. Identify the committee(s) approving the study protocol.	Ludwig-Maximilians-University Munich Ethics Committee (approvals #623–15 and #711–16) University of Patras Ethics Committee (approval #22699/21.11.2013). French Ministry of Research (DC-2011-1399). MS19-21.
12. Include a statement confirming that informed consent was obtained from all subjects and that the experiments conformed to the principles set out in the WMA Declaration of Helsinki and the Department of Health and Human Services Belmont Report.	The Munich clinical study was conducted in accordance with the Helsinki Declaration and was prospectively approved by the Ludwig-Maximilians-University Munich Ethics Committee (approvals #623–15 and #711–16). All patients gave written informed consent a priori. The Patras clinical study (Clinical identification of malignant pleural effusions in the emergency department, MAPED) entailed samples from patients enrolled in a prospective clinical trial (Stathopoulos et al, 2020). MAPED was conducted in accord with the Declaration of Helsinki, was registered with ClinicalTrials.gov (#NCT03319472), and written informed consent was obtained from all patients a priori. MAPED was approved by the University of Patras Ethics Committee (approval #22699/21.11.2013). The Nantes MPM cell lines were derived from pleural fluid aspirates obtained for diagnostic purposes. All patients gave written informed consent a priori for their excess pleural fluid not required for diagnosis to be used for the establishment of cell lines. MPE samples from 61 patients with MPM were used to generate the cell lines, as described elsewhere (Gueugnon et al, 2011; Delaunay et al, 2020). All the collected samples and the associated clinical information were registered in a database validated by the French Ministry of Research (DC-2011-1399). Pages MS19-21.
13. For publication of patient photos, include a statement confirming that consent to publish was obtained.	NA
14. Report any restrictions on the availability (and/or on the use) of human data or samples.	NA
15. Report the clinical trial registration number (at ClinicalTrials.gov or equivalent), where applicable.	The Patras clinical study was registered with ClinicalTrials.gov (#NCT03319472). Page MS21.
16. For phase II and III randomized controlled trials, please refer to the CONSORT flow diagram (see link list at top right) and submit the CONSORT checklist (see link list at top right) with your submission. See author guidelines, under 'Reporting Guidelines'. Please confirm you have submitted this list.	NA
17. For tumor marker prognostic studies, we recommend that you follow the REMARK reporting guidelines (see link list at top right). See author guidelines, under 'Reporting Guidelines'. Please confirm you have followed these guidelines.	NA

F- Data Accessibility

18: Provide a "Data Availability" section at the end of the Materials & Methods, listing the accession codes for data generated in this study and deposited in a public database (e.g. RNA-Seq data: Gene Expression Omnibus GSE39462, Proteomics data: PRIDE PXD000208 etc.) Please refer to our author guidelines for 'Data Deposition'.<br><br>Data deposition in a public repository is mandatory for:<br>a. Protein, DNA and RNA sequences<br>b. Macromolecular structures<br>c. Crystallographic data for small molecules<br>d. Functional genomics data<br>e. Proteomics and molecular interactions	Yes we provide a data availability section at the end of Materials and Methods Section. Page MS 31.
19. Deposition is strongly recommended for any datasets that are central and integral to the study; please consider the journal's data policy. If no structured public repository exists for a given data type, we encourage the provision of datasets in the manuscript as a Supplementary Document (see author guidelines under 'Expanded View' or in unstructured repositories such as Dryad (see link list at top right) or Figshare (see link list at top right).	Yes we provide a data availability section at the end of Materials and Methods Section. Page MS31.
20. Access to human clinical and genomic datasets should be provided with as few restrictions as possible while respecting ethical obligations to the patients and relevant medical and legal issues. If practically possible and compatible with the individual consent agreement used in the study, such data should be deposited in one of the major public access-controlled repositories such as dbGAP (see link list at top right) or EGA (see link list at top right).	NA
21. Computational models that are central and integral to a study should be shared without restrictions and provided in a machine-readable form. The relevant accession numbers or links should be provided. When possible, standardized format (SBML, CellML) should be used instead of scripts (e.g. MATLAB). Authors are strongly encouraged to follow the MIRIAM guidelines (see link list at top right) and deposit their model in a public database such as Biomodels (see link list at top right) or JWS Online (see link list at top right). If computer source code is provided with the paper, it should be deposited in a public repository or included in supplementary information.	Human MPM mutation data were retrieved from the cBioPortal for Cancer Genomics (www.cbioportal.org/) by querying "mesothelioma", selecting the study "Mesothelioma (TCGA, PanCancer Atlas)", selecting "Query by Gene", submitting KRAS and TP53, selecting "Mutations", "Putative copy-number alterations from GISTIC", "mRNA expression z-scores", and "Protein expression z-scores", and by selecting "Submit Query". Raw data were downloaded (Source Data for Figs 1C-E and 2B-F) and analyzed. Gene expression data from 86 TCGA MPM patients (one patient without KRAS/TP53 alterations was not available) were downloaded from https://xenabrowser.net/datapages/?dataset=TCGA-MESO.htseq_fpkm-uq.tsv&host=https%3A%2F%2Fgdc.xenahubs.net&removeHub=https%3A%2F%2Fxena.treehouse.gi.ucsc.edu%3A443 and were analyzed using R*. ENSEMBL gene IDs were converted to gene symbols using biological database network (https://biodbnet-abcc.ncifcrf.gov/db/db2dbRes.php). The data were filtered, normalized, and differential gene expression (ΔGE) was computed using the packages limma (https://bioconductor.org/packages/release/bioc/html/limma.html) and edgeR<br><br>(https://bioconductor.org/packages/release/bioc/html/edgeR.html) on R* using a divisive K-means approach. All mutations (n = 2150) of all patients (n = 87) with MPM from the TCGA pan-cancer dataset were retrieved from www.cbioportal.org/ and were fed into the protein analysis through evolutionary relationships (PANTHER) Classification System (www.pantherdb.org/) using parameters: organism, Homo Sapiens; analysis, statistical overrepresentation test>PANTHER pathways or reactome pathways (both analyses were done); whole-genome reference list: Homo Sapiens; test type: binomial; and correction: false discovery rate. All raw data from the two independent PANTHER and reactome pathway analyses were retrieved, merged, and analyzed. Gene set enrichment analysis (GSEA) was performed with the Broad Institute pre-ranked GSEA module software (http://software.broadinstitute.org/gsea/index.jsp; Subramanian et al, 2005). MS pages 18-19.

G- Dual use research of concern

22. Could your study fall under dual use research restrictions? Please check biosecurity documents (see link list at top right) and list of select agents and toxins (APHIS/CDC) (see link list at top right). According to our biosecurity guidelines, provide a statement only if it could.	No
